# Examining local level variation in Special Educational Needs and Disabilities (SEND) service provision and associated data sources in England: a scoping review

**DOI:** 10.1057/s41599-025-06319-0

**Published:** 2026-02-10

**Authors:** Jennifer Clare Saxton, Ariadna Albajara Saenz, Owen Williams, Jacob Matthews, Isaac Winterburn, Eleanor Chatburn, Nazneen Nazeer, Kristine Black-Hawkins, Tamsin Ford

**Affiliations:** 1 University of Cambridge, Cambridge, UK; 2 University of East Anglia, Norwich, UK; 3 University of Kelaniya, Kelaniya, Sri Lanka

**Keywords:** Information systems and information technology, Health humanities, Education, Science, technology and society, Social policy

## Abstract

The Special Educational Needs and Disabilities (SEND) system in England was reformed in 2014, including the introduction of child-centred legally binding education, health and care plans (EHCPs) and expectations for improved multi-agency working coordinated by local authorities (LAs). Since then, service-user satisfaction has declined, and children’s outcomes have not improved. Critics cite local variation in SEND provision and limited accountability for implementation failures as contributing factors. This scoping review summarises peer-reviewed and grey literature as well as open-access data sources reporting LA and multi-academy trust (MAT) level variation in SEND provision to identify key reasons for local variation SEND services, and data sources that could be used to better monitor local public bodies. We identified and graded 120 articles/reports (18 peer-reviewed, 102 grey) and 8 open-access data sources. The literature investigated nine topic areas; most studies were low quality. Eight open-access data sources included information about SEND prevalence, demand and provision complaints. Our review highlights that LAs were underprepared and under-resourced to implement the reforms. The administration involved in EHCPs contribute to variation in multiple ways. Greater standardisation and guidance for professionals could reduce variation. Existing data sources could be better used to improve monitoring and accountability for local provision, which should include MATs given their potential to influence SEND provision. Low-quality studies dominated the grey literature. More rigorous methods and reporting standards could also improve decision-making and service planning.

## Introduction

### Background and policy context

In England, children and young people (CYP) who experience significantly greater difficulties in learning than their peers, or disabilities that hinder their use of standard facilities in mainstream education settings, are said to have special educational needs (SEN) (DfE & DHSC, 2015). Though disabilities do not necessarily involve learning difficulties, government policy combines ‘SEN’ and disabilities into the term ‘SEND’, recognising that some CYP will require additional support to thrive in education settings in the absence of a primary learning difficulty. In this review, we will use the term ‘SEND’ when referring to the English system, and unless otherwise specified we will use the term ‘SEN’ for other countries. SEND provision in England aims to improve CYP’s access to education, while supporting their health and wellbeing (DfE & DHSC, 2015). SEND policy is underpinned by the principle of inclusion, and the removal of barriers to learning to enable the majority of CYP with SEND to be educated in mainstream settings (DfE & DHSC, 2015). This inclusion principle has been adopted by many countries globally, increasingly so since international declarations such as the 2006 convention on the rights of persons with disabilities (Buchner et al., 2021; United Nations, 2006). Whilst definitions and operationalisation of SEN and educational inclusion vary by country, lack of consistent improvement to CYP outcomes relative to peers without SEN are widespread (Dalgaard et al., 2022). There is also limited high quality research to unpick the reasons for lack of overall positive impact, such as few studies consulting CYP and an overreliance on qualitative and cross-sectional studies to inform practice (Keles et al., 2024). Whilst the Organisation for Economic Co-operation and Development (OECD) has made efforts to compile SEN data from signatory countries to help overcome commonly experienced challenges in providing SEN-related support (OECD, 2005), data gaps remain that, if filled, could help countries to refine policies and systems to improve experiences and outcomes of CYP with SEN. Greater use of administrative data for example, could contribute to SEN policy analyses (Gilmour & Stiefel, 2025).

The way in which CYP and their families can seek SEND-related support for education underwent fundamental legislative and policy change in England in 2014 (UK Government, 2014). Key changes included new legal routes for redress if SEND provision fell short of statutory expectations, expansion of age eligibility for services from 18 to 25 and replacing non-enforceable ‘statements’ with legally binding Education, Health and Care Plans (EHCPs).

EHCPs are intended for CYP with high support needs that schools cannot meet with ordinarily available provision. 5.3% of pupils in state-funded settings had an EHCP in 2024/25, most commonly for autism spectrum disorder (ASD) (DfE, 2025). EHCPs may reflect combined inputs from health, social care and education sectors, for example specialised mental health support, interventions for complex communication needs, education within a specialist setting, and the assignment of disability social workers (DfE & DHSC, 2015). EHCP eligibility assessments, plan development and reviews are overseen by local authorities (local public bodies responsible for delivering public services). The transition to EHCPs aimed to provide a more satisfying process for parents/carers and better outcomes for CYP, through a child-centred approach, greater involvement of parents/carers in decision-making, and integrated input from health, education and social care sectors to enable more holistically designed support (Sales & Vincent, 2018).

The remainder of CYP with SEND are assigned ‘SEN Support’ (14.2% of pupils in state-funded settings in 2024/25; (DfE, 2025), which is overseen by schools. Examples include small group working, the provision of extra time in exams, or short-term speech and language therapy. A ‘Local Offer’ (LO) for SEND was also introduced to provide information about local SEND services, allow service-users to give feedback, and shape services to better suit local needs (DfE & DHSC, 2015).

### Current performance of the SEND system in England and other high-income settings

More than a decade after the implementation of England’s SEND legislation, there is growing evidence that it has not met its goals of improving parent/carer satisfaction or outcomes for CYP (National Audit Office, 2019). Poor outcomes are being compounded by increasing numbers of CYP with identified needs (DfE, 2025) reduced resources to meet them, and a system that is described as financially unsustainable (National Audit Office, 2024).

SEND-related complaints to the Local Government and Social Care Ombudsman and appeals against local authority (LA) decisions regarding SEND assessment and provision are at record levels, with most cases being upheld in favour of families (LGSCO, 2023; MoJ, 2023). Educational outcomes for CYP with SEND remain considerably worse than those for their peers without SEND (DfE, 2020, 2022a). Recent time trend analyses showed modest narrowing of attainment gaps at ages 11, 16 and 16–25, but a widening attainment gap at age 5 for CYP receiving SEN support or EHCPs (Hunt, 2025). Onward trajectories of CYP with SEND are also poorer, including disproportionately higher rates of school exclusion (Timpson, 2019) and increased risk of becoming ‘not in employment education or training’ (NEET) (Crowley, 2023). An evaluation of 30 local area inspections concluded that CYP have not benefitted sufficiently since the introduction of the reforms, and that in the weakest local areas, there was a lack of strategic oversight to implement the reforms successfully (OFSTED, 2017; Tysoe et al., 2021). Independent reports by the National Audit Office have also found that the system offers poor value for money, suffers from low financial sustainability, and fails to adequately support the needs of CYP with SEND and their families (National Audit Office, 2019, 2024). Research examining outcomes across different geographical areas has identified significant variation in academic attainment, suggesting a ‘postcode lottery’ in SEND provision (postcodes refer to specific geographical areas; the lottery component refers to unfair variation in the availability of services based purely on where you live) (Azpitarte & Holt, 2024). The government has acknowledged this variation, expressing a commitment to providing ‘the right support in the right place at the right time’ for all children with SEND, partly by addressing variation in service quality and access across local areas (DfE, 2022b).

The downward trend in SEND service performance has occurred against a background of increasing austerity and key policy changes including the push for all schools to become multi-academy trusts (MATs) (Gray & Barford, 2018; Roberts, 2022). MATs are single governing bodies with charitable status that oversee the management of groups of schools (DfE, 2021); the average MAT includes 7–8 schools but there is a wide range (Plaister, 2023). MATs are independent of LAs in terms of accountability and control (unlike schools that are LA-maintained), and are afforded greater flexibility in their curriculum, timetables, and staff working arrangements (DfE, 2021). The impact of MATs on SEND provision and outcomes remains an under-researched area, but some Special Educational Needs Coordinators (SENCos) report added value, particularly for MATs with dedicated SEND leaders (Flemons, 2024). In contrast, notable concerns about MATs include lower than expected rates of SEND identification in areas with the highest proportions of academised primary schools compared to LAs with the fewest academies (Hutchinson, 2025). There are also higher than average rates of permanent exclusion and unexplained exits from schools, exceeding those of LA-maintained schools, with three-quarters of affected pupils considered vulnerable, including those with SEND (Hutchinson, 2019). LAs also contribute to variation in SEND identification and provision (Hutchinson, 2025). They play a central role in SEND provision, including decisions about accepting applications for EHC assessments and determining the final contents of EHCPs, including which setting a child will attend.

The challenges faced in England (whose education system is centrally managed by the UK government) are mirrored in many other high-income settings. For example, the adjacent devolved nation of Scotland, which has separate education policies, regulations and systems to England, has seen an eight-fold increase in CYP receiving ‘additional support needs’ (ASL) since their 2004 reforms. There is also evidence of wide gaps in attainment for CYP receiving ASL compared to those who do not, and critics say that poor data collection and recording have contributed to inadequate planning and financing for ASL (Audit Scotland, 2025). Another example is Finland, which shares a similar tiered approach to SEN provision with England. Though Finland is widely regarded as having one of the best education systems in the world, it too stands on the brink of further SEN reform to improve outcomes and reduce inequity, based on recent OECD analyses (OECD, 2022).

### Rationale for this review

Despite the policy in England for all schools to become part of MATs, we are not aware of any evidence reviews examining how MATs may be affecting SEND identification and provision. Similarly, although a few studies have mentioned a ‘postcode lottery’ in SEND provision at LA level, we could not find any comprehensive reviews assessing LA-level variation since 2014. Our review is particularly timely given the recent change of government, and the potential for further reforms, in light of the current government enquiry into ‘Solving the SEND crisis’ (UK Parliament, 2024) and the delayed Schools White Paper which is now expected in 2026 (HM Treasury, 2025). Findings from this review will also be relevant to other high-income countries with a similar focus on inclusion of SEN learners. Several countries stand on the brink of SEN reform – or have recently implemented reforms and are in the process of their own evaluations (Cameron et al., 2024). Many high-income settings are recording increased rates of SEN amongst their pupils, including specific conditions such as autism, which often require additional funding and systems to provide additional support in schools (Solmi et al., 2022). This is occurring amidst a global teacher crisis, affecting all countries (UNESCO, 2024), and a push by many governments to ensure better outcomes for CYP with SEN, e.g. in Northern Ireland (Department for Education Northern Ireland, 2025), Japan (Kaizu & Tamaki, 2024), and many others.

This review forms part of The HOPE Study, which is using England-wide linked administrative health and education data to examine outcomes for CYP following SEND-related provision (Zylbersztejn et al., 2023) We conducted the review to better understand the factors contributing to variation in SEND identification, provision and outcomes at both LA and MAT levels observed in administrative data, as well as to identify additional data sources that could be used to monitor LA and MAT-level variation. Whilst several open-access data sources with SEND-related information are available on the Department for Education (DfE) website (DfE, 2024), it is not clear whether this list is comprehensive or is being used to its full potential to monitor LA or MAT-level performance, such as via local government online tools (LGA, 2025). This would be useful given that local area inclusion dashboards have been considered to improve accountability for service provision. Findings from our review will inform evidence-based recommendations for reducing variability within the SEND system, offer suggestions for better use of existing data to monitor variation, and outline research priorities to further improve understanding of how, why and for whom SEND identification and provision varies.

### Review aims and objectives

We aimed to summarise evidence and open-access data sources that include information about SEND identification and provision at LA and MAT levels in England.

Our objectives were to:

1. Identify, summarise and grade evidence from peer-reviewed and grey literature sources documenting any aspect of SEND identification and provision at LA and MAT levels.
2. Identify and summarise open-access data sources that include SEND-related information at LA and MAT levels.
3. Draw out key aspects of SEND services and key processes contributing to variation at LA and MAT levels.
4. identify remaining evidence gaps and make evidence-based recommendations to improve SEND identification and provision at LA and MAT levels.

## Methods

### Choice of review type and protocol

We chose to conduct a scoping review because this method is designed to synthesise and ‘map’ the literature on a particular topic or research area, and to identify research gaps that can inform policy and practice (Daudt et al., 2013). A scoping review was well suited to our broad lines of enquiry focused on mapping variation and data sources at LA and MAT levels.

### Eligibility criteria for included studies—literature review

We included quantitative and qualitative studies from grey and peer-reviewed literature reporting data on any aspect of SEND identification or provision at LA or MAT levels in England, written in English. Example areas of investigation included, but were not limited to, statutory EHC assessments, EHCP design processes, SEND staff training, and school types. Examples of data and information sources included SEND prevalence surveys, service-user and/or service-provider surveys, audits, annual reports and budgets, and value-for-money indicators. To be included, studies had to provide data for one or more LAs in England, which have legal responsibilities for SEND provision (see Online Resource 1). We excluded studies that: presented data collected prior to the introduction of the SEND Code of Practice (DfE & DHSC, 2015), aggregated data above or disaggregated below LA or MAT level, included uncoded qualitative data, focused on individuals older than 25 years of age, or reported only secondary data, unless the authors had conducted novel analyses.

### Eligibility criteria for included open-access data sources

We included open-access databases, report series, and interactive online tools containing any SEND-related data at LA or MAT levels.

### Search strategies

Our literature search covers the period from 1st January 2015 to 13th January 2022. As much of our review focuses on local government (LA) level variation, we considered it necessary to search both grey and peer-reviewed literature.

#### Search terms

For most of our searches, we developed five groups of search terms, which we combined using ‘AND’ and ‘OR’ operators, with parentheses as appropriate. The five groups were based on: (1) the level at which data were sought (i.e. LA or MAT), (2) the location (i.e. England), (3) the subject matter (SEND), (4) aspects of SEND provision for which LAs or MATs are responsible, and (5) our target age group (CYP aged 0–25). We restricted our search to articles published in English. See online resource 2 for full search terms for both peer-reviewed and grey literature.

#### Peer-reviewed databases

We searched Google Scholar and PubMed for medical, public health and education literature; PsycEXTRA for technical and government reports and conference papers; and the British Education Index for research, policy, and practice studies related to education and training in the UK.

#### Grey literature databases and other online sources

We searched the DfE website and the GOV.UK publications portal for official documents, command papers, House of Commons papers, and key departmental papers. We also searched ‘Local Offer for SEND’ websites via LA websites and hand-searched for studies exploring the views of parents, children and professionals on SEND provision, using each site’s internal search bar or the Google ‘Find' tool. For this search, we used the terms ‘consultation’, ‘feedback’ and ‘survey’. Due to the high number of results returned, we screened only the first 10 results per search. We also conducted an advanced Google search, set to retrieve PDFs from England and reviewed the first 20 pages of results (i.e. 200 documents). Finally, we consulted our stakeholder groups, who suggested additional websites potentially containing relevant grey literature: Special Needs Jungle (https://www.specialneedsjungle.com/), KIDS (https://www.kids.org.uk/sendiass), Mencap (https://www.mencap.org.uk/), Council for Disabled Children (https://councilfordisabledchildren.org.uk/). Our stakeholders included three advisory groups of CYP with SEND, parents/carers and professionals with a role in SEND provision which remained open to new membership for the study’s duration. We recruited stakeholders through our professional networks, and advertising in SEN journals and on social media. We consulted stakeholders throughout our study to ensure our research was relevant and meaningful to service users, and that our research tools were designed and worded appropriately.

#### Open access data sources

##### Search terms and databases/data sources

We searched for open-access data sources from the following online sources/websites: (1) GOV.UK, (2) the DfE’s Explore Education Statistics database, (3) the Local Government Association (the national membership body for local authorities), (4) the Local Government and Social Care Ombudsman, (5) the Family Hubs Network, (6) the Association of Directors of Children’s Services, (7) Ministry of Justice tribunal data, and (8) the register of schools and colleges in England. See Online Resource 3 for a summary of the open-access data source search strategy and associated URLs.

### Article screening and data extraction

Two reviewers screened the articles from the peer-reviewed literature, and a further two reviewers screened the grey literature. Both pairs of reviewers followed these steps in sequence: (1) Compilation of the list of references from each search; (2) Duplicate removal; (3) Title and abstract screening (for peer-reviewed literature), or in the case of the grey literature, an initial screen of the title and abstract (if available), followed by screening of the executive summary and contents page; (4) Full text review, with reviewers reporting reasons for any exclusions at this stage, and (5) Data extraction. Pairs of reviewers compared findings and resolved discrepancies with a third reviewer if necessary.

We documented the flow of literature through the review according to PRISMA guidelines (Page et al., 2021); see Online Resource 4. We developed a standardised table for data extraction from the included studies and reports (Online Resource 5).

### Quality grading of included studies

Although not routinely included in scoping reviews, two reviewers independently graded each study, with discrepancies resolved through discussion with a third reviewer. One purpose of the grading was to gauge the quality of the evidence that was influencing aspects of local SEND provision, such as the Local Offer for SEND. Secondly, we could use the study grades to provide greater emphasis on medium and high-quality studies in our results and recommendations arising.

Peer-reviewed literature: We used the Mixed Methods Appraisal Tool (MMAT) to critically appraise peer-reviewed literature (Hong et al., 2018) (see Online Resource 7). This tool was a pragmatic choice for oure review as it covers five study designs, alone or in combination for mixed-methods studies, allowing reviewers to use a single grading tool. As with our grey literature grading tool (see below), we assigned domain-specific and overall study grades based on the percentage of MMAT criteria met ('yes' judgements), in domains applicable to each study.

Grey literature: We used the AACODS checklist (Authority, Accuracy, Coverage, Objectivity, Date, Significance) to evaluate and critically appraise the grey literature (Tyndall, 2010). The checklist covers six domains (see Online Resource 6). For each domain, we graded studies as low ( < 50% of criteria met), medium (50-79%), or high ( > 79%). We then calculated a total quality score and assigned an overall quality grade (low, medium or high) to distinguish between lower- and higher-quality literature in our narrative summaries.

We based our scoring approach, and the alignment of MMAT and AACODS grades, on a prior systematic review that also used MMAT and AACODS in combination (Meulenbroeks et al., 2022).

### Categorisation of included studies and open access databases

We categorised the included studies according to the study designs, adapted from Nundy et al. (2022). In the absence of a precedent in the literature, we further grouped the studies according to their main area of investigation: (1) SEND identification and provision broadly/system-level, (2) EHCP processes, implementation and quality, (3) complaints and redress, (4) child voice and experience, (5) practitioner roles and training, (6) strategy and planning, (7) school exclusions, (8) transition to adulthood, and (9) the Local Offer for SEND. We classified the open-access data sources as follows: transport, funding, complaints, SEND provision, and EHCPs. Study categories were based on each study’s stated aim, what they did and what they reported that was relevant to the scoping review. Categories were developed by the lead author of the study who had oversight of the literature search and managed the master database of studies. Identifying main, and largely discrete areas of investigation was done for pragmatic reasons, enabling us to summarise a large and disparate set of studies and reports.

## Results

The first section of the results summarises the included literature from peer-reviewed and grey literature searches by topic area, study type and quality grade. The second section provides a narrative summary of the peer-reviewed and grey literature, and the third section summarises the open-access data sources, including SEND-related information and any available data trends since 2015. The final section summarises key SEND services and processes contributing LA and MAT-level variation.

### Records retrieved and included, and quality assessment

We included 18 peer-reviewed articles and 102 grey literature articles and reports (see Online Resource 4). A summary of the methodological characteristics and main areas of investigation is presented in Table 1.

**Table 1:** Methodological characteristics and main areas of investigation (*n* = 120 studies): sub-categories show n (%).

Study characteristic		Main area of investigation									
		SEND provision^a^	EHCPs^b^	Strategy & planning	Complaints & redressal	Child voice & experience	School exclusions	Transition to adulthood	Practitioner roles & training	Local offer for SEND	Total
Literature type	Peer-reviewed	3 (16.7)	5 (27.8)	0 (0)	3 (16.7)	2 (11.1)	1 (5.6)	2 (11.1)	2 (11.1)	0 (0)	18 (15)
	Grey	50 (49)	9 (8.8)	4 (3.9)	1 (1.0)	4 (3.9)	1 (1.0)	2 (2)	1 (1)	30 (29.4)	102 (85)
Study design^c^	Observational – Descriptive	48 (45.3)	11 (10.4)	3 (2.8)	2 (1.9)	5 (4.7)	1 (0.9)	3 (2.8)	3 (2.8)	30 (28.3)	106 (88.3)
	Observational – Analytical	5 (35.7)	3 (21.4)	1 (7.1)	2 (14.3)	1 (7.1)	1 (7.1)	1 (7.1)	0 (0)	0 (0)	14 (11.7)
Research method	Qualitative	7 (30.4)	4 (17.4)	1 (4.3)	1 (4.3)	2 (8.7)	2 (8.7)	2 (8.7)	1 (4.3)	3 (13.0)	23 (19.2)
	Quantitative (primary data)	6 (30)	7 (35)	0 (0)	0 (0)	2 (10)	0 (0)	1 (5)	1 (5)	3 (15)	20 (16.7)
	Quantitative (secondary data)	1 (33.3)	1 (33.3)	0 (0)	1 (33.3)	0 (0)	0 (0)	0 (0)	0 (0)	0 (0)	3 (2.5)
	At least two methods	39 (52.7)	2 (2.7)	3 (4.1)	2 (2.7)	2 (2.7)	0 (0)	1 (1.4)	1 (1.4)	24 (32.4)	74 (61.7)
Refers to	Local authorities	52 (43.7)	14 (11.8)	4 (3.4)	4 (3.4)	6 (5.0)	2 (1.7)	4 (3.4)	3 (2.5)	30 (25.2)	119 (99.2)
	Multi Academy Trusts	6 (75)	0 (0)	0 (0)	0 (0)	0 (0)	1 (12.5)	0 (0)	1 (12.5)	0 (0)	8 (6.7)
Respondents	Children and young people (CYP)	5 (31.25)	1 (6.25)	0 (0)	0 (0)	4 (25)	0 (0)	0 (0)	0 (0)	6 (37.5)	16 (13.3)
	CYP and Parents/carers	5 (62.5)	2 (25)	0 (0)	0 (0)	0 (0)	0 (0)	1 (12.5)	0 (0)	0 (0)	8 (6.7)
	Parents/carers	17 (56.7)	4 (13.3)	0 (0)	1 (3.3)	0 (0)	0 (0)	0 (0)	0 (0)	8 (26.7)	30 (25)
	Parents/carers & SEND/education professionals	1 (33.3)	1 (33.3)	0 (0)	1 (33.3)	0 (0)	0 (0)	0 (0)	0 (0)	0 (0)	3 (2.5)
	Education or care professionals	6 (54.5)	2 (18.2)	0 (0)	0 (0)	0 (0)	0 (0)	0 (0)	2 (18. 2)	1 (9.1)	12 (10)
	Mixed stakeholder group (3+ groups)	18 (37.5)	2 (4.2)	4 (8.3)	1 (2.1)	2 (4.2)	2 (4.2)	3 (6.25)	1 (2.1)	15 (31.3)	48 (40)
	n/a: secondary administrative or cohort data	1 (25)	2 (50)	0 (0)	1 (25)	0 (0)	0 (0)	0 (0)	0 (0)	0 (0)	4 (3.3)
Study quality^d^	Low	49 (47.6)	9 (8.7)	3 (2.9)	2 (1.9)	5 (4.8)	1 (1)	3 (2.9)	1 (1)	30 (29.1)	103 (85.8)
	Medium	2 (20)	3 (30)	1 (10)	1 (10)	1 (10)	1 (10)	0 (0)	1 (10)	0 (0)	10 (8.3)
	High	2 (28. 6)	2 (28.6)	0 (0)	1 (14.3)	0 (0)	0 (0)	1 (14.3)	1 (14.3)	0 (0)	7 (5.8)

^a^SEND provision: considered multiple aspects of SEND provision, identification and outcomes, and transition to the CFA 2014 legislation.

^b^EHCPs: Education and Health Care Plans.

^c^Study designs from Nundy et al. (2021); our review did not identify any experimental designs or meta-analyses.

^d^We used the AACODS checklist to grade grey literature (Tyndall, 2010) and the MMAT tool (Hong et al, 2018) to grade peer-reviewed literature.

### Peer-reviewed studies

Of the 18 included studies, 8 were qualitative, 3 analysed secondary data (using EHCPs as a data source or other administrative data), and 7 used at least two methods (analysing primary or secondary quantitative and qualitative data). All studies used observational designs: 13/18 were descriptive and 5/18 were analytical. Fifteen studies included results pertaining to LAs, one to MATs, and two to both LAs and MATs. The most investigated topic areas were EHCPs, complaints and redress, and broader aspects of SEND provision (see Table 1 and Online Resources 8 and 9). Using the MMAT quality assessment tool, 7 studies were judged to be of high quality, 6 of medium quality, and 5 of low quality (see Table 2). We report summary characteristics and quality grade for each peer-reviewed study in Online Resource 8 and provide the full list of included references in Online Resource 9.

**Table 2:** Percentage of studies graded as low, medium or high quality, by article type (peer-reviewed or grey literature) % (n).

Study domains graded	Study quality grade		
		
**Grey literature (*n*** = **102 studies) assessed with the ACCODS grading tool**^**1**^			
Section 1: Authority	12.7% (13)	73.5% (75)	13.7% (14)
Section 2: Accuracy	40.2% (41)	37.3% (38)	22.5% (23)
Section 3: Coverage	93.1% (95)	0% (0)	6.9% (7)
Section 4: Objectivity	18.6% (19)	49.0% (50)	32.4% (33)
Section 5: Date	18.6% (19)	49.0% (50)	32.4% (33)
Section 6: Significance	34.3% (35)	43.1% (44)	22.5% (23)
Overall study grade	96.1% (98)	3.9 (4)	0% (0)
**Peer-reviewed literature (*n*** = **18 studies) assessed with MMAT grading tool**			
Screening questions	0% (0)	0% (0)	100% (18)
Section 1 grade: Qualitative	6.7% (1)	46.7% (7)	46.7% (7)
Section 2 grade: Quantitative randomized controlled trials	N/A	N/A	N/A
Section 3 grade: Quantitative nonrandomized	0% (0)	0% (0)	100% (1)
Section 4 grade: Quantitative descriptive	22.2% (2)	55.6% (5)	22.2% (2)
Section 5 grade: Mixed methods	60.0% (3)	40.0% (2)	0% (0)
Overall study grade	27.8% (5)	33.3% (6)	38.9% (7)

1 ACCODS checklist, Tyndall, 2010

2 MMAT grading tool, Hong et al, 2018. Not all MMAT domains apply to all studies (they are study design dependent) so the denominator for calculating %s may be less than the total number of included studies.

Note: overall study grades cannot be higher than any individual domain grades.

### Grey literature studies

Of the grey literature studies, nearly half focused on SEND provision broadly, and one third focused on the Local Offer (LO) for SEND. Fifteen were qualitative, 20 involved quantitative primary data collection, and the remaining used at least two different methods. All studies used observational designs. Ninety-eight studies were rated as low quality, and four as medium quality. Most (*n* = 97) included reporting at the LA level only; the remainder reported on a mix of LAs and MATs. We report summary characteristics and quality grades for each grey literature study in Online Resource 10 and provide the full list of included references in Online Resource 11.

### Narrative summary of literature, by topic investigated and study quality

#### SEND identification/provision broadly (3 peer-reviewed & 50 grey literature studies)

This section includes studies examining the overall performance and functioning of the SEND system or across multiple aspects of SEND identification and provision at LA or MAT level. Examples include the financial expenditure on SEND services within an LA, or evaluations of LA or MAT-level activities and processes after the transition to the implementation of The Children and Families Act (CFA) 2014.

#### Peer-reviewed literature on SEND identification and provision

One high-quality study (Wood & Legg, 2020) explored practitioners’ vision for SEND provision in a single MAT. Identification and support for children with SEND was inequitable across the MAT, and some children were not fulfilling their potential due to inconsistent application of SEND identification systems and processes, even when various ‘diagnostic assessments’ were available. Good practice enablers were leaders with ‘clear direction and accountability for outcomes of learners with SEND’, ‘whole-trust, holistic practices’, pooling resources and expertise and sharing best practice. Barriers included ’under resourcing’, lack of curriculum ownership, ineffective tailoring of provision, external pressures (e.g. inspection visits from Ofsted), and the need to ‘illustrate strong pupil outcomes’ and be accountable for them. Another high-quality study (Roman-Urrestarazu et al., 2021), which used administrative data from England, identified significant differences in autism prevalence by sex (4.32 male:1 female), across racial/ethnic minority groups, among those with English as an additional language, and between LAs and geographic areas. The authors suggest these disparities could reflect differences in phenotypic prevalence and/or ‘differences in detection or referral’ for particular groups.

One low-quality study (Palikara et al., 2019) explored education professionals’ views on the 2014 SEND reforms at the LA level. While some participants said they could improve cross-service working, concerns were raised about the ‘gap between ideology and implementation’, particularly when no additional staff or funding was provided locally to manage the transfer of statements of SEN to EHCPs, with reports of staff burnout. Participants also reported procedural inconsistencies between LAs and resulting paperwork, out-of-date advice, poor interagency working, and low quality LOs. Participants wanted ‘clear guidelines and systematic, standardised training’ to improve implementation.

### Grey literature on SEND identification and provision

#### National government or DfE led or commissioned research (*n* = 4)

One medium-quality study commissioned by the DfE (Griggs, 2017) assessed 16 case studies of children with SEND who were part of an early education and development longitudinal study. The study concluded that funding cuts across certain LAs had led to increased inconsistency in LA-level provision. Selected LAs had sufficient resources and staffing to be heavily involved in the EHCP process, while others had limited resources and staffing, leading to low-quality EHCPs and delays in assessments.

The remaining three studies were low-quality but reflect the finding of inconsistent practice at LA level, often linked to inadequate funding and human resources. One found that only one-third of LAs provided additional funding to schools beyond the £6000 SEND funding allocation, and that criteria for allocating additional funding varied significantly across LAs (Parish, 2015). Another study suggested that the proactiveness of SENCos within LAs was a strong determinant of the quality of care provided to children with SEND (Department for Education, 2021). A third study highlighted that insufficient LA funding had reduced the quality of EHCPs, annual reviews, and SENCo-related care support. Heavy workloads had led to the loss of experienced staff, which in turn led to inconsistent quality of support within and between LAs (House of Commons Education Committee, 2019).

#### Inspectorate-led research (*n* = 5)

A medium-quality study (Ofsted, 2021b) used qualitative case studies to assess the quality of SEND provision in several schools across two LAs. SENCos reported that bureaucracy and delays within the LAs, related to referrals and EHCP assessments, were frustrating and reduced their effectiveness. The study also found that despite strategies and strong LA ‘ambitions for multi-agency collaboration’, these were not always realised in practice and did not always lead to improved outcomes. Schools and families reported long waiting times and excessive bureaucracy associated with the EHCP process, which resulted in CYP with SEND missing educational opportunities due to delayed or inaccurate identification of needs.

The remaining four studies were low-quality inspection reports assessing the implementation of the 2014 SEND reforms. They found that LAs consistently lacked joint commissioning and high quality, representative EHCPs. SENCos were an integral element in communicating information from schools to other agencies, however, lack of capacity led to limited communication that broadly reduced SEND care quality. Not all local areas had been inspected as should have happened according to the CFA 2014, and not all areas had been re-inspected due to inadequate provision (Ofsted & CQC, 2016; Ofsted & Care Quality Commission (Department for Education, 2021; Hayter et al. 2017; Ofsted, 2021a; Ofsted & CQC 2017). We only include one local area SEND inspection here, as there were more than 100 available at the time of our search. We have conducted a separate representative analysis of these individual LA inspections and re-inspections (Albajara Saenz 2025).

#### LA-led or commissioned research (*n* = 31)

All studies were low-quality, providing survey and interview data, and one ‘written statement of action’ following an inadequate rating of a local area inspection (Norfolk County Council, 2020). Overall, they highlight repeated inconsistencies in SEND provision across LAs. There were often delays in LAs creating EHCPs with most families reporting dissatisfaction with the assessment and EHCP process. A common reason for delays to EHCPs was the difficulty schools had with early identification of SEND (Bristol City Council, 2020), which was related to lack of specialist and general SEND training provided to mainstream schools for which LAs have a duty (Cambridgeshire County Council., 2016). Mainstream schools also have insufficient funding to meet the increased SEND demand (Scambler, 2021). Lack of communication between education, LAs, health and social care added to the delays in EHCP production. Work would typically be duplicated by different agencies which wasted valuable time and resources (Torbay Council, 2018). There was consistently poor communication throughout the entire system, including the communication towards families, between external agencies, and with SENCos (Portsmouth City Council, 2021; Swindon Borough Council, 2021). The lack of communication extended to families, where co-production was rare (Lancashire County Council, 2021). Families were often not included in decisions about their child’s care, and rarely attended meetings related to their child (Bournemouth Christchurch & Poole Council, 2021). The combination of late identification of SEND for some pupils and lack of multi-agency communication created delays in EHCPs and dissatisfaction within LAs (Vincent, 2021). EHCPs were typically low quality, and poorly written (Cheshire East Council, 2021). Lack of training, communication, and poorly written EHCPs were related back to insufficient funding relative to SEND demands (Deacon, 2017). The increased SEND-related workload with a comparatively small staffing base increased staff turnover, including the loss of experienced staff which has been a major point of dissatisfaction for families in some LAs (Cambridgeshire County Council., 2018).

#### Third sector-led research including national associations (*n* = 10)

All studies were low-quality, using interview and survey data to highlight the variability of SEND provision within their respective LAs. Most reports mentioned pervasive underfunding across the system. Firstly, there was a lack of funding in mainstream schools relative to their SEND demand (PACTS Stockport parent-carer forum, 2021). The number of requested EHCPs had risen by 35% in some areas yet funding and staffing appeared stagnant (Parish, 2018). Staff in mainstream education had been expected to support children with complex SEND despite limited time and minimal training by LAs, and 39% of families waited over 6 months from referral to EHCP production (British Association of Social Workers., 2018), though there was one report of low uptake of available training (Essex County Council., 2019). One study highlighted that 69% of home to school transport funding was spent on the SEND high needs block (Swords, 2019). Poor inter-agency communication between the LA and other SEND services was negatively affecting SEND referrals (Essex County Council., 2019). ECHPs reviews which are supposed to be annual, were also inconsistently held (Sheffield Parent Carer Forum, 2019). The lack of systemic coordination had made some families feel like they were “sent round in circles” as they are inappropriately passed between different SEND organisations (Healthwatch Swindon., 2020). Staff were often dismissive of claims from families, and communication from LAs fell short of what parents needed (Amaze, 2020).

### EHCPs (5 peer-reviewed & 9 grey literature studies)

#### Peer-reviewed studies of EHCPs processes and implementation

One high-quality scoping study about post-16 transitions (i.e. CYP over the age of 16 years) (Dunsmuir, 2020) noted that excessive bureaucracy of EHCPs was a challenge to their implementation. Two studies with SENCos soon after the 2014s reform - one medium (Sales & Vincent, 2018) and one high quality (Boesley & Crane, 2018) - identified what worked well, areas of concern, and how to support interagency working and family-led planning. Boesley et al’s (2018) participants said EHCPs had potential, but there were challenges around managing misconceptions and expectations from multiple parties about the SENCo role and EHCPs, lack of transparency and constant evolution of EHCP processes, decreased funding, difficulties accessing EHCPs for those with social, emotional and mental health (SEMH) needs due to over-emphasis of academic progress and difficulties validating SEMH needs. Sales and Vincent (2018) reported that EHCPs were not always needs-based, but were influenced by parental resources and advocacy ability, funding constraints and ‘high profile’ children. They highlighted continuing inequities and inconsistent application of legislation within and between LAs. Some children felt included in EHCP processes, but others were scared by formal meetings. Multi-agency working (health, education and social care) worked well if all professionals attended the meetings, but this was weakest for annual reviews.

Two small-scale low-quality studies echoed many of these challenges. One SENCo study (Richards, 2021) identified reduced LA funding and staff, insufficient information and training to implement the new system, and where enactment of EHCPs locally was influenced by ethos, extent of support, and quality of evidence. Palikara et al., (2019) cited concerns with poor LA-level compliance with the 20-week legal limit for EHCP processing, EHCP tendency to focus on education (not health and care), EHCP outsourcing and plans written by people with little SEND knowledge, and poor interagency working, all driven by the high demands of the new system, but with little training or guidance.

#### Grey literature studies of EHCPs processes and implementation

One medium-quality DfE commissioned survey of parents and young people (Adams, 2017) found that two-thirds were satisfied with overall experience of the EHC needs assessment and planning process, but there was variation at LA-level, and there was inconsistent consideration of families’ personal needs and circumstances. There was low use of independent advice and support services (IASS), the LO and personal budgets (5% of families had one, and less than one-fifth knew about them).

Most of the remaining studies (all low quality) focused on user satisfaction surveys with questions such as the extent people were involved in the process, if it was explained clearly, and if they had seen their EHCP and agreed with its contents. Many of these studies illustrate polarisation in reported experiences with a roughly even split of good versus poor (Bristol City Council, 2020; Cheshire East Council, 2021). Some surveys reported consistently positive experiences but were hampered by very small sample sizes or sample bias (e.g. only including those already accepted for EHCPs) (Devon County Council, 2018). Other low-quality research included secondary analysis of council data (Keer, 2016), identifying that 46/152 LAs had outsourced the writing of EHCPs to four firms over one year costing £1.6 million. The outsourcing process was opaque, but there was concern it would undermine EHCP co-production with children and families (especially as staff would not know the families), potentially contravened data protection laws, and with unknown impacts on EHCP quality. A government-level enquiry (House of Commons Women and Equalities Committee, 2020) into LA provision of EHCPs during the COVID-19 pandemic, found that relaxed expectations on LAs about their ‘reasonable endeavours’ duty continued for too long, leaving provision gaps for three months, with some LAs misinterpreting and failing to provide an ‘acceptable standard’, some ceasing all communication with children and families. The report further noted that the lack of ‘ring-fenced catch up funding’ for CYP with SEND would worsen ‘existing disparities in funding and outcomes’ compared to peers without SEND.

#### Peer-reviewed studies of EHCP quality

Three studies of EHCP quality all independently identified several similar concerns: namely wide variation (including at LA level) in the organisation of plans, extent of consultation of CYP and families, and the quality of written goals and outcomes. One high-quality study (Castro et al., 2019) assessed the quality of EHCP outcomes drawn from 11 London LAs and 46 schools, where most outcomes were rated as not functional and not high-quality. EHCP quality varied by the LA in which it was developed, school type, short vs long timeframe, and type of need, with the authors recommending standardised EHCP training and procedural guidelines. One medium-quality study of SENCos (Sales & Vincent, 2018) reported that EHCP outcomes and provision were not always ‘SMART’ (specific, measurable, achievable, realistic and time-specific) as specified in the SEND Code of Practice (DfE & DHSC, 2015), and would be strengthened by quantifying goals (e.g. number of provision hours/week), adapting the format to include a wider age group and those with communication difficulties, and involving and valuing parents more. One medium-quality study (Gaona et al., 2020) investigated the methods and extent that views, wishes and aspirations of autistic children were recorded in their EHCPs, and if plans were functional. The authors identified discrepancies in the organisation and content of EHCPs across the five LAs they compared, calling for specific guidelines to develop holistic, person-centred EHCPs.

#### Grey literature studies of EHCP quality

One medium-quality study commissioned by the DfE (Adams, 2017) also identified considerable LA-level variation in satisfaction with EHCPs. They investigated parent and child perceptions of EHCP quality, which were found to be positively appraised by parents but less well understood by children at younger ages. EHCPs for those aged 16–25 were more negatively appraised in terms of likelihood of future community participation, independent living, aspirations and employment.

A further small-scale low-quality interview study with parents/carers (Adams, 2018) identified the following factors leading to high EHCP satisfaction: the positive impact one proactive person from an LA or education setting can make, availability of specialists, EHCPs ready before a transition, families and professionals working together, plus face-to-face with meaningful involvement of CYP (not ‘box ticking’). Factors linked to low satisfaction were poor communication from LAs, inaccessible information and support, low transparency about delays and low recognition of their impact, and lack of involvement of families (e.g. in meetings), low attention to detail and lack of tailoring in EHCPs (copying and pasting, and generic plans), and lack of cooperation between schools and LAs. The study further involved SEND experts to assess the quality of 18 EHCPs (blinded to pre-existing satisfaction level) and found that satisfaction with the process was unrelated to EHCP quality, but varied by LA, age, previous ‘SEN statement’ (from the former SEND system, similar to an EHCP) and need type. EHCP domain quality scores ranged from 69%-91%, with better quality ratings if a consistent house style was used, with subheadings and first-person contributions from child and family members. In contrast, one low-quality council-led survey asked 18 parents/carers to report on the outcomes that had been enabled as a result of being issued with an EHCP (Lancashire County Council, 2021), with over two-thirds endorsing EHCPs, for example enabling better decision-making and more independence.

#### Complaints & redressal (3 peer-reviewed & 1 grey literature study)

Of the peer-reviewed literature, one high-quality qualitative study (Cullen, 2019) examined parent distress from SEND-related disputes. Distress was caused by significant unmet SEND needs, the demands over time, delays and unmet expectations when engaged with statutory processes, fear for the future and cumulative consequences on family life. Parents said LAs should listen and understand more, take responsibility and amend mistakes, invest in SEND services, and offer peer-support. A medium-quality study (Marsh, 2020) identified large LA-level variation in the percentage of EHCPs and SEND tribunal appeals relative to the child population from England-wide government data (2013-2018). LAs with lower EHCPs percentages had more appealable EHCP-related LA decisions (refusal to assess or write EHCPs, and about EHCP content) and had lower financing compared to LAs with higher EHCP percentages. This finding may be related to funding being partially based on historic spend in the national funding formula for schools and high needs.

One low-quality study (Lindsay, 2021) found lower appeal rates to the SEND tribunal for parents going through mediation with LAs, than those not using mediation, with associated average cost savings, but case complexity strongly influenced costs. There was also substantial between-LA variation in mediation service contacts and uptake.

One low-quality study from the grey literature (Bryant, 2022) gathered views of SEND professionals and LA staff about increasing appeals to the SEND tribunal. Increased service demand and reduced funding had led to poor provision and increased appeals. Disputes about EHCP content (including educational placements) were increasing, and fewer were resolved by mediation; lack of disaggregation of tribunal data prevented assessment of equity of access. Reducing disputes required good quality implementation of the CFA 2014 (e.g. joined-up pathways for families, via strong inter-agency decision-making). Challenges to reducing appeals were advocacy groups, contradictory professional reports, low clarity about SEND definitions and best practice guidelines e.g. what constituted ‘effective practice’ when LAs were balancing budgets and individual expectations, and ‘ordinarily available provision’.

#### Child voice & experience (2 peer-reviewed & 4 grey literature studies)

One small medium-quality study (Sharma, 2021) asked a range of LA SEND professionals and special school staff about barriers they faced when eliciting child voice in EHCPs. Professionals said CYP were often too young to meaningfully express their views, and resource constraints and differences in power dynamics with CYP undermined their ability to elicit children’s opinions. One low-quality study (Palikara, 2018) assessed depictions and processes used to elicit child voice in 184 EHCPs produced in nine Greater London LAs, in mainstream and special schools. LAs varied substantially in the processes and methods they used to capture CYP voices in EHCPs, and in the depiction of CYP voices (e.g. use of first person). In general, there was limited information included about CYP abilities and strengths.

The grey literature studies (all low quality) focused more on CYP educational access and experiences of educational settings and the wider SEND system, with LA-variation commonly identified, and mixed experiences amongst service-users. One study (Scott, 2016) identified how CYP support could become more ‘person-centred’ via changes to LA practices to improve transparency, communication, trust and understanding with families, other LAs and health services. This would require more training and resources and changed organisational culture to reduce local area variability. The study also noted a need for LAs to improve post-16 opportunities for CYP e.g. through brokering discussions and developing strategies with potential employers. Another study conducted during the COVID-19 pandemic (Care Quality Commission, 2020) demonstrated poorer access to education and the full curriculum by CYP if they had SEND compared to those who did not, partly due to administrative delays in arranging school transport and the issuing of part-time timetables (which is a joint LA-school responsibility). Short breaks provision also reduced, reducing CYP access to enrichment and support.

In contrast, one small LA-based survey of CYP with SEND (Bradford City Council, 2020) asked about health and wellbeing outcomes from their provision. Most respondents reported positively including the extent they thought their voice was heard, feeling supported, safe, valued, included, accepted, and able to learn. However, it was not clear how this information was attributable to local area SEND provision, and there was lack of clarity about how the findings would be incorporated into the LO and outcome frameworks as per the survey aims. Another LA study considered child experience of the SEND system as part of a review of the LO (Bristol City Council, 2020). Of 77 CYP respondents, 46% needed services they were not receiving (e.g. health, therapies, financial, leisure, equipment, assessments), awareness of Bristol’s LO was low (17%), but of those who had used it most found it helpful.

#### Practitioner roles & training (2 peer-reviewed & 1 grey literature study)

One high-quality study of SENCos (Curran, 2021) found their role to be highly variable. Some oversaw entire MATs; others worked at single school. They had differing levels of seniority and advocacy ability. They were overstretched, their time dominated by administrative tasks such as referrals to LAs, paperwork and meetings. They reported a lack of joint commissioning and joined up services, and lack of local consistency about time allocations for SENCo work, and standardisation and expectations about paperwork. One medium-quality study of teaching assistants (TAs) (Griffiths, 2018) found that dyslexia training was most effective when TAs were linked to LA advisory teams and they could receive ongoing mentoring support from LA specialist advisors during implementation, which many TAs feared would cease due to LA funding cuts.

One low-quality grey literature study from one LA surveyed SEND professionals (mostly working in education or early years) about their training needs (Swindon Borough Council, 2021). Respondents were confident about making positive relationships, communicating and working co-productively with CYP, families and other practitioners to meet needs using person-centred approaches, and to promote CYP strengths. They wanted information and training about personal budgets, preparation for adulthood from younger ages, complex case panels, participation, the LO, the legal framework around EHCPs, and the SEND and Inclusion Strategy.

#### Stakeholder strategy & planning (4 grey literature studies)

One medium-quality study focused on SEND strategy development (North Yorkshire County Council., 2021), generating key themes from focus groups with parents/carers and professionals. They agreed on constituents of a ‘good life’ for CYP with SEND (e.g. being valued, good mental health, wide ranging opportunities, independence). Obstacles for the strategy to address were strategic e.g. insufficient accountability and transparency and operational (e.g. understaffed workforce). Priorities for change included improving attitudes towards CYP and families; clearer, more accessible information, transparent communication, needs-led services, ending the postcode lottery, more social opportunities and respite. A low-quality study with CYP and families also aimed to develop a local SEND strategy (Council for Disabled Children., 2021). CYP valued existing activities and groups but wanted a wider choice and improved transport. Parents and CYP both wanted better preparation for employment in adulthood. They also wanted improved coordination of service inputs via a single key worker, and improved information sharing to minimise the need to repeat their story, and ensuring professionals had the most up-to-date information.

Two LAs (Westminster City Council, 2017; Wolverhampton Council, 2019) undertook Joint Strategic Needs Assessments, judged to be low-quality. The first sought to understand current service provision, gaps in services and areas of unmet need; the second adopted the lenses of independence and inclusion, and reducing ‘social and environmental barriers to CYP with SEND living ‘a good ordinary life’. In both studies CYP and families highlighted the need for continued engagement and cooperation between service providers, and challenges to early SEND identification and tailored support, (especially for autistic children in one study). Families also wanted clear, accessible information about available services, autism pathways and an improved (and autism-friendly) LO including a better range of leisure activities. Respondents from Westminster also called for enhancement of post-16 information, including pathways for specific SEND cohorts.

#### School exclusions (1 peer-reviewed & 1 grey literature study)

One medium-quality peer-reviewed study (Daniels, 2019) interviewed multiple stakeholders to understand how school exclusion practices (official and unofficial) affected CYP with SEND and their support. Key findings relevant to this review included, firstly, negative policy impacts. For example, the change from Independent Appeal to Independent Review Panels in parallel with the increasing academisation of schools ‘muddied’ the roles of academies and LAs for excluded children and made it more difficult for LAs to challenge schools about their exclusion practices, and where there was delayed reporting by some academies to LAs when CYP with SEND were identified. New guidance in the SEND Code of Practice also meant fewer CYP were recorded in SEN registers, which led some LAs to reduce specialist teacher provision. Secondly, the study identified increasing demand for SEND services (e.g. SEMH and ASD), which LAs had not yet adjusted to. Thirdly, the study found LA-level variability in policy and funding decisions, siloes within LAs which constrained multi-agency working, reduced staff which led to reactive working, and financial pressures including challenges with the ‘high needs block’ being used for excluded CYP, which reduced what LAs can use to support schools. There was also ad hoc allocation of provision by LAs per what was available geographically rather than being needs-led, and insufficient LA resources to transition CYP back into mainstream school.

One low-quality study (House of Commons Select Committee, 2018) considered the increasing rate of school exclusions of pupils from mainstream schools and referrals to alternative provision. Key findings relevant to LAs were that children who are excluded have no system of redress to challenge the decision and face an adversarial system with the school and LA, with no say over new placements. LAs themselves have low awareness of provision in their area for alternative placements, which leads to inappropriate and unscrutinised commissioning decisions and inappropriate placement decisions. LAs have little oversight or scrutiny over school decisions to exclude, or in checking unregistered providers.

#### Transition to adulthood (2 peer-reviewed & 2 grey literature studies)

One high-quality scoping study sought professional and manager perspectives on transitions for CYP aged 16–25 with SEND, which had undergone significant legislative and policy change since the CFA (Dunsmuir, 2020). Challenges voiced by participants included: lack of resources, ‘negotiating young people’s wishes’ and knowing when participants understood, navigating tension between child, parent and professionals about educational and living arrangements, excessive bureaucracy to create EHCPs, high demands of reconfiguring services, and ability of all required professionals to attend team meetings. Inter-professional teams were established through legislative change, rather than being self-selected, with evidence of ‘resistance to change in cultural norms and structures’. One low-quality study (Malkani, 2021) identified success factors for post-16 transitions in one LA including programmes that involve families, start earlier (by age 13-14), and where local employers are onboard, to improve employment chances and create mentoring opportunities. Clear interagency communication was required to fully understand CYP strengths, needs and capabilities, and to ensure good transitions to adult health services. Families called for additional chances for CYP to participate in settings beyond education to foster positive development, sense of belonging and purpose.

Both grey literature studies were low-quality. The first (Jones, 2018) examined funding and support for apprentices with SEND from the perspective of employers, third-party organisations and apprenticeship providers. LA involvement in recruitment for apprenticeships was viewed positively because it enabled targeting of disadvantaged groups. Providers were ‘more likely to be aware of the wider support needs of the apprentices' when LAs were involved in contrast to other recruitment programmes requiring self-declaration of additional needs. Challenges included EHCPs being required to access programmes, which were increasingly difficult to obtain due to LA resistance. Third-sector organisations reported funding was too ‘contract driven’ and in-flexible to enable adequate support. Other funding concerns included apprentices with undiagnosed additional support needs, including mental health, where employers were unclear about eligibility, required evidence, and restrictions on use of funds. One survey of parents who had children with SEND (Natspec, 2021) asked about the quality of information, advice and guidance LAs provided about post-16 options. Respondents wanted clearer impartial information (including through the LO) and planning to start earlier to enable smooth transitions. Parents thought LA information was edited by LAs to steer young people based on cost, wanted their preferences to be taken seriously, and for CYP to be more involved in decision-making.

#### Local offer for SEND (30 grey literature studies)

All LO for SEND studies were from the grey literature and graded as low-quality. Generally, the studies were led or commissioned by LAs, and sought feedback from service-users, with mixed experiences reported. Areas of investigation included: awareness of it, what people used it for, surveys/feedback about the website (useability and the quality of information it provided), and feedback about short breaks.

Awareness of the LO was mixed, as was being able to find required information. In several LAs, most respondents had heard of and were aware of the type of information the LO provided e.g. (Torbay Council, 2015). Parents often used the LO to find out about EHC needs assessments (Hodgson, 2021). In other LAs most respondents found the LO information useful but sometimes not up to date (Plymouth City Council, 2019) or information was missing (Reading Borough Council, 2022). Other LAs found more negative experiences, such as where most parents found the LO difficult to use (Doncaster Borough Council, 2017; South Gloucestershire Council, 2021). In some LAs none of the CYP surveyed had heard of the LO e.g. (Slough Borough Council, 2020).

Unsurprisingly, similarly mixed views were found about the LO website itself which likely to be the main medium through which LO information is accessed. Many service-users found the website useful with easy to find information in one LA and liked the website design (Child Friendly Leeds., 2021). In another LA, respondents rated the LO website highly and mostly found the LO information easily (Wakefield City Council, 2016). However, another online survey identified that most respondents were dissatisfied with the Nottinghamshire LO website (Smith, 2021). In other studies, none of the CYP asked had used the website before (St Helens Borough Council, 2017; Staffordshire County Council, 2022). Some LAs engaged service-users in LO website testing (Staffordshire County Council, 2019), accepting feedback from CYP about suggested changes to the website to make it more child friendly.

Studies investigating short-breaks provision also found mixed experiences, but they were more negative than positive. In some LAs, CYP reported enjoying short breaks provisions, and group activities out of school were the most popular (Wakefield City Council, 2018). In other LAs most families reported their short breaks provisions as safe, fun and helpful for fostering independence in adulthood (Herefordshire Council, 2018) and rated the short breaks positively (Oldham County Council, 2022). In contrasting LAs, many families faced issues finding the information they required on short breaks and said there was a lack of activities local to them (O’Malley, 2021). In one LA, more than 50% of CYP could not attend activities due to lack of support and felt unsatisfied with short breaks provisions (North Somerset Council, 2019). There were further questions about how accessible short breaks provision were in other LAs. For example, one where 70% of eligible families had not received short breaks provisions at all (MacDonald, 2021), and another where short breaks activities were described as inaccessible and only moderately met CYPs needs (Stevens, 2020).

### Summary of open-access data sources for SEND

We identified eight open-access data sources and data series which include SEND-related information at LA-level (see Table 3). These sources cover areas such as SEND prevalence by school type, transport, spending and complaints. We also found several online tools allowing SEND information at LA level to be combined in bespoke tables. Some sources offered disaggregation by SEND type, age and other sociodemographic characteristics, but most did not offer any disaggregation to explore equity of coverage. Trends show variable numbers of children with EHCPs, shortages of school placements in both mainstream and specialist settings, differing geographies of LAs (transport costs more in rural areas), and practical differences (such as start and finish times for post-16 provision). Complaints data reflect increased complaints over time, as well as increased SEND prevalence and EHCPs over time. Not all the sources we identified appear in the SEND summary of data sources document provided by the DfE, such as LGSCO complaints (DfE, 2024).

**Table 3:** Summary of eight open access data sources with SEND-related information at local authority or multi-academy trust levels in England.

Author (source)	Title	Date	Aspects of SEND included	Data type	LA/MAT coverage	Study design	Population involved	Publication aims	Socio-demographic disaggregation of data	Outcomes	Recent trends
**Complaints and redressal**											
LGSCO (Local Government and Social Care Ombudsman)	Special educational needs archive (2021-2022/ 2020-2021/ 2019-2020/ 2018-2019/ 2017-2018/ 2016-2017/ 2015-2016)	2015–2022	Complaints about councils/social care providers/ organisations providing local public services	Archive	All LAs	Archive (grouped by year)	Parents/carers submitting complaints on SEND-related issues	Publication on decisions in accordance with transparent decision-making processes	NA	Ombudsman decisions, upheld/not upheld outcomes, and identified service failings	Increasing number of complaints and decisions published annually
Ministry of Justice	Tribunal Statistics Quarterly: January to March (2022/2021/2020/2019); Tribunals and gender recognition certificates statistics quarterly (January to March 2018; July to September 2017/2016/2015)	2015–2022	Tribunal data	Official statistics	All LAs	Repeated cross-sectional, (quarterly, published annually in time series format)	Parents/carers and CYP with SEND appealing against LA decisions related to EHCPs	Present rate of appeal to the SEND Tribunal for prior calendar year	Not available at LA level	SEND Tribunal appeal rate	Increase in appeal rate over time
**SEND prevalence, demand and provision**											
Department for Education (DfE)	Education, Health and Care Plans: England (2020–2022); Statements of SEN and EHC Plans: England (2015–2019)	2015–2022	EHCPs, statements of SEN	National statistics release (based on statutory SEN2 data return)	All LAs	Repeated cross-sectional, published annually	Children and young people (CYP) with an EHCP or a statement of SEN	To provide data on CYP with an EHCP/statement of SEN	Age	Number of statements of SEN and EHCPs: total, new, requests and assessments, issued within time limits, mediations and tribunals, personal budgets, transfers from statements of SEN to EHCP, traineeships, apprenticeships, and supported internships	•Increase in total EHCPs •Increase in initial requests and new EHCPs •Increase in new plans issued within 20 weeks
DfE– EES (Explore Education Statistics)	Special educational needs in England (Academic Year 2021–22/2020-21/2019-20; January 2019/2018/ 2017/ 2016/ 2015)	2015–2022	Pupils with SEN, SEN provision in schools	National statistics release (based on School Census returns)	All LAs	Repeated cross-sectional, published annually	CYP with SEN attending state-funded, independent and general hospital schools	To provide pupil level data on CYP with SEN	Age, gender, ethnicity, first language, FSME	Number and percentage of pupils with SEN; types of need; placement by school type	•Increase in pupils with SEN •Autism Spectrum Disorder (ASD) most common need among those with EHCPs •Speech, Language and Communication Needs (SLCN) most common for SEN Support
DfE - EES	Planned LA and school expenditure (Financial years 2015–16 to 2021–22)	2015–2022 (financial years)	Planned expenditure	Official statistics release (Section 251 Budget Return)	All LAs	Repeated cross-sectional, published annually	Schools, education and children’s and young people’s services	To provide information on LAs’ financial spending intentions regarding schools, education and children’s services	NA	Planned expenditure	•Largest planned increases in high needs block spending •Increase in high needs top-up funding and SEN support service planned expenditure •Increase in planned expenditure on other high needs funding categories
**Open-source databases to combine SEND data sources in bespoke tables**											
DfE - EES	Create your own tables (Explore Education Statistics tool)	Live tool, updated regularly	Customisable access to datasets across themes, including social care, early years, finance and funding, further education (FE), higher education (HE), and school outcomes	Interactive data tool	All LAs (depending on dataset)	NA (interface for custom table generation using existing statistical releases)	NA (varies by dataset)	To provide an interface for accessing and combining data in different areas of interest	Age, gender, ethnicity, FSME, English as first language and others depending on dataset	Custom outputs based on selected variables and filters from official datasets	Varies by dataset — users can explore SEND-related trends in key areas such as social care, attainment, and funding
DfE - Register of schools and colleges in England	Get Information about Schools (GIAS)	2017	SEN provision, special classes, Section 41 in schools and colleges, special schools	Register	All LAs	Administrative database (updated continuously)	Schools, colleges, academy trusts and other educational establishments	Register replacing Edubase service, provides details on schools, children’s centres, academy trusts and sponsors, school federations	Gender, age range, religious character, religious ethos, diocese, urban/rural	Searchable list of establishments and their characteristics	List of establishments, can be filtered by Section 41 approval status, special classes availability, type of SEN provision, special school establishment
Local Government Association (LGA)	LG Inform Tool	Updated regularly (live tool)	Tribunals, school absences, exclusions, academic attainment, level of development, phonics screening checks, statements of SEN, EHCPs, NEET and participation, early years foundation stage profile results, outcomes for children looked after by LAs, Special Educational Needs statistics in England, education provision, planned LA and school expenditure on SEN, destinations of key stage 4 and 16 to 18 (KS5) students, school workforce, pupil characteristics, services, children in need and child protection, schools inspections and outcomes	Data, reports	All LAs	Dashboard reporting using administrative and official statistics	NA	To support local authorities and stakeholders with data-driven decisions, transparency, performance monitoring, and benchmarking in education and SEND	Age, gender, FSM	Descriptive outcomes and trends for education and SEND indicators	Data updated regularly to reflect new releases from government and other official sources

*ASD* autism spectrum disorder, *CYP* children and young people, *DfE* Department for Education, *EES* Explore Education Statistics, *EHCP* education, health and care plan, *FE* Further education, *HE* higher education, *FSME* free school meal eligibility, *LAs* local authorities, *LGA* Local Government Association, *LGSCO* Local Government & Social Care Ombudsman, *NEET* not in education, employment or training, *SEND* special educational needs and disability, *SEN2* survey collecting aggregate LA level information on CYP with statements of SEN or EHCPs, *SLCN* speech, language and communication needs.

### Key SEND services and processes contributing LA and MAT-level variation

Collectively, medium and high-quality studies found substantial LA-level variation in how the 2014 SEND legislation and policy was implemented in multiple domains (e.g. Dunsmuir, 2020; Griggs, 2017; Ofsted, 2021b). Firstly, funding for SEND provision varied by LA. Funding shortfalls meant the system could not be implemented properly or meet the needs of an increasing SEND population (especially for autism and SEMH) and often led to resource-led rather than needs-led decision-making (Daniels, 2019; Sales & Vincent, 2018). Specific aspects of SEND provision that were affected included EHCP development (Sharma, 2021), ‘transition to adulthood’ programmes (Dunsmuir, 2020) and retaining enough staff to reduce the ‘postcode lottery’ (North Yorkshire County Council., 2021). Demand could be higher still if all those who were entitled to services were aware of them and if LAs improved information sharing with service-users (Adams, 2017; North Yorkshire County Council., 2021).

Secondly, lack of standardisation of key documents were often blamed for inconsistent practices at LA-level, particularly around SEND identification and EHCP development. For example, autism prevalence varied significantly by LA, as did the percentage of EHCPs amongst the pupil population suggesting LA-level differences in identification criteria and processes (Marsh, 2020; Roman-Urrestarazu et al., 2021). Regarding EHCPs, there was a call for improved training and guidelines so professionals could accurately describe CYP needs, elicit CYP views, authentically co-produce plans, ensure plans included meaningful and measurable outcomes, and bring consistency to how plans were organised (Adams, 2017; Boesley & Crane, 2018; Castro et al., 2019; Dunsmuir, 2020; Gaona et al., 2020; Sales & Vincent, 2018; Sharma, 2021).

Thirdly, variation in local capacity to deal with the heavy bureaucracy of the system was affecting waiting times for SEND services, leading to delayed identification and reduced quality of provision (Ofsted, 2021b), including the administration of EHCPs (Dunsmuir, 2020). SENCos reported being overstretched, facing excessive paperwork, and difficulties with inter-agency working (Boesley & Crane, 2018; Curran, 2021). Strong interagency working was critical for the system to work well and was influenced by all the above factors. For example, teaching assistants appreciated the value of strong links with LAs (Griffiths, 2018), whilst others reported the negative impact of siloed working, poor interagency cooperation and resistance to changing cultural norms about SEND provision by key agencies (Daniels, 2019; Dunsmuir, 2020). Variation in LA-level SEND provision was evident from studies consulting service-users and families, who were distressed by delays, ongoing unmet needs, not being listened to by LA staff, and lack of accountability and transparency regarding statutory processes and decision-making (Cullen, 2019; North Yorkshire County Council., 2021). This variation in satisfaction was also reflected in variable tribunal appeals from administrative data sources (Marsh, 2020).

Less commonly investigated negative influences on provision were wider policy influences, such as an over emphasis on academic outcomes (Boesley & Crane, 2018), and delayed SEND identification and reduced school-level accountability for pupil exclusions since the introduction of MATs (Daniels, 2019). Though very few studies examined SEND provision in the context of MATs, what there is suggests it may be contributing to inequities and inconsistencies in SEND provision (Wood & Legg, 2020). Finally, we note that, with one exception, all LA-led research into the overall quality of their services and the Local Offer, was low quality. These studies, as well as open access data sources for SEND, also often lacked disaggregation by socio-demographic characteristics or SEND types. Open access data sources and series were rarely disaggregated or combined to create indicators that could be used, for example, to understand variation in spending on specific aspects of SEND services at LA-level to enable efficiency saving and facilitate commissioning decisions (e.g. around SEND transport or therapies).

## Discussion

### Major sources of LA level variation in SEND provision identified by our review

Evidence we compiled, published in the early years following the 2014 SEND reforms, suggests that LAs were under-resourced, insufficiently trained and ill-prepared to implement them. Years later, many LAs are still failing to fully adhere to CFA legislation and related policy, with considerable variation between LAs in the extent of adherence. In several studies of all qualities LA staff have described a disconnect between ‘ideology versus implementation’ suggesting that the vision for the reforms has not been applied in practice. The 2014 SEND reforms coincided with funding cuts, and a decade later, the system remains under financial strain. Real-terms reductions in SEND expenditure have not kept pace with the rising number of children identified with SEND and having EHCPs (Institute for Fiscal Studies, 2024; National Audit Office, 2024). The financial capacity of LAs to absorb these pressures also varies; a minority of LAs (though growing in number) have declared that they cannot meet their funding commitments for the following year (Sandford, 2024).

Inconsistent application of the limited guidelines provided to LAs has led to long-lasting detrimental effects on SEND provision and the children who need it. It has also placed excessive pressure on professionals such as SENCos. The experience and responsibilities of SENCos vary widely, contributing to within and between LA variation. Many SENCos are reportedly reaching breaking point. The mismatch between what families expect to receive based on statutory entitlements and what they are offered or receive has caused a breakdown in relationships between professionals and families. Variation between LAs in the use of legal redress mechanisms, such as tribunal appeals, may reflect variable quality of local provision and levels of user satisfaction.

There is high-quality evidence of probable inequitable service access to autism services by ethnic minority groups, and LA variation in the identification of autism (Roman-Urrestarazu et al., 2021). EHCP processes and the quality of plans are another major source of local variation. EHCPs vary in structure, style and content (Castro et al., 2019), with no consistent method being used to capture the child’s voice, and where many lack SMART goals (Sales & Vincent, 2018). Lack of joint commissioning and poor multi-agency collaboration contribute to variability in both EHCP quality and associated processes. Moreover, the ‘Health’ component of EHCPs remains underrepresented and insufficiently integrated.

Nearly all research into Local Offers for SEND was poor quality and suggested mixed experiences of using it, and from the one medium quality study available it appears that there is low CYP awareness of it in at least one LA (North Yorkshire County Council., 2021). Local services linked to LOs can be a lifeline for children with SEND and their families, offering crucial information to help them understand and navigate the complex system. Without high quality research into this aspect of SEND provision we cannot know whether LOs are being used to their full potential, or if service-users’ reviews and feedback are being actively incorporated and used to shape future local offers (DfE & DHSC, 2015) Our own research, conducted since the searches for this review were completed, illustrates considerable variation in the extent to which LAs comply with SEND Code of Practice requirements and equality legislation in their LOs (Matthews, et al., 2024).

### Major sources of MAT level variation in SEND provision identified by our review

Considering that nearly half of all schools are now part of MATs (Plaister, 2023), there is very little research at MAT level exploring their impact on SEND identification and provision. The research we found suggests MATs are likely contributing to local variability in both SEND identification and provision. This included evidence that the introduction of MATs has worsened the experiences of CYP with SEND and increased their risk of school exclusion, which is variable by LA. One study found variation in identification and provision even within a single MAT, despite the presence of good leaders and tools available to support identification (Wood & Legg, 2020). Overall whilst some professionals thought MATs had a theoretical potential to do good (Flemons, 2024) like LAs, their accountability mechanisms are weak, and LAs have little power to investigate poor practice at MAT-level, which aligns with research published since our review (Hutchinson, 2019, 2025).

### Implications of our findings

LAs need more funding and staff to clear the backlog that has built up over the years and is being compounded by real-term reductions in spending. Part of the long-term strategy of investment and strengthening the SEND system should include improving and monitoring Local Offers for SEND with robust evaluation mechanisms in place for this and other services. This could include more extra-curricular activities, greater short breaks provisions, and signposting the existence of the LO to newly identified CYP and families. Any further SEND reforms should include providing professionals with standardised reporting to increase consistency in processes and clarity for the families engaging with the system. In line with this, more work is needed to make EHCPs truly child-centred, including evidence-based methods to elicit the child’s voice. Similarly, LA activities around the preparation of CYP with SEND for adulthood should happen earlier, with more meaningful engagement and support to businesses so CYP have a better chance of employment. Currently, the extent of LA involvement and engagement with businesses also seems to vary.

### Using existing data sources to reduce local variation and improve accountability

Our research revealed both a frustration from service-users about lack of LA accountability for poor SEND provision, and a multitude of existing data sources that could be combined and leveraged to strengthen local monitoring and accountability. Aside from complaints and appeals data, there is little other monitoring data, particularly in real-time, to observe variation in LA performance and to hold them to account for provision; there is even less for MATs. The SEND improvement plan begun by the previous government (UK Government, 2023) included the development of data dashboards for this purpose, but it is not clear which data sources would be used, or who would decide which indicators should be included. We recommend that researchers and independent watchdog organisations could develop LA and MAT level dashboards, as well as the use of freedom of information requests as appropriate to plug information gaps with existing administrative, routinely collected data. A full understanding of freely available data sources with SEND data could help build a meaningful data dashboard for monitoring and evaluation of SEND services, to provide targets for quality improvement and to strengthen accountability with a mixture of carefully designed and evaluated incentive and reward strategies (National Audit Office, 2009). Other countries facing similar gaps in their understanding of SEN/D systems, or other complex public services, could consider harnessing administrative data for this purpose. For example, Gilmour & Stiefel (2025) describe a number of policy relevant evaluation questions and analysis methods to apply to administrative sources of SEN data from the USA which could equally be applied elsewhere.

In England, sustainable dashboards could also be developed from within the SEND system. An existing dashboard that we are aware of, LG-Inform tool (Local Government Association, 2025) reports on several indicators related to SEND, including for example, the percentage of EHCP applications that were processed within the 20-week statutory time limit, EHCPs, and mediation and tribunal appeals. Whilst important, other key areas such as LGSCO complaints data, and spending per child with SEND on different therapies and transport, are omitted. Neither does the tool disaggregate by sociodemographic information to be able to examine the extent of equitable access to SEND services and CYP outcomes; this should be possible for some characteristics provided they are not so rare as to identify individual service-users. A further government source reports on SEND-related data sources (DfE, 2024), but it is not comprehensive (for example it excludes SEND transport data), and it does not assimilate the sources or report on trends over time. Only a minority of the sources it includes offer disaggregated statistics. Disaggregated data allow consideration of equity in provision and service access, can improve planning, budgeting, monitoring and evaluation, ensuring that ‘no-one is left behind’ (Abualghaib et al., 2019); this principle applies beyond the SEND system in England to other countries and systems, aligning with the ‘central transformative promise’ of UN Member States working towards the 2030 Sustainable Development Goals (United Nations, 2025). Within and outside of this list are data sources which if combined hold strong potential to reduce local variation and drive-up quality of provision.

### Remaining evidence gaps and research priorities

Our review identified several evidence gaps and research priorities. Firstly, very few studies examined equity and inequality of SEND service provision locally and few data sources disaggregate by socio-demographic characteristics associated with inequality. Secondly, there are very few studies about the impact of MATs on SEND identification and provision, and on how to minimise local variation. Thirdly, there are few studies focused on transition planning for CYP post-16. Fourthly, most of the research we identified was low quality, particularly whole service evaluations and Local Offer studies by LAs. Better quality research is needed, for example on parent/carer satisfaction, to avoid misleading interpretations and the use of low-quality findings to plan services. Rigour could be improved with stronger study designs, such as time trend analyses to evaluate services, and pilot testing or staggered implementation of new strategies in some LAs with counterfactuals or exploiting natural experiments to evaluate LA practices against child and family outcomes. There is a wealth of routinely collected administrative data to facilitate this, saving time and resources otherwise spent on primary data collection, is valuable in being population-representative, and facilitates the study of vulnerable or hard-to-reach populations, such as CYP with SEND and their families (Cole., 2022; Ellard-Gray et al., 2015; Milne et al., 2022).

### Limitations

Outcomes for CYP with SEND and their families were out of scope of this review but are a critical marker of service quality and equity of access. Similarly, school level variability was out of scope, and will have large influence on SEND provision and outcomes, particularly children who have lower level ‘SEN support’ which is largely overseen by schools, and who constitute the majority of SEND learners. Our review also identified within LA-variation, which is person-dependent, and emphasises the importance of strong SEND leadership at LA and MAT levels to set a clear tone and expectations for provision. For practical reasons we limited our screening process to the first ten pages of results from local authority website searches, and it is possible we missed relevant sources that were not picked up via our other search strategies.

## Conclusion

There are multiple causes of local variation in SEND provision from inconsistent implementation of CFA legislation. Variation is unfair for service-users and could increase as most schools are assimilated into MATs. Greater standardisation, increased resources and better use of existing data to strengthen monitoring, evaluation and accountability of local bodies could reduce variation in provision, improve quality and increase equity of access to services.

## Supplementary information

Supplementary information-Anonymised_v2

## Data Availability

All data generated or analysed during this study are included in this published article and its supplementary information files, except for domain-specific quality grades for each included study—these are available from the authors on reasonable request.
