# Supplementary material for: Examining local level variation in Special Educational Needs and Disabilities (SEND) service provision and associated data sources in England: a scoping review
Source: Humanit Soc Sci Commun. 2026 Feb 10;13(1):306. doi: 10.1057/s41599-025-06319-0 (PMC12999517; doi:10.1057/s41599-025-06319-0)
Supplement: Supplementary file 1 — Supplementary information-Anonymised_v2 [file 41599_2025_6319_MOESM1_ESM.docx]

# Supplementary material

# **Examining local level variation in Special Educational Needs and Disabilities (SEND) service provision and associated data sources in England: A scoping review**

## Online resource 1. List of local authorities included in the review

| **No.** | **Local Authority** | **Region** |
| --- | --- | --- |
| 1 | Barking and Dagenham | London |
| 2 | Barnet | London |
| 3 | Barnsley Borough Council | Yorkshire and The Humber |
| 4 | Bath and North East Somerset Council | South West |
| 5 | Bedford Borough Council | East of England |
| 6 | Bexley | London |
| 7 | Birmingham City Council | West Midlands |
| 8 | Blackburn with Darwen Borough Council | North West |
| 9 | Blackpool Council | North West |
| 10 | Bolton Borough Council | North West |
| 11 | Bournemouth, Christchurch and Poole Council | South West |
| 12 | Bracknell Forest Borough Council | South East |
| 13 | Bradford City Council | Yorkshire and The Humber |
| 14 | Brent | London |
| 15 | Brighton and Hove City Council | South East |
| 16 | Bristol City Council | South West |
| 17 | Bromley | London |
| 18 | Bury Borough Council | North West |
| 19 | Buckinghamshire County Council | South East |
| 20 | Calderdale Borough Council | Yorkshire and The Humber |
| 21 | Cambridgeshire County Council | East Of England |
| 22 | Camden | London |
| 23 | Central Bedfordshire Council | East of England |
| 24 | Cheshire East Council | North West |
| 25 | Cheshire West and Chester Council | North West |
| 26 | City of London | London |
| 27 | City of York Council | Yorkshire and The Humber |
| 28 | Cornwall Council | South West |
| 29 | Coventry City Council | West Midlands |
| 30 | Croydon | London |
| 31 | Cumbria County Council | North West |
| 32 | Darlington Borough Council | North East |
| 33 | Derby City Council | East Midlands |
| 34 | Derbyshire County Council | East Midlands |
| 35 | Devon County Council | South West |
| 36 | Doncaster Borough Council | Yorkshire and The Humber |
| 37 | Dorset Council | South West |
| 38 | Dudley Borough Council | West Midlands |
| 39 | Durham County Council | North East |
| 40 | Ealing | London |
| 41 | East Riding of Yorkshire Council | Yorkshire and The Humber |
| 42 | East Sussex County Council | South East |
| 43 | Enfield | London |
| 44 | Essex County Council | East Of England |
| 45 | Gateshead Borough Council | North East |
| 46 | Gloucestershire County Council | South West |
| 47 | Greenwich | London |
| 48 | Hackney | London |
| 49 | Halton Borough Council | North West |
| 50 | Hammersmith and Fulham | London |
| 51 | Hampshire County Council | South East |
| 52 | Haringey | London |
| 53 | Harrow | London |
| 54 | Hartlepool Borough Council | North East |
| 55 | Havering | London |
| 56 | Herefordshire Council | West Midlands |
| 57 | Hertfordshire County Council | East Of England |
| 58 | Hillingdon | London |
| 59 | Hounslow | London |
| 60 | Isle of Wight Council | South East |
| 61 | Isles of Scily | South West |
| 62 | Islington | London |
| 63 | Kensington and Chelsea | London |
| 64 | Kent County Council | South East |
| 65 | Kingston Hull City Council | Yorkshire and The Humber |
| 66 | Kingston upon Thames | London |
| 67 | Kirklees Borough Council | Yorkshire and The Humber |
| 68 | Knowsley Borough Council | North West |
| 69 | Lambeth | London |
| 70 | Lancashire County Council | North West |
| 71 | Leeds City Council | Yorkshire and The Humber |
| 72 | Leicester City Council | East Midlands |
| 73 | Leicestershire County Council | East Midlands |
| 74 | Lewisham | London |
| 75 | Lincolnshire County Council | East Midlands |
| 76 | Liverpool City Council | North West |
| 77 | Luton Borough Council | East of England |
| 78 | Manchester City Council | North West |
| 79 | Medway Council | South East |
| 80 | Merton | London |
| 81 | Middlesbrough Borough Council | North East |
| 82 | Milton Keynes Council | South East |
| 83 | Newcastle Upon Tyne City Council | North East |
| 84 | Newham | London |
| 85 | Norfolk County Council | East Of England |
| 86 | North East Lincolnshire Council | Yorkshire and The Humber |
| 87 | North Lincolnshire Council | Yorkshire and The Humber |
| 88 | North Somerset Council | South West |
| 89 | North Tyneside Borough Council | North East |
| 90 | North Yorkshire County Council | Yorkshire and the Humber |
| 91 | Northamptonshire County Council | East Midlands |
| 92 | Northumberland County Council | North East |
| 93 | Nottingham City Council | East Midlands |
| 94 | Nottinghamshire County Council | East Midlands |
| 95 | Oldham Borough Council | North West |
| 96 | Oxfordshire County Council | South East |
| 97 | Peterborough City Council | East of England |
| 98 | Plymouth City Council | South West |
| 99 | Portsmouth City Council | South East |
| 100 | Reading Borough Council | South East |
| 101 | Redbridge | London |
| 102 | Redcar and Cleveland Borough Council | North East |
| 103 | Richmond upon Thames | London |
| 104 | Rochdale Borough Council | North West |
| 105 | Rotherham Borough Council | Yorkshire and The Humber |
| 106 | Rutland County Council | East Midlands |
| 107 | Salford City Council | North West |
| 108 | Sandwell Borough Council | West Midlands |
| 109 | Sefton Borough Council | North West |
| 110 | Sheffield City Council | Yorkshire and The Humber |
| 111 | Shropshire Council | West Midlands |
| 112 | Slough Borough Council | South East |
| 113 | Solihull Borough Council | West Midlands |
| 114 | Somerset County Council | South West |
| 115 | South Gloucestershire Council | South West |
| 116 | South Tyneside Borough Council | North East |
| 117 | Southampton City Council | South East |
| 118 | Southend-on-Sea Borough Council | East of England |
| 119 | Southwark | London |
| 120 | St Helens Borough Council | North West |
| 121 | Staffordshire County Council | West Midlands |
| 122 | Stockport Borough Council | North West |
| 123 | Stockton-on-Tees Borough Council | North East |
| 124 | Stoke-on-Trent City Council | West Midlands |
| 125 | Suffolk County Council | East of England |
| 126 | Sunderland City Council | North East |
| 127 | Surrey County Council | South East |
| 128 | Sutton | London |
| 129 | Swindon Borough Council | South West |
| 130 | Tameside Borough Council | North West |
| 131 | Telford and Wrekin Borough Council | West Midlands |
| 132 | Thurrock Council | East of England |
| 133 | Torbay Council | South West |
| 134 | Tower Hamlets | London |
| 135 | Trafford Borough Council | North West |
| 136 | Wakefield City Council | Yorkshire and The Humber |
| 137 | Walsall Borough Council | West Midlands |
| 138 | Waltham Forest | London |
| 139 | Wandsworth | London |
| 140 | Warrington Borough Council | North West |
| 141 | Warwickshire County Council | West Midlands |
| 142 | West Berkshire Council | South East |
| 143 | West Sussex County Council | South East |
| 144 | Westminster | London |
| 145 | Wigan Borough Council | North West |
| 146 | Wiltshire Council | South West |
| 147 | Windsor and Maidenhead Borough Council | South East |
| 148 | Wirral Borough Council | North West |
| 149 | Wokingham Borough Council | South East |
| 150 | Wolverhampton City Council | West Midlands |
| 151 | Worcestershire County Council | West Midlands |

## Online resource 2. Search terms for peer-reviewed and grey literature sources

| “Local authorit*” OR “Local area” OR District OR Council OR “Academ*” |
| --- |
| AND |
| England OR [Greater London](https://eur03.safelinks.protection.outlook.com/?url=https%3A%2F%2Fsimple.wikipedia.org%2Fwiki%2FGreater_London&data=05%7C02%7Cjcs230%40medschl.cam.ac.uk%7C13eb4389aff7487ed52408dcab154649%7C49a50445bdfa4b79ade3547b4f3986e9%7C1%7C0%7C638573355060954377%7CUnknown%7CTWFpbGZsb3d8eyJWIjoiMC4wLjAwMDAiLCJQIjoiV2luMzIiLCJBTiI6Ik1haWwiLCJXVCI6Mn0%3D%7C0%7C%7C%7C&sdata=7q6OChZ5OSgl6OWRlkOcZij6BvBDTfQnwLLDxtpLBiM%3D&reserved=0) OR [South East](https://eur03.safelinks.protection.outlook.com/?url=https%3A%2F%2Fsimple.wikipedia.org%2Fwiki%2FSouth_East_England&data=05%7C02%7Cjcs230%40medschl.cam.ac.uk%7C13eb4389aff7487ed52408dcab154649%7C49a50445bdfa4b79ade3547b4f3986e9%7C1%7C0%7C638573355060965218%7CUnknown%7CTWFpbGZsb3d8eyJWIjoiMC4wLjAwMDAiLCJQIjoiV2luMzIiLCJBTiI6Ik1haWwiLCJXVCI6Mn0%3D%7C0%7C%7C%7C&sdata=bvjWcyUQLjbeP6HhCTkcP7AP4XGrMZ6Tt1UOQhenf10%3D&reserved=0) England OR [South West](https://eur03.safelinks.protection.outlook.com/?url=https%3A%2F%2Fsimple.wikipedia.org%2Fwiki%2FSouth_West_England&data=05%7C02%7Cjcs230%40medschl.cam.ac.uk%7C13eb4389aff7487ed52408dcab154649%7C49a50445bdfa4b79ade3547b4f3986e9%7C1%7C0%7C638573355060973015%7CUnknown%7CTWFpbGZsb3d8eyJWIjoiMC4wLjAwMDAiLCJQIjoiV2luMzIiLCJBTiI6Ik1haWwiLCJXVCI6Mn0%3D%7C0%7C%7C%7C&sdata=96HGLXdsApMDt3d86gYyMADMEwPBu7jo768adSKT1Wg%3D&reserved=0) England OR [West Midlands](https://eur03.safelinks.protection.outlook.com/?url=https%3A%2F%2Fsimple.wikipedia.org%2Fwiki%2FWest_Midlands_(region)&data=05%7C02%7Cjcs230%40medschl.cam.ac.uk%7C13eb4389aff7487ed52408dcab154649%7C49a50445bdfa4b79ade3547b4f3986e9%7C1%7C0%7C638573355060981351%7CUnknown%7CTWFpbGZsb3d8eyJWIjoiMC4wLjAwMDAiLCJQIjoiV2luMzIiLCJBTiI6Ik1haWwiLCJXVCI6Mn0%3D%7C0%7C%7C%7C&sdata=D5a7r5HtzNuq4%2BogbVT7yyMgYjOxgR5mhajgwc7DIPc%3D&reserved=0) OR [North West](https://eur03.safelinks.protection.outlook.com/?url=https%3A%2F%2Fsimple.wikipedia.org%2Fwiki%2FNorth_West_England&data=05%7C02%7Cjcs230%40medschl.cam.ac.uk%7C13eb4389aff7487ed52408dcab154649%7C49a50445bdfa4b79ade3547b4f3986e9%7C1%7C0%7C638573355060991156%7CUnknown%7CTWFpbGZsb3d8eyJWIjoiMC4wLjAwMDAiLCJQIjoiV2luMzIiLCJBTiI6Ik1haWwiLCJXVCI6Mn0%3D%7C0%7C%7C%7C&sdata=qp%2FqZQ0gLKD2S5J5yRVQJ0M5ewKue%2BsLeoz4tF7hreA%3D&reserved=0) England OR [North East](https://eur03.safelinks.protection.outlook.com/?url=https%3A%2F%2Fsimple.wikipedia.org%2Fwiki%2FNorth_East_England&data=05%7C02%7Cjcs230%40medschl.cam.ac.uk%7C13eb4389aff7487ed52408dcab154649%7C49a50445bdfa4b79ade3547b4f3986e9%7C1%7C0%7C638573355061000434%7CUnknown%7CTWFpbGZsb3d8eyJWIjoiMC4wLjAwMDAiLCJQIjoiV2luMzIiLCJBTiI6Ik1haWwiLCJXVCI6Mn0%3D%7C0%7C%7C%7C&sdata=L9hXpXyrzaguNNMRO0JEMnLbZr4qA7xOUiEfvyXg8no%3D&reserved=0) England OR [Yorkshire and the Humber](https://eur03.safelinks.protection.outlook.com/?url=https%3A%2F%2Fsimple.wikipedia.org%2Fwiki%2FYorkshire_and_the_Humber&data=05%7C02%7Cjcs230%40medschl.cam.ac.uk%7C13eb4389aff7487ed52408dcab154649%7C49a50445bdfa4b79ade3547b4f3986e9%7C1%7C0%7C638573355061008298%7CUnknown%7CTWFpbGZsb3d8eyJWIjoiMC4wLjAwMDAiLCJQIjoiV2luMzIiLCJBTiI6Ik1haWwiLCJXVCI6Mn0%3D%7C0%7C%7C%7C&sdata=%2FV73EO4j1hT6rR%2FvjvziVmN4JmlWW2XVctM1OF0VkUs%3D&reserved=0) OR [East Midlands](https://eur03.safelinks.protection.outlook.com/?url=https%3A%2F%2Fsimple.wikipedia.org%2Fwiki%2FEast_Midlands&data=05%7C02%7Cjcs230%40medschl.cam.ac.uk%7C13eb4389aff7487ed52408dcab154649%7C49a50445bdfa4b79ade3547b4f3986e9%7C1%7C0%7C638573355061016258%7CUnknown%7CTWFpbGZsb3d8eyJWIjoiMC4wLjAwMDAiLCJQIjoiV2luMzIiLCJBTiI6Ik1haWwiLCJXVCI6Mn0%3D%7C0%7C%7C%7C&sdata=C1OfzjOlzsDG1rnQYDBZISo%2BW1P1Nxw%2BpskRy09AQqM%3D&reserved=0) OR [East of England](https://eur03.safelinks.protection.outlook.com/?url=https%3A%2F%2Fsimple.wikipedia.org%2Fwiki%2FEast_of_England&data=05%7C02%7Cjcs230%40medschl.cam.ac.uk%7C13eb4389aff7487ed52408dcab154649%7C49a50445bdfa4b79ade3547b4f3986e9%7C1%7C0%7C638573355061022287%7CUnknown%7CTWFpbGZsb3d8eyJWIjoiMC4wLjAwMDAiLCJQIjoiV2luMzIiLCJBTiI6Ik1haWwiLCJXVCI6Mn0%3D%7C0%7C%7C%7C&sdata=QZ5KOjGURiU%2B3yiUkV9%2Bishi3n6o4mMZftQQDuMSfEs%3D&reserved=0) |
| AND |
| “Special Educational Needs and Disability” OR “Special Educational Needs” OR SEND OR SEN OR “Speech, language and communication needs” OR ASD OR “Asperger’s Syndrome” OR Autism OR “moderate learning difficulties” OR “severe learning difficulties” OR “profound and multiple learning difficulties” OR “physical disability” OR “sensory impairment” OR “vision impairment” OR “hearing impairment” OR “multi-sensory impairment” OR “specific learning difficulties” OR dyslexia OR dyspraxia OR dyscalculia OR “challenging behaviour” OR “disruptive behaviour” OR “disturbing behaviour” OR “mental health difficulties” OR anxiety OR depression OR self-harming OR “substance misuse” OR “eating disorders” OR psychosomatic OR “functional disorder” OR “attention deficit disorder” OR “attention deficit hyperactive disorder” OR “attachment disorder” |
| AND |
| Provision OR intervention OR Identification OR service OR assessment OR support OR help OR local offer OR EHC OR ECHP OR Statement OR apprenticeships OR traineeships OR internships OR prevalence OR incidence OR cases OR variation OR review OR Tribunal OR budget OR “value for money” OR spend OR satisfaction OR feedback OR stakeholder OR collaboration OR co-production OR “joint commissioning” or mediation OR appeals OR complaints OR “Postcode Lottery” OR “human resources” OR vacancies OR staff* OR CCG |
| AND |
| “Children and Young People” OR “0-25 years” OR early years OR “post-16” OR “Adolescen*” OR “Young*” OR “Youth*” OR “Secondary School” OR “Primary School” OR School OR Education |

## Online resource 3. Search strategy for open access data sources and databases

| **Source** | **Search terms or hand-searched** |
| --- | --- |
| Official UK government website (https://www.gov.uk/) | “sen2”, “school capacity”, “section 251” |
| Explore Education Statistics service UK government website (https://explore-education-statistics.service.gov.uk/find-statistics) | “special educational needs”, “school expenditure” |
| Publications site Local Government Association website (https://www.local.gov.uk/publications) | “special educational needs” “spending trends SEND” |
| Local Government and Social Care Ombudsman website (https://www.lgo.org.uk/) | “special educational needs” |
| Family Hubs Network website (https://familyhubsnetwork.com/) | “special educational needs” |
| official website of the Association of Directors of Children’s Services Ltd (ADCS) website (https://adcs.org.uk/) | “school transport” |
| Tribunals statistics UK government website (https://www.gov.uk/government/collections/tribunals-statistics) | Hand-searched tribunal data related to decisions regarding SEN |
| Register of schools and colleges in England, UK government website (https://www.gov.uk/guidance/get-information-about-schools) | Hand-searched |

## Online resource 4: PRISMA diagram to illustrate flow of literature through the review


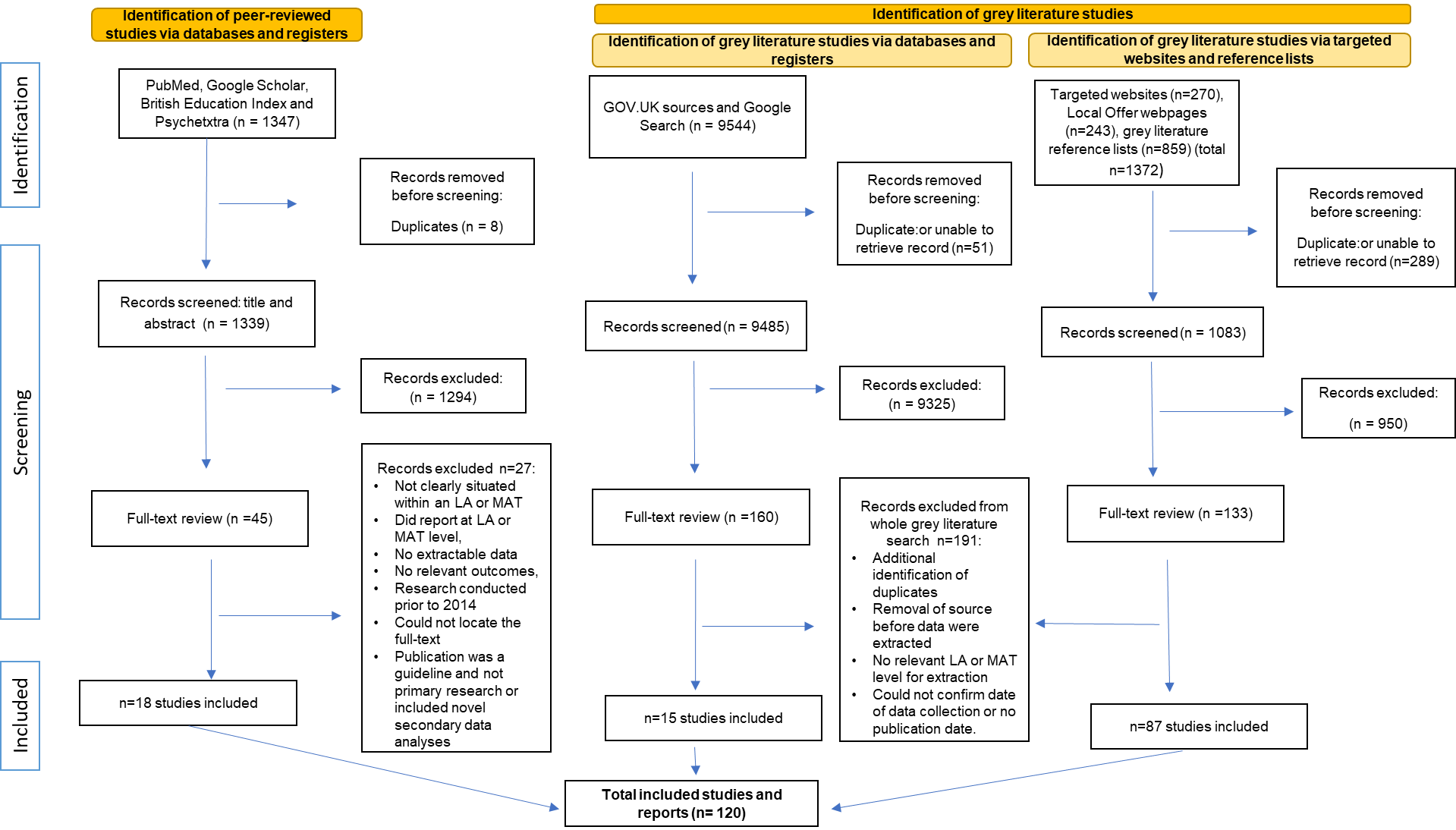


## Online resource 5. Data extraction table headings

1. Author name or organisation name,
2. Document title,
3. Grey source or peer-reviewed,
4. Year published,
5. Year of data collection,
6. Study Population,
7. Aspect(s) of SEND provision included
8. Type of environment/setting,
9. Local Authority or Multi-Academy Trust
10. Study/report design,
11. Intended audience,
12. Goal/objectives of document,
13. Any models/frameworks/guidance mentioned,
14. Key findings,
15. Was this a standalone report/study,
16. Was this a follow up to a previous report/study,
17. Was this part of a series of reports/studies,
18. Limitations (Author Identified),
19. Limitations (Reviewer Identified),
20. Recommendations from Author.

## Online resource 6. AACODS checklist for assessing the quality of grey literature

Jess Tyndall, Flinders University, Nov 2010. Archived at the Flinders Academic Commons:

http://dspace.flinders.edu.au/dspace/

| AACODS domain |  | Yes | No | ? |
| --- | --- | --- | --- | --- |
| Authority | **Identifying who is responsible for the intellectual content.**  *Individual author:*  • Associated with a reputable organisation?  • Professional qualifications or considerable experience?  • Produced/published other work (grey/black) in the field?  • Recognised expert, identified in other sources?  • Cited by others? (use Google Scholar as a quick check)  • Higher degree student under “expert” supervision?  *Organisation or group:*  • Is the organisation reputable? (e.g. W.H.O)  • Is the organisation an authority in the field?  In all cases:  • Does the item have a detailed reference list or bibliography? |  |  |  |
| Accuracy | • Does the item have a clearly stated aim or brief?  • Is so, is this met?  • Does it have a stated methodology?  • If so, is it adhered to?  • Has it been peer-reviewed?  • Has it been edited by a reputable authority?  • Supported by authoritative, documented references or credible sources?  • Is it representative of work in the field?  • If No, is it a valid counterbalance?  • Is any data collection explicit and appropriate for the research?  • If item is secondary material (e.g. a policy brief of a technical report) refer to the original. Is it an accurate, unbiased interpretation or analysis? |  |  |  |
| Coverage | All items have parameters which define their content coverage. These limits might mean that a work refers to a particular population group, or that it excluded certain types of publication. A report could be designed to answer a  particular question, or be based on statistics from a particular survey.  • Are any limits clearly stated? |  |  |  |
| Objectivity | It is important to identify bias, particularly if it is unstated or unacknowledged.  • Opinion, expert or otherwise, is still opinion: is the author’s standpoint clear?  • Does the work seem to be balanced in presentation? |  |  |  |
| Date | For the item to inform your research, it needs to have a date that confirms relevance  • Does the item have a clearly stated date related to content? No easily discernible date is a strong concern.  • If no date is given, but can be closely ascertained, is there a valid reason for its absence?  • Check the bibliography: have key contemporary material been included? |  |  |  |
| Significance | This is a value judgment of the item, in the context of the relevant research area  • Is the item meaningful? (this incorporates feasibility, utility and relevance)  • Does it add context?  • Does it enrich or add something unique to the research?  • Does it strengthen or refute a current position?  • Would the research area be lesser without it?  • Is it integral, representative, typical?  • Does it have impact? (in the sense of influencing the work or behaviour of others) |  |  |  |

## Online resource 7. MMAT Tool for grading peer-reviewed literature

From: Hong QN, Pluye P, Fàbregues S, Bartlett G, Boardman F, Cargo M, Dagenais P, Gagnon M-P, Griffiths F, Nicolau B, O’Cathain A, Rousseau M-C, Vedel I. Mixed Methods Appraisal Tool (MMAT), version 2018. Registration of Copyright (#1148552), Canadian Intellectual Property Office, Industry Canada.

| **Category of study designs** | **Methodological quality criteria** |  | | **Responses** | |
| --- | --- | --- | --- | --- | --- |
|  |  | Yes | No | Can’t tell | Comments |
| Screening questions (for all types) | S1. Are there clear research questions? |  |  |  |  |
|  | S2. Do the collected data allow to address the research questions? |  |  |  |  |
|  | *Further appraisal may not be feasible or appropriate when the answer is ‘No’ or ‘Can’t tell’ to one or both screening questions.* | | |  | |
| 1. Qualitative | 1.1. Is the qualitative approach appropriate to answer the research question? |  |  |  |  |
|  | 1.2. Are the qualitative data collection methods adequate to address the research question? |  |  |  |  |
|  | 1.3. Are the findings adequately derived from the data? |  |  |  |  |
|  | 1.4. Is the interpretation of results sufficiently substantiated by data? |  |  |  |  |
|  | 1.5. Is there coherence between qualitative data sources, collection, analysis and interpretation? |  |  |  |  |
| 2. Quantitative randomized controlled  trials | 2.1. Is randomization appropriately performed? |  |  |  |  |
|  | 2.2. Are the groups comparable at baseline? |  |  |  |  |
|  | 2.3. Are there complete outcome data? |  |  |  |  |
|  | 2.4. Are outcome assessors blinded to the intervention provided? |  |  |  |  |
|  | 2.5 Did the participants adhere to the assigned intervention? |  |  |  |  |
| 3. Quantitative nonrandomized | 3.1. Are the participants representative of the target population? |  |  |  |  |
|  | 3.2. Are measurements appropriate regarding both the outcome and intervention (or exposure)? |  |  |  |  |
|  | 3.3. Are there complete outcome data? |  |  |  |  |
|  | 3.4. Are the confounders accounted for in the design and analysis? |  |  |  |  |
|  | 3.5. During the study period, is the intervention administered (or exposure occurred) as intended? |  |  |  |  |
| 4. Quantitative descriptive | 4.1. Is the sampling strategy relevant to address the research question? |  |  |  |  |
|  | 4.2. Is the sample representative of the target population? |  |  |  |  |
|  | 4.3. Are the measurements appropriate? |  |  |  |  |
|  | 4.4. Is the risk of nonresponse bias low? |  |  |  |  |
|  | 4.5. Is the statistical analysis appropriate to answer the research question? |  |  |  |  |
| 5. Mixed methods | 5.1. Is there an adequate rationale for using a mixed methods design to address the research question? |  |  |  |  |
|  | 5.2. Are the different components of the study effectively integrated to answer the research question? |  |  |  |  |
|  | 5.3. Are the outputs of the integration of qualitative and quantitative components adequately interpreted? |  |  |  |  |
|  | 5.4. Are divergences and inconsistencies between quantitative and qualitative results adequately addressed? |  |  |  |  |
|  | 5.5. Do the different components of the study adhere to the quality criteria of each tradition of the methods involved? |  |  |  |  |

## Online resource 8. Peer-reviewed study characteristics and Mixed Methods Appraisal Tool (MMAT) grades, by area of investigation (n=18 studies)

| **First author & date** | **Study type** | **Respondents** | **Study design** | **Includes: LA, MAT, Both** | **Sample size & respondents** | **Study aim(s) or research questions** | **Methods** | **Main LA or MAT related findings** | **Overall study grade** |
| --- | --- | --- | --- | --- | --- | --- | --- | --- | --- |
| **Child voice & experience** | | | | | | | | | |
| Sharma, 2021 | At least two methods | Mixed stakeholders | Observational-Descriptive | LA | n=52 survey respondents & n=6 interviews with LA SEND professionals and special school staff | Explore barriers experienced by range of SEND professionals and specialist school staff in England when eliciting the voice of CYP for EHCP assessments and Annual Reviews | 36/152 LAs and 16/40 randomly selected special schools responded to the survey; Interview participants opted in via the survey | Most common barrier to including CYP voice in EHCPs was 'barriers to participation' that inhibit their ability to express their views meaningfully (e.g. too young) and limited professional capacity to ellicit CYP views (e.g. resource strain, power dynamics) | Medium quality |
| Palikara, 2018 | At least two methods | CYP | Observational-Analytical | LA | n=184 EHCPs | Analyse depictions of CYP voices in EHCPS and the processes by which these were gathered, for 184 pupils at mainstream and special schools in Greater London | Analysis of EHCPs from 9 Greater London LAs, for a mixture of boys and girls, using inductive content analysis and deductively matched to the ICF-CY | Substantial between LA variability in the process and methods used to capture CYP voices in EHCPs. Type of school influenced how CYP voice was captured, and the extent that methods were described - favouring mainstream schools. Also LA-level differences in depiction of CYP voice regarding: use of first person. There was limited information about CYP abilities and strengths | Low quality |
| **Complaints and redressal** | | | | | | | | | |
| Marsh, 2020 | Quantitative (secondary data) | N/A: secondary analysis of administrative or cohort data | Analytical | LA | Whole population estimates for CYP 2-18 years of age annually for 5 years (e.g. 319,819 in 2018 had EHCPs from an estimated population of 11,183,816) | Provide a statistical overview of new EHCPs and SEND tribunal appeals by examining statistical trends from government data for English LAs from 2013 to 2018 | Used publicly available data to construct 5-year EHCP and tribunal related trends for all of England's LAs, then considered case study LAs and regional analyses. | Large variation between LAs in percentage of EHCPs (5.2% to 1.0%) and tribunal appeals (43 to 0 per 10,000). LAs with lower %EHCPs have more appealable decisions at tribunals and are financed at significantly lower levels than LAs with higher %EHCPs "largely due to an historic spend factor of 50% in the national funding formula." Regional diffences are clear, with largest increases in EHCPs and tribunals in the South East. | Medium quality |
| Lindsay, 2019 | At least two methods | Parents/carers and SEND/education professionals | Observational-Analytical | LA | n=109 LAs & n=74 parent interviews | Examine effectiveness of mediation related to CYP with SEND in the first two years of CFA (2014) enactment: 1) to reach resolutions with no subsequent appeal to SEND Tribunal, and; 2) cost savings of pre-tribunal resolution for LAs and parents | Online surveys of LAs & parent interviews. Cost-effectiveness analysis. Recruited parents who had appealed to SEND Tribunal via forums, snowballing and social media to achieve **purposive sample from 17 case-study LAs.** | 22.4% of mediation cases appealed to the SEND Tribunal vs 36% of non-mediation. Substantial between-LA variation in mediation service contacts and uptake (1-11 and 0-8 per 10,000 school population respectively), and correlation between initial contact and uptake. Complexity of cases strongly influenced costs. Mediation pathway led to lower aggregate cost via lower incidence of appeals and tribunals (£5,231) compared to non-mediation (£5,730). Aggregate costs savings for 1275 mediation cases were >£600,000; average cost saving <£500 per case. | Low quality |
| Cullen, 2019 | Qualitative | Parents/carers | Observational-Descriptive | LA | n=78 interviews | Understand why some SEN disagreements become so distressing for parents, and how such disagreements can be prevented or resolved | Parents with experience of using at least one disagreement resolution process since Sept 1, 2014 were recruited via information leaflets, word of mouth and social media | Reasons for high distress levels around SEN disagreements: 1) Children had significant SEN; 2) Concern over unmet needs; 3) Engaging with statutory processes (demanding, delayed, dissonance between expectation and reality); 4) Number of processes over time; 5) Fear for the future; 6) Cumulative consequences on family life. Ways for LAs to prevent or resolve: 1) Show understanding; 2) Do job properly and listen to parents; 3) Take responsibility to sort it when goes wrong; 4) Invest in LA SEN system; 5) Offer peer support to other parents | High quality |
| **Education and Health Care Plans (EHCPs)** | | | | | | | | | |
| Sales, 2018 | At least two methods | Mixed stakeholders | Observational-Descriptive | LA | n=20: 11 interviews, one FGD, 4 CYP surveys | Identify the extent that reforms including EHCPs) have overcome shortcomings of statementing processes, identify what worked well and 'areas of concern', and offer recommendations to support families through EHCP needs assessments and planning | In-depth semi-structured interviews with parents/carers and SENCOs, an FGD with parents, and a questionnaire with CYP in 2016, analysed thematically. | EHCPs are not always needs based (also influenced by parental resources and advocacy ability, funding constraints and 'high profile' children). There are continuing inequities and inconsistent application of legislation within and between LAs. EHCP outcomes and provision within plans are not always 'SMART'. EHCPs could be strengthened by: quantifying more e.g. number of TA hours/week, involving and valuing parents, and adapting the 'all about me' section for YP for a wider age group and communication difficulties. YP felt included, but scared by meetings. Post-16 transitions were smoother since expansion of SEND age range, but FE institutions need to be more inclusive (extending EHCPs to post-compulsory education), and forward planning further than 12 months ahead. Multi-agency working (health, ed, soc care) works well if all professionals attend the meetings, and was weakest for annual reviews. | Medium quality |
| Richards, 2021 | Qualitative | Education professionals | Observational-Descriptive | LA | n=8 SENCOs | Explore SENCO experiences of implementing EHCPs, and their challenges and opportunities in private, voluntary and independent settings | Three data collection methods/stages: 1) 'Work-lines' to depict positive and challenging experiences of EHCP implementation; 2) Critical event narrative interviews; 3) self-completed repertory grids and ranking 21 salient constructs in order of importance. Content and network analyses to integrate findings and reveal influences over practice. | EHCP implementation and SENCOs: Key aspects of macrosystem (e.g. legislation and policy): lack of senior colleague knowledge of the new system, reduced numbers of area SENCOs for early years provision and fewer trainings & information, particularly when support services provision transferred from the LA to a public company, which increased costs of training courses and employers were less inclined to offer training to staff. Also reduced resourcing of the system. Key exosystem (enactment within local organisations) influences: ethos, support, and quality of evidence. Key microsystem (individual level) influences: knowledge, skills, purpose & outcomes. Key mesosystem influences (i.e. interactions and relationships between different system levels): Relationships with parents and other SENCOs. | Low quality |
| Castro, 2019 | Quantitative (secondary data) | N/A: secondary analysis of administrative or cohort data | Observational-Analytical | LA | 236 EHCPs | In context of recent CFA (2014) implementation, to assess: 1) Quality of EHCP outcomes (function, extent of participation, whether short-term outcomes differ from long-term); 2) EHCP outcome content (how it varies by LA, school type, and type of need) | London LAs were approached to participate in the study. 11 LAs agreed to take part, meetings were arranged with individual schools (n=46) to access EHCPs. Two researchers independently rated EHCPs using a Goal Functionality Scale. | The majority of outcomes were rated as not functional and not high-quality. EHCP outcome quality varies by the LA in which it was developed, school type, and short vs long timeframe. Content varies to some extent on type of need. Authors call for standardisation training and procedural guidelines. | High quality |
| Gaona, 2019 | Qualitative | CYP | Observational-Descriptive | LA | n=12 EHCPs | Examine the methods used to capture views of CYP with ASD about their EHCPs, and whether the resulting 'views, wishes, and aspirations' are functional and match CYP's aspirations | 12 EHCPs for CYP with ASD during their transition to post-secondary destinations were collected from 5 different LAs in Greater London. Section A of each EHCP was analysed using content analysis inductively and matched deductively to codes from the ICF-CY (International Classification of Functioning, Disability and Health Children and Youth Version) | Views and aspirations centred around wishes for greater autonomy (self-care, domestic life, mobility, enhanced participation at home, school and in communities. Support systems (school staff, professionals, families) are strongly relevant at this time. Discrepancies in organisation and content of EHCPs across LAs suggests need for more specific guidelines to develop holistic and person-centred EHCPs. ICF-CY could assist as universal language to discuss views, needs and context for EHCPs and translate into person-specific provision across all life areas | Medium quality |
| Boesley, 2018 | Qualitative | Education professionals | Observational-Descriptive | LA | n=16 SENCOs | Gather SENCO perspectives on process of applying for and transferring EHCPs, identify areas of strength, current challenges, future outlook, potential improvements, and experience and views on multi-agency working and family-led planning. | Interviews with 16 SENCOs from primary and secondary schools, analysed using thematic analysis | 1) Perceived role of SENCO in EHCP process (managing misconceptions and disengagement from services, managing parental expectations); 2) Procedural challenges and changes: an evolving process (regional disparity, lack of transparency in EHCP process, decreased SEN funding, continually evolving process for implementing EHCPs, EHCP system had potential); 3) Difficulties accessing EHCPs for CYP with SEMH needs (over emphasis on academic progress, difficulties validating social, emotional and MH needs) | High quality |
| **Practitioner roles and training** | | | | | | | | | |
| Griffiths, 2018 | Qualitative | Education professionals | Observational-Descriptive | LA | n=24 TAs at 16 primary, 5 secondary & 3 cross-phase schools | Explore TA dyslexia training effectiveness, application in practice, and enablers and blockers of TAs' wider influence on increasing dyslexia awareness | Convenience sample of TAs who had attended a year-long dyslexia training course | Theme 1: taking on a major academic CPD commitment - difficult for TAs to manage; LAs could consider offering different versions with different levels of accreditation. Theme 2: role of the local authority in supporting staff CPD - LAs seen as having critical role in course delivery, and ongoing tutorial support and mentoring. This aspect was positively evaluated by TAs who were part of LA advisory teams, and access to LA specialist advisers and email contact was called a ‘lifeline’ when there was no school-level support or relevant CPD. TAs were concerned this support would be cut with wider LA funding cuts. Theme 3: the paradox of the expert - was school-specific, referring to TAs receiving referrals, but being seen as operating outside of the usual system, difficult to intergrate their skills and share them in the classroom with teachers. Overall, specialist training developed dyslexia knowledge, skills and understanding of dyslexia and application, and enhanced student learning. Required good quality regular liaison between TAs and class teachers (primary settings) or English department (secondary settings). | High quality |
| Curran, 2021 | At least two methods | Education professionals | Observational-Descriptive | LA and MAT | n=15 SENCOs (FGDs), n=1903 SENCO survey respondents | Understand variation in breadth and depth of SENCO role across differing educational settings and phases post-2014 SEND reforms | Online focus groups with 15 SENCOs analysed thematically and used to develop a subsequent online survey, which was piloted, distributed, and thematically analysed | SENCO role is multifaceted, but not well understood by all colleagues. Often involves parallel teaching, SLT and safeguarding responsibilities. Some SENCO's worked across entire MATs, others at school level, with different seniority levels. Authors consider a need for senior-level SENCO in each school to advocate for SEN and inclusive policy and school-wide practice. Most SENCOs felt there was insufficient time for their role and to meet needed SEND provision, role support was inconsistent, and most time was spent on administrative tasks. Most time spent was on data analysis, completing referrals (including to LAs) and annual paperwork, but also meetings, including with external agencies and parents. SENCOs reported a lack of joint commissions and joined up services, with impression that EHCPs are SENCO remit only. This is compounded by lack of agreed local or national time allocations for SENCOs to carry out their work (varied from 0.5-3.5 days per week by SENCO in this study), and lack of standardisation and expectations about paperwork. | Medium quality |
| **School exclusions** | | | | | | | | | |
| Daniels, 2019 | Qualitative | Mixed stakeholders | Observational-Descriptive | LA and MAT | n=27 iterviewees, n=20 interviews | Assess multiple stakeholder perspectives from each of the UK's four jurisdictions on official and informal school exclusion practices, and mutual shaping of policy and practice | Analysis of a subset of data from the Excluded Lives project. Stakeholder interviews (senior policy makers and Government Officers, LA Education Officers, YP who were NEET, lawyers, social worker) analysed with thematic analysis | 1) Policy: changing Independent Appeal to Independent Review Panels combined with push towards academisation led to 'muddied' the roles of academies and LAs for excluded children, and harder for LAs with reduced powers to challenge schools. Some LAs reduced specialist teacher numbers due to the SEND CoP which reduced CYP recorded in the SEN register; 2) School governance - delays of academies reporting to LAs when CYP with SEN are identified; 3) increasing demands which LAs have not adjusted to meet (particularly social, emotional and mental health and Autism Spectrum Disorder), reduced capacity, and financial pressures. Different policy and funding decisions made by different LAs. Multi-agency/professional working constrained by silos between LA departments. Team cuts meant LAs worked reactively. Problems with 'high needs block': if a CYP is excluded or needs a special school, high needs block pays for it and reduces the amount LAs can use to support schools. Adhoc allocation of provision by LAs determined by what was available geographically, rather than being needs-led, and LAs no longer have resources to transition CYP back into mainstream school. | Medium quality |
| **SEND identification and provision** | | | | | | | | | |
| Wood, 2019 | Qualitative | Education professionals | Observational-Descriptive | MAT | n=9 education staff in four academy schools comprising one MAT | Explore the vision for SEND provision of practitioners in a Multi-Academy Trust | Documentary analysis, and a series of iterative and inductive FGDs analysed thematically. | Current ‘identification’ and ‘support and intervention’ of children with SEND, impeded inclusive equitable practice across the MAT and children were not fulfilling their potential. Examples included: inconsistent application of SEND identification systems and processes, despite availability of ‘a range of diagnostic assessments', and variable quality of provision offer by academy. Enablers of good practice: leadership with ‘clear direction and accountability for outcomes of learners with SEND', 'whole-trust, holistic practices...pooling of resources’, ‘sharing of good practice and expertise', where ‘collaboration and collegiality guides the ethos  Barriers: ‘reduction in funding’ ‘under resourcing’, lack of curriculum ownership and effective tailoring for SEND children, external pressures from Ofsted, being held accountable and to 'illustrate strong pupil outcomes’ | High quality |
| Roman-Urrestarazu, 2021 | Quantitative (secondary data) | N/A: secondary analysis of administrative or cohort data | Observational-Analytical | LA | Population-based sample of 7,047,238 pupils | Evaluate whether socioeconomic disadvantage is associated with ASD prevalence and the likelihood of accessing ASD services in racial/ethnic minority and disadvantaged groups in England | Analysis of Spring School Census 2017 from the Pupil Level Annual Schools Census of the National Pupil Database | Significant differences in ASD prevalence exist across racial/ethnic groups, geographic areas and LAs, suggesting potential differences in phenotypic prevalence and/or 'differences in detection or referral for racial/ethnic minority groups. Overall ASD prevalence=1.76%, of which 18.1% had learning difficulties. Overall male-to-female (MFR) ratio=4.32:1. Highest ASD prevalence in Black pupils (2.11%, MFR=4.68:1); lowest in Roma/Irish Travelers (0.85%; MFR=2.84:1). ASD was associated with social disadvantage and EAL. The effect of race/ethnicity on ASD status was mediated through social disadvantage. | High quality |
| Palikara, 2019 | At least two methods | Mixed stakeholders | Observational-Descriptive | LA | n=349 education professionals | Gather views from a mixture of educational professionals about the implementation of the CFA 2014 SEND reforms in England. | Sample recruited via research team networks, including professionals working in special education and mainstream schools, educational psychologists, speech and language services and more. Professionals completing an online semi-structured survey including questions about demographics, training about SEND reforms, and their role in managing changes resulting from the reforms and SEND Code of Practice. Quantitative data analysed with descriptive statistics and statistical tests, qualitative data analysed with thematic analysis | Positive, common views amongst professionals: Extending SEND age to 25 would bridge gap to FE. More holistic assessment would improve cross-service working. EHCP more holistic, person centred and co produced. Negative, common views were about the gap between ideology and implementation. Some questioned the value of changing from statements to EHCPs (partly due to delays 'due to local authorities having to push through changes very quickly' with limited training offered), tendency to focus on education (not health and care), insufficient staffing at local level to transfer statements to EHCPs), poor compliance with 20 week legal limit, greater delays than with statements, SEN department staff burnout due to overwhelm, procedural inconsistencies between LAs and paperwork produced (may include limited and out of date advice, some EHCPs outsourced, some written by people with little knowledge of SEND), lack of funding to support the transition overall. Quality of interagency working is challenging (especially in relation to the EHCP assessment process, which is left up to the LA to decide, and where LAs were given no guidance), and will also determine the quality of the local offer. There was a strong call for 'clear guidelines and systematic, standardised training' to properly implement the new standard. | Low quality |
| **Transition to adulthood** | | | | | | | | | |
| Malkani, 2021 | At least two methods | Mixed stakeholders | Observational-Descriptive | LA | n=75 students | Gather data to develop, co-ordinate and monitor council services and support for young people with SEND as they move into adulthood. Focused on: employment, community inclusion, health and independent living. | FGDs, interviews, and a survey. Thematic analysis (qualitative component), factor analysis for the survey. | Factors for successful post-16 transition: 1) transition programmes started at age 13-14 improved long-term employment chances; 2) continued support at school helped YP more than when solely responsible & asking for help adhoc; 3) involving families in transition planning 4) clear interagency communication (school, social care, health professionals, families) from the start, where all understand YP’s strengths, needs and capabilities; 5) opportunities to meet employers, staff, and see workplaces; 6) providing mentoring and learning opportunities in the workplace, and training employers to faciliate this; 7) opportunities for YP engagement outside of educational settings to foster positive development and behaviours, and sense of belonging and purpose; 8) YP transitioning to adult health systems would benefit from specialised nurse services to liaise/work as multi-agency team with health, social services and school, organise meetings, and help families navigate and understand the health system, and reduce their burden and stress. | Low quality |
| Dunsmuir, 2020 | Qualitative | Mixed stakeholders | Observational-Descriptive | LA | n=16 interviews | Conduct scoping study of professional and manager perspectives on CFA reforms of transitions for YP aged 16–25 with SEND, specifically: 1) differences to CYP outcomes; 2) changes to professional working practices; 3) the extent of ‘communal’ collaboration in practice. | Telephone interviews with a range of professionals and managers involved in transitions within 3 LAs, including educational psychologists, heads/deputy heads of special school/college, health commissioner, SEND and social care managers. Used 'Community of Practice' framework to understand processes of change at local level in response to the introduction of CFA 2014 legislation. | 1) Professional activity, planning and organisation had changed since the CFA: enhanced cross-service communication, coordinated working, service restructuring, co-location, changed lines of accountability; 2) implications for CYP and families were emphasis on long-term planning and involving CYP in decision-making and defining outcomes - especially as YP were placed at the centre of decisions. Challenges that were voiced: (lack of) resources, 'negotiating young people’s wishes' and knowing when participants understood, and navigating tension between child and parent, and child and professional views about educational and living arrangements. Other implementation challenges: bureaucracy around creating EHCPs, resource constraints meaning not all could attend team meetings who needed to be there (as they were busy reconfiguring services), inter-professional teams were established due to legislative change - not self selected - so 'resistance to change in cultural norms and structures' apply here as seen in other studies | High quality |

## Online resource 9. References: Peer-reviewed studies

1. Boesley, L., & Crane, L. (2018). ‘Forget the Health and Care and just call them Education Plans’: SENCOs' perspectives on Education, Health and Care plans. Journal of Research in Special Educational Needs, 18(S1), 36-47. https://doi.org/https://doi.org/10.1111/1471-3802.12416
2. Castro, S., Grande, C., & Palikara, O. (2019). Evaluating the quality of outcomes defined for children with Education Health and Care plans in England: A local picture with global implications. Res Dev Disabil, 86, 41-52. https://doi.org/10.1016/j.ridd.2019.01.003
3. Cullen, M. A., & Lindsay, G. (2019). Special Educational Needs: Understanding Drivers of Complaints and Disagreements in the English System [Original Research]. Frontiers in Education, 4. https://doi.org/10.3389/feduc.2019.00077
4. Curran, H., & Boddison, A. (2021). ‘It's the best job in the world, but one of the hardest, loneliest, most misunderstood roles in a school.’ Understanding the complexity of the SENCO role post-SEND reform. Journal of Research in Special Educational Needs, 21(1), 39-48. https://doi.org/https://doi.org/10.1111/1471-3802.12497
5. Daniels, H., Thompson, I., & Tawell, A. (2019). After Warnock: The Effects of Perverse Incentives in Policies in England for Students With Special Educational Needs [Original Research]. Frontiers in Education, 4. https://doi.org/10.3389/feduc.2019.00036
6. Dunsmuir, S., Cline, T., Crafter, S., & Lang, J. (2020). Challenges in planning transition to adulthood for young people who have special educational needs and disabilities: professional and managerial perspectives. Journal of Research in Special Educational Needs, 20(1), 27-37. https://doi.org/https://doi.org/10.1111/1471-3802.12459
7. Gaona, C., Castro, S., & Palikara, O. (2020). The views and aspirations of young people with autism spectrum disorders and their provision in the new Education Health and Care plans in England. Disabil Rehabil, 42(23), 3383-3394. https://doi.org/10.1080/09638288.2019.1593520
8. Griffiths, D., & Kelly, K. (2018). Beyond the broom cupboard: teaching assistants’ reflections upon the wider impact of their specialist dyslexia training. Reflective Practice, 19(3), 345-357. https://doi.org/10.1080/14623943.2018.1479685
9. Lindsay, G., Conlon, G., Totsika, V., Gray, G., & Cullen, M. A. (2021). “The impact of mediation on resolution of disagreements around special educational needs: Effectiveness and cost effectiveness”. Research Papers in Education, 36(3), 275-298. https://doi.org/10.1080/02671522.2019.1677756
10. Malkani, R. (2021). Investigating the opportunities provided for young adults with special education needs and disabilities (SEND) to prepare for adulthood in a city in England. Support for Learning, 36(2), 238-257. https://doi.org/https://doi.org/10.1111/1467-9604.12353
11. Marsh, A. J., & Howatson, K. (2020). Education, health and care plans and tribunals in England: A statistical tale from 2019. British Educational Research Journal, 46(3), 574-592. https://doi.org/https://doi.org/10.1002/berj.3596
12. Palikara, O., Castro, S., Gaona, C., & Eirinaki, V. (2018). Capturing the Voices of Children in the Education Health and Care Plans: Are We There Yet? [Original Research]. Frontiers in Education, 3. https://doi.org/10.3389/feduc.2018.00024
13. Palikara, O., Castro, S., Gaona, C., & Eirinaki, V. (2019). Professionals’ views on the new policy for special educational needs in England: ideology versus implementation. European Journal of Special Needs Education, 34(1), 83-97. https://doi.org/10.1080/08856257.2018.1451310
14. Richards, H. (2021). EHCP Implementation in the Early Years: constrictions and possibilities. Support for Learning, 36(2), 204-221. https://doi.org/https://doi.org/10.1111/1467-9604.12351
15. Roman-Urrestarazu, A., van Kessel, R., Allison, C., Matthews, F. E., Brayne, C., & Baron-Cohen, S. (2021). Association of Race/Ethnicity and Social Disadvantage With Autism Prevalence in 7 Million School Children in England. JAMA Pediatrics, 175(6), e210054-e210054. https://doi.org/10.1001/jamapediatrics.2021.0054
16. Sales, N., & Vincent, K. (2018). Strengths and limitations of the Education, Health and Care plan process from a range of professional and family perspectives. British Journal of Special Education, 45(1), 61-80. https://doi.org/https://doi.org/10.1111/1467-8578.12202
17. Sharma, P. (2021). Barriers faced when eliciting the voice of children and young people with special educational needs and disabilities for their Education, Health and Care Plans and Annual Reviews. British Journal of Special Education, 48(4), 455-476. https://doi.org/https://doi.org/10.1111/1467-8578.12386
18. Wood, P., & Legg, N. (2020). Special educational needs and disability provision within an English multi-academy trust: capturing the vision of its practitioners. Education 3-13, 48(3), 288-302. https://doi.org/10.1080/03004279.2019.1664405

## Online resource 10. Grey literature study characteristics and AACODS grades (n=102 studies)

| **First author & date** | **Study type** | **Respondents** | **Study design** | **Includes: LA, MAT, Both** | **Sample size & respondents** | **Study aim(s) or research questions** | **Methods** | **Main LA or MAT related findings** | **Overall study grade** |
| --- | --- | --- | --- | --- | --- | --- | --- | --- | --- |
| **Child voice & experience** | | | | | | | | | |
| Ofsted, 2020 | Qualitative | CYP | Observational, Descriptive | LA | 6 'local areas' for SEND 659 phone calls with early years providers between October and November 13 visits to residential special schools in September and October 45 visits to further education and skills (FES) providers in September and October 270 visits to schools between 10 and 19 November, including mainstream schools, special schools and pupil referral units (PRUs) A focus group with four social care inspectors, and written submissions from two social care inspectors. We also aN/Alysed written submissions from two social care inspectors who had completed visits to local areas and residential special schools. | To consider the experiences of CYP with SEND gathered during Ofsted/CQC visits to education, health and social care providers in the context of the COVID-19 pandemic (Sept-Dec 2020) | Inspectors collected information from provider-level leaders and practitioners primarily, with some data from CYP and family member interviews at interim stages only. | CYP with SEND were less likely to attend school/college than peers in the autumn 2020 term, partly due to difficulties arranging school transport (e.g. one special school unable to provide transport for new pupils, despite a timely application). Many CYP with SEND who were attending educational settings, were unable to access the full curriculum, which is a joint LA-school responsibility, often because CYP were given part-time timetables. Short break provision for CYP with SEND was reduced, with reduced staff capacity where providers were open, and other non-LA barriers to attendance, leading to reduced access to enrichment and support. Practitioners said the pandemic had improved multi-agency working to support CYP, likely benefitting those with SEND but risking practitioner burnout. One college leader said their LA links were stronger after developing a rapid-response system to alert relevant LA teams when students were not getting enough to eat. | Low |
| Bradford City Council , 2020 | Quantitative (primary) | CYP | Observational, Descriptive | LA | 49 | This survey formed part of the SEND Local Offer Consultation & Engagement Summary 2019/20. The outcomes survey was conducted to engage CYP in co-producing the LO and develop a Local Area outcomes framework | Snap survey | LA-relevant findings for local offer co-production and development of outcomes framework:  1) Just over half said they felt in control of their lives (59%);  2) About two thirds of participants reported being: happy and having friends (65%), healthy and happy (64.4%), resilient (65.9%), confident and able to acheive their dreams (64%) and thought their voice was heard (64.4%);  3) Around three quarters or more said they: enjoyed good health and well being (81%), felt supported and safe (80%), valued, included and accepted (77%), were able to learn (77.7%), and the people who care for them were enabled to support them (86.6%) | Low |
| Bristol City Council, 2020 | Quantitative (primary) | CYP | Observational, Descriptive | LA | 77 | 1) to better understand CYP experiences of the SEND system in Bristol and; 2) for Bristol council to use survey data help improve the experience of all CYP with SEND and their parents/carers | Not stated | LA-relevant findings:  83% had not heard of Bristol’s Local Offer website; of those who had, 85% had used it, of which one-third found it helpful, 50% partly helpful, 16% very helpful. 50% were involved in the EHCP process vs 40% who were not (10% unsure) Of those involved, 50% said their experience was excellent or good, versus 20% poor.  40% of respondents who have an EHC plan told us they haven’t seen their EHC plan 43% rated their EHCP as very good or excellent vs 14% very poor 57% agreed their plan included what they want to do in the future vs 14% disagreeing 46% of respondents said they needed services that they weren’t getting (covered a range of services, health, therapies, PFA, financial, leisure, equipment, assessments) | Low |
| Scott, 2016 | Qualitative | Mixed stakeholders | Observational, Descriptive | LA | 80 parents, 40 YP, 5 schools, 3 colleges, 3 LA staff, >100 written responses from multiple stakeholders. | To understand the experience that parents/carers and YP with SEND have of school and colleges | Face-to-face meetings with participants in all regions of England, and written feedback. Stakeholders were headteachers/teachers (mainstream and special schools), SENCOs, learning support assistants, parents/carers, CYP, senior LA staff voluntary organisations& service providers. Open, unstructured discussion. Informal analysis: summaries of key points, focused on recurring themes | LA-relevant recommendations: **1) Improve communication, across all agencies, in every area for a ‘person-centred’ SEND system. Promote trust and understanding via proper family engagement, to improve support for CYP, and provide a more realistic understanding of school/college/LA provision.** Many examples of poor communication between: a) LAs (e.g. when families move from one LA to another, reducing access to essential equipment); b) LAs and schools (e.g if LA names a school for a child in an EHCP, but the school tells the family they cannot take them); c) LAs and health services; d) families and LAs (e.g. not all were confident to approach LAs);  **2) More training for staff working with CYP on identifying SEND;**  **3) More transparency over funding** so it is clearer to families how LAs spend money, and challenge LAs to find ways to use contingency funds to meet SEND spending shortfalls;  4) Encourage LAs to interpret and apply CFA legislation and the SEND CoP to change organisational culture and reduce local area variability in provision;  **5) Encourage LAs to provide expertise and improve post-16 opportunities for CYP with SEND** e.g. brokering discussions & developing strategies with employers to improve access to training and employment opportunities. | Low |
| **Complaints and redressal** | | | | | | | | | |
| Bryant, 2022 | At least two methods | Mixed stakeholders | Observational, Descriptive | LA | 6 local areas | 1. What accounts for the rising numbers and patterns of disputes in the SEND system? 2. Where is the system working well and what are the challenges, both within policy and practice relating specifically to dispute resolution, and within the wider system, that are giving rise to disagreements and disputes? 3. What would be needed – and should be considered as part of the SEND Review – to strengthen approaches to avoiding and resolving disagreements and disputes, in terms of policy and practice relating specifically to dispute resolution and to the wider SEND system? | Local areas were selected to reflect a range of tribunal appeal rates, proportions of CYP with EHCPs, deprivation levels, ethnic diversity, geography. Methods included discussions/workshops with SEND service leaders and national bodies, and analysis of publicly available data | **Between 2013/14 and 2020/21:** increasing Tribunal appeal rates, fewer appeals about decisions to carry out EHC assessments, more about EHCP content/education placements, fewer Tribunal appeals are withdrawn, conceded, or resolved via mediation, more Tribunal decisions, which favour families (not LAs). Data insufficiently disaggregated to assess equity of access. **Factors to prevent/manage disputes identified by LA and national staff:** 1) Effective communication, high-quality casework via local culture change to build relationships with families, give timely responses, and person-centred planning; 2) Strong graduated approach, consistent expectation of inclusive practice and SEN support in mainstream settings; 3) Empowering SENDIASS to broker resolutions; 4) Strong inter-agency working and joint decision-making and commissioning (education, health, care each with designated roles reflected in protocols) to provide joined up support pathways; 5) Health, education and care leaders to monitor disputes 'in real-time' and make swift decisions. **Challenges identified:** 1) Proliferation of advocacy groups encourage costly appeals; 2) Inequity of access to dispute resolution though no evidence to support the claim; 3) Unintended consequences of extended Tribunal powers meant CYP with EHCPs got faster access to provision that those with SEN support; 4) Decreasing use of mediation, partly because trend towards placement-related disputes are less amenable to mediation, and poor communication at the assessment stage; 5) Low clarity about key definitions: e.g. ‘efficient use of resources’ (a legal test) and 'effective practice' when LAs must also ensure VFM and manage local high needs block allocations 6) Contradictory reports from professionals of the same disciplines; 7) Vague definitions, thresholds & lack of best practice guidelines to support the SEN statutory framework, e.g. when to carry out EHCNAs, expectations of 'ordinarily available provision'; 8) Increased demands for provision coupled with reduced funding in turn reduced preventative services and increased frustration about lack of early support, and reduced capacity for high quality, co-productive person-centred casework with high staff turnover, fuelling disagreement. All threatens the LT sustainability of the system; 9) Over-dependence on education has undermined the objective to unifiy education, health and care. | Low |
| **Education and Health Care Plans (EHCPs)** | | | | | | | | | |
| Devon County Council, 2018 | Quantitative (primary) | Parents/carers | Observational, Descriptive | LA | 39 parents/carers | To gather parent/carer feedback on EHC statutory assessments | Feedback is routinely requested every time an EHCP is issued (39/411 EHCPs issued - 9.5% response rate for this period) | At least 95% of respondents agreed or strongly agreed that their views were adequately reflected in the EHCP, all relevant professionals contributed to the plan, and gave detailed, up-to-date advice. 79-85% said the process was: clearly explained from start to finish, they were given information about who to talk to if they had concerns during the process, were made aware tof independent information, advice and support services at any time in the process, and of information in Devon’s LO and where to find it. | Low |
| Cheshire East Council , 2021 | Quantitative (primary) | Parents/carers | Observational, Descriptive | LA | 345 parents/carers | To gather parent/carer feedback on the EHC needs assessment process and satisfaction levels | No methods reported except where the survey was promoted (Parent Carer Forum, council’s consultation webpages, social media) | 79% of respondents had CYP who had completed, or were completing, EHC needs assessments. Two-thirds said EHCPs pinpointed needs and appropriate outcomes and felt involved in decision-making. 59% said the right people were involved, 48% said the process was clear, 40% said the process was completed within 20 weeks. Around half of EHCPs had been reviewed in the last 12 months (46% in 2020, 8% in 2021). Authors report that most indicators are improving, though no formal statistics, small sample sizes or consideration of bias. | Low |
| South Gloucestershire Council, 2020 | Quantitative (primary) | Parents/carers | Observational, Descriptive | LA | 14 parents/carers | Questionnaire feedback following assessment for an EHCP | Questionnaire sent out to parent/carers at the end of the 20-week EHCNA process, included rating scale questions and open ended answers/comment boxes. | Most respondents found it easy to understand written communications from the EHCP team, felt involved in the EHCNA process, said case coordinator was helpful, questions were answered fully and quickly, child’s views were considered, their views were valued, and final EHCP described CYP needs. Around half of participants found it very easy to request an EHCNA. | Low |
| House of Commons Women and Equalities Committee, 2020 | Qualitative | Mixed stakeholders | Observational, Descriptive | LA | 17 witnesses gave evidence and 40 written pieces of evidence were received. | To summarise evidence submitted to the enquiry about the provision of EHCPs for CYP with SEND | In June 2020, the committee launched three sub-inquiries about key issues arising from a previous broader inquiry into unequal effects of the COVID-19 pandemic on people with protected characteristics (The Equality Act). Oral and wrriten evidence was reviewed and summarised (no methodological details provided). | Concerns about the relaxed expectations on LAs (“reasonable endeavours” duty): 1) continued too long - many CYP with SEND had little/no support for three months; 2) 'nebulous' in relation to EHCPs - inconsistently interpreted/poorly understood by some LAs, whose support 'fell far short of an acceptable standard', and some examples of LAs not communicating at all with CYP and families. Also concern that lack of 'ring-fenced catch up funding' for CYP with SEND would worsen 'existing disparities in funding and outcomes' compared to peers without SEND. | Low |
| Adams, DfE, 2017 | Quantitative (primary) | CYP and parents/carers | Observational, Descriptive | LA | 13,643 (10,675 parents/carers with CYP <16, 2246 parents/carers answering for CYP aged 16+, 722 YP 16+ self-reporting) | To establish the extent that delivery of EHC needs assessments and planning, and resultant EHCPs reflected intentions set out in the CFA and SEND CoP, how satisfied CYP and parents/carers are, and the extent this varies by LA and particular sub-groups | 65,172 CYP with 2015 EHCPs were identified via the NPD and Individualised Learner Records (publicly funded further education pupils). Invited to take part by letter. Survey was open July-Nov 201, offered online, paper version, face-to-face, easy read, and by phone. Reminder letters after 4 weeks, including a paper questionnaire booklet and a reply-paid envelope, then two week follow up phone call. Weighted to provide England representative picture. | EHC needs assessment and planning process: Two-thirds were satisfied with overall experience. Mixed response if it was a positive experience for CYP, especially younger and from particular LAs. At least two-fifths found the process difficult to start, and had to make >1 assessment request. 20-week plan completion deadline was frequently missed. Inconsistent consideration of families' personal needs and circumstances. CYP felt listened to, but tailoring could be better and include YP more. Half of parents/carers knew about EHCP review process - more likely for those with young CYP. Low use of IASS, the Local Offer and the local Independent Supporter Service. 5% of families had a Personal Budget: less than one-fifth knew about them. **Perceptions of EHCP quality:** largely easy for parents/carers to understand, but less so for CYP (partly age related). Many anticipated a positive impact from EHCPs - more for shorter-term than longer/future, and more for younger than older children. EHCPs for 16-25s were more negatively appraised in terms of likelihood of future e.g. community participation, independent living, aspirations and employment. LAs varied considerably in likelihood of respondents being satisfied or dissatisfied, but no obvious geographic clustering of higher and lower performing. | Medium |
| Torbay Council , 2019 | Quantitative (primary) | Parents/carers | Observational, Descriptive | LA | 7 parents/carers | To gather feedback on EHCP assessment process and resulting plan. | EHCP Questionnaire sent to SENCOs with documentation when there is a request for a statutory assessment. Online version also available. | All respondents agreed: their views were adequately reflected in the EHCP / Annual Review, educational professionals that contributed gave detailed up to date information. Most agreed that: all relevant professionals contributed to the EHC / Annual Review, the plan would effectively meet their child’s needs, the process was clearly explained throughout, they were told who they could talk to if they had concerns and where to get independent information, advice and support from SENDIASS Torbay at anytime. More than half agreed that social care and health professionals gave detailed and up to date information. Plans were sometimes not used by schools. | Low |
| Lancashire County Council , 2021 | Quantitative (primary) | CYP and parents/carers | Observational, Descriptive | LA | 85 parents/carers and young people | To find out about the progress and development of CYP with EHCPs | No methods provided. Online survey was available for completion from March to the end of June 2021. | Over two thirds of participants agreed the support in the EHCP enabled better decision-making, more independence, engagement in education and social participation, and improved their overall well-being. Respondents in mainstream schools were less satisfied than those in specialist provision, CYPs' responses were more positive than adults' (though there were only 18 CYP responses of a small total sample size). Responses collected after schools re-opened following COVID-related closures were more positive than prior. | Low |
| Keer, 2016 | Quantitative (primary) | N/A: secondary analysis of administrative or cohort data | Observational, Analytical | LA | 152 LAs | To establish which LAs outsourced EHCP writing, the number of outsourced EHCPs, and associated expenditure between April 2015 and March 2016. | Secondary data analysis. Assessed live contracts & payments made by LAs to four identified SEN document outsourcing firms using council payments data & contract registers. | At least 42/152 LAs outsourced EHCP writing between April 2015 and March 2016, costing £1.6 million. Wide LA variation in outsourcing and expenditure. Authors suggest reasons for outsourcing include pressure to convert statements to EHCPs by the 2018 deadline, and to meet statutory limit (20 weeks). Lack of transparency means it is unclear how outsourcing influences quality (if freelancers never meet the child) and if it complies with legislation (e.g. data protection, sharing of sensitive documents). Also unclear if it is increasing timeliness of EHCPs being issued, as many LAs still behind. | Low |
| Adams, 2018 | At least two methods | Parents/carers and SEND/education professionals | Observational, Analytical | LA | 25 parents/carers & 10 SEND experts who reviewed 18 EHCPs | 1) Better understand the experiences of CYP and families in LAs with above or below average levels of satisfaction during the EHC needs assessment and planning process and with EHCPs and; 2) Evaluate the quality of EHCPs; 3) Explore the relationship between positive and negative user experiences EHCP and EHCP quality; 4) Identify exemplary practice to drive process improvements and increase EHCP quality. | 1) One-to-one semi-structured interviews with 25 parents/carers, analysed to identify factors related to satisfaction level. Sample included those with above and below average EHCP satisfaction in a 2016 survey, plus information on type of need and sociodemographics; 2) 18 parents/carers provided anonymised EHCPs for quantitative double-review by SEND academics and external moderators/reviewers, blinded to satisfaction level, moderated more than once, scored against criteria from the SEND CoP: adherence to statutory requirements (/120), accessibility (/100), representing CFA and SEND CoP principles (/10). | **Parent/carer satisfaction with process was unrelated to EHCP quality. Satisfaction varied by LA, age, previous SEN statement, need type. Factors linked to higher satisfaction:** 1) One individual can make a huge difference (e.g. one proactive person from an LA or education setting); 2) Dedicated specialist support (to guide families through the process); 3) Having EHCPs ready before a transition; 4) Families and professionals working together, face-to-face; 5) Meaningful involvement of CYP (not 'box ticking'). **Factors leading to dissatisfaction:** 1) Lack of communication from LAs; 2) inaccessible information and support during a complex process; 3) Low transparency about delays and low recognition of their impact; 4) Lack of involvement of families in the process (e.g. in meetings); 5) Low attention to detail and lack of tailoring in EHCPs (copying and pasting, and generic plans); 6) Lack of cooperation between schools and LAs;  **EHCP assessments:** mean score of 91% for statutory requirements, mean score 76% for accessibility, and mean score of 69% for representing principles of COP and CFA. OVERALL, 79% quality rating. Factors leading to better scores: consistent house style, subheadings, use of first person contributions from child and family members. | Low |
| **Local offer for SEND** | | | | | | | | | |
| Bournemouth, Christchurch and Poole Council , 2021 | At least two methods | PCs | Observational, Descriptive | LA | 15 PCs | Identify strengths and weaknesses of current social care section of LO to help improve its useability. | Not stated | 50< used the LO to find social care information. The information on the SEND LO was rated at 5.3 out of 10. >50~~%~~ PCs knew how to request a social care assessment for themselves, however >80% didn't find the social care pathway clear. | Low |
| Surrey Additional Needs and Disabilities Partnership, 2021 | At least two methods | Mixed stakeholders | Observational, Descriptive | LA | 7 not stated | Feedback on LO email enquiry service following mystery shopping exercise. Also, gather experiences and improvements for LO website through user testing. | Not stated | Contacted person provided helpful, clear, and caring advice. There were mixed responses regarding waiting times. The majority of participants were happy with the service. | Low |
| Lancashire County Council , 2017 | At least two methods | PCs | Observational, Descriptive | LA | 28 PC's | Find families views about FIND newsletter. | Survey sent to families included in database with the Autumn 2017 issue of the FIND newsletter, copies were taken to local parent carer forums and information was put on social media | The majority of participants found the FIND Newsletter useful. 25% were interested in writing or editing future newsletters. Multiple respondents suggested more information on CYP. | Low |
| Slough Borough Council, 2020 | Qualitative | CYP | Observational, Descriptive | LA | 10 CYP | To gain local students opinions on Sloughs Family Information Service website. Also, to ensure CYP know how to find and use the current LO. | Not stated | None of the respondents knew what the LO was or how to access the LO. Respondents suggested multiple design changes to the LO website including improving visual appeal and reducing word count. The CYP were interested in clubs in the local area. | Low |
| Northamptonshire County Council , 2017 | At least two methods | Mixed stakeholders | Observational, Descriptive | LA | 34 PCs, 15 SENCOs, 1 CYP, 12 Not stated | Assess use of LO and LO website. Also, to gain feedback on LO and LO website to guide improvements for users. | Survey shared on social media, live on the LO for March 2017 and shared via informer and schools emails | The majority of respondents had found and used the LO. Of the >50% who found the information they were looking for, the majority found the information useful. The majority found social media useful in finding LO information. | Low |
| Doncaster Borough Council , 2017 | Quantitative (primary) | Education professionals | Observational, Descriptive | LA | 24 SENCo survey & 31 SENCo meeting | Collect feedback about the LO | Events and meetings organised with various groups across the borough to collect feedback about the LO | About 15% of SENCOs found it difficult to access the LO and could not find the information they needed. >50% had difficulty finding the information they needed. >85% found the information useful and easy to understand. <95% were likely to use the LO website again. | Low |
| Hodgson, L. (Derbyshire Parent Carer Voice), 2021 | At least two methods | PCs | Observational, Descriptive | LA | 80 PCs | Views of parent carers of CYP with SEND in relation to the review of the Derbyshire LO | Not stated | The majority of PCs accessed the LO for EHCN assessment information. The website was rated about 3 out of 5 for visual appeal and easy of use by PCs and their children. | Low |
| Plymouth City Council , 2019 | Qualitative | Mixed stakeholders | Observational, Descriptive | LA | 40 PC, CYP and SENCos not stated | Gather PCs, CYP, and volunteers feedback on Plymouth LO to guide improvements. | Parent group meeting, | The majority of PCs could find the LO and found the information useful. >75% said the information was not up-to-date. Many PCs felt the LO website could be simplified. | Low |
| Devon County Council, 2022 | At least two methods | Mixed stakeholders | Observational, Descriptive | LA | 7 CYP, 77 PCs, 4 SBPs, 60 CYP Volunteers | To get PCs, CYP and professionals views on the LO to improve it. | Not stated | >50% of parents do not access funding for activities, where less than a quarte of parents cannot afford the activities. The largest barrier to doing activities is that many do not welcome additional needs for CYP. Funding is the main area of support short breaks providers require. | Low |
| O'Malley, M. (Essex County Council), 2021 | At least two methods | PCs | Observational, Descriptive | LA | 400 PCs, 20 interviews, 3 FGs | Explore experiences and issues around the current short breaks offer and use these insights to help shape the future service offer | For interviews, participants selected to represent a diverse sample of families, with consideration given to distribution across districts, as well as children’s ages, disability, and services accessed. | Lack of access to the short breaks service is mainly due to staffing issues, complicated family needs and unequal provision across Essex. Lack of local activities was the largest barrier for families. More information needs to be provided for disability services for short breaks such as "Light Touch Support". Many families found it difficult to access the information they needed to find. | Low |
| Herefordshire Council , 2018 | At least two methods | PCs | Observational, Descriptive | LA | 125 Families | Gather PCs and CYP experiences of the Short Breaks offer to make further improvements. | Families with children receiving short breaks since April 2017 | >75% of respondents rated the service at 5 out of 10 or more. The majority of respondents reported that the short break was safe and fun, whilst helping to foster independence and prepare the CYP for adulthood. The majority of families felt less pressure where the short break allowed the family to spend time together. | Low |
| North Somerset Council, 2019 | At least two methods | Mixed stakeholders | Observational, Descriptive | LA | 60 CYP, 44 PCs, 7 professionals, 1 SENCo | Views of CYP under the age of 18 with SEND, parents and carers who have children under the age of 18 with SEND and professionals who work with CYP under the age of 18 who have SEND were sought, have their say regarding the current short breaks offer | Participants recruited at drop in sessions | CYP were of varying ages and locations. More than 2/3 of CYP enjoyed the activities they did. The main activities enjoyed were having a buddy and clubs. PCs were the CYPs main way of finding out about activities. >50% of CYP couldn't attend an activity, often due to lack of support at the activities. The majority of respondents felt unsatisfied with the current short breaks provision. The majority of respondents did not know how to access the LO. There was mixed opinions on whether the current short breaks services were helpful or not for families. | Low |
| Oldham County Council, 2017 | At least two methods | Mixed stakeholders | Observational, Descriptive | LA | 50 CYPs, 166 PCs, 38 PCs in FG's | Gather short breaks experiences from PCs, CYP, volunteers, and professionals to improve the future effectiveness of local joint working. | Survey distributed electronically and in hard copy to PC, CYP and providers, distributed via current short breaks providers, LO and Action Together website, parent carer forum, parent and carer forum’s annual conference, 4 focus groups with PC of CYP who access short breaks or have personal budget, Barrier Breakers supported members to complete survey, Action Together followed up 34 parents who contacted about the engagement from the Local Authority, Current short breaks providers invited to attend consultation interview, of which 5 attended and a digital survey was circulated to providers, 5 of whom completed the survey | 87% of CYPs accessed short breaks, where both PCs and CYPs rated sports and trips as the most helpful. No CYPs rated their current short breaks negatively. The majority of CYPs did not have a personal budget. The majority of PCs said their children attended short breaks, however many PCs felt their CYPs individual needs were not met. Of the families who receive a personal budget, the majority felt that more group based and weekend provision would be prefered. Many providers felt that there was poor advertising of short breaks services. | Low |
| Telford and Wrekin Borough Council, 2021 | At least two methods | PCs | Observational, Descriptive | LA | 91 PCs, 21 SENCos, 3 CYP, 2 not stated | Gather feedback on short breaks offer to guide future improvments. | Not stated | The majority of PCs found the short breaks statement easy to understand. 19% of respondents did not participate in short breaks activities. >35% of respondents said that short breaks activities were inaccessible. The short breaks activities moderately met respondents needs. Parents of children over 18 had more difficulty having their childs needs met by short breaks. Life skills and sports were the most desired short breaks activities. | Low |
| Wakefield City Council, 2018 | Quantitative (primary) | CYP | Observational, Descriptive | LA | 102 CYP | Gather CYP feedback on Short Breaks and desired activities. | Feedback gathered during engagement events | Sports activities identified as the most popular activities to implement. Activities surrounding technology (i.e. computers) identified as the most popular creative activities. The majority of CYPs enjoy their current short breaks provision. The most popular short break was group activities outside of school. | Low |
| MacDonald, C (Northumberland County Council). , 2021 | At least two methods | Mixed stakeholders | Observational, Descriptive | LA | 80 PCs, 40 CYPs, and 2 SENCos | Gather PCs, CYPs and SENCos feedback on short breaks provisions. | The NCC communication team, forums and local services promoted the links via social media platforms and within their networks (PC). Survey links on the home page of the SEND LO. Survey promoted alongside the PC survey, two SENCOs supported CYP to complete the survey (CYP). A short online survey shared with members of a CYP's disability group, shared it within their professional networks (SENCos). Survey links shared with colleagues within the Clinical Commissioning Groups, Disabled Children’s Team , SEND champions and each Early Help Locality Team, to share within their networks and promote via their social media pages. | 70% of CYP had not received a short break service, with the main barrier being limited information or support. The main way families found short breaks services was through personal research. The average family found it moderately difficult to find and access short breaks services. The majority of PCs felt that there were a limited number of short breaks available, particularly those that met their CYPs needs. 79% of CYP wanted to do more activities away from home such as clubs. Health professionals rated the availability of and suitability of short breaks services as <50%. | Low |
| Westminster City Council, 2021 | At least two methods | CYP | Observational, Descriptive | LA | 8 CYP surveys, 6 CYP in FG, 6 CYP interviews | Gather CYP experience with transitions and short breaks | Not stated | Schools identified as generally helpful, however they could work harder at encouraging equality. Learning support identified as helpful. Sports activities the main short breaks activity. Creating autism friendly environments identfied as important for CYPs. | Low |
| South Gloucestershire Council, 2021 | At least two methods | PCs | Observational, Descriptive | LA | 137 PCs | Assess LO usage and gather how LO is being found and accessed. Also, to test useability of LO website with PCs. | Not stated | The majority of participants found the LO, with the majority of those finding the LO difficult to use. Although, 77% found the majority of information they needed. | Low |
| St Helens Borough Council, 2017 | At least two methods | PCs | Observational, Descriptive | LA | 8 PCs | Assess PC useability of LO website and gather feedback to improve ease of use. | Members of the parent and carer forum Listen 4 Change | Half of PCs had previously accessed the LO, where the ease of use and relevance of LO information was ranked is minorly disatisfactory. The PCs found the website design as satisfactory. The case studies evidenced mixed success in finding LO information with the PCs. | Low |
| St Helens Borough Council, 2017 | At least two methods | CYP | Observational, Descriptive | LA | 40 CYP | Gather experience of the LO and LO website. | Sessions held in educational settings (4 primary schools, 1 secondary school), schools asked for groups of around 8-10 pupils (children with SEND, siblings of children with SEND and any other pupils). Session held during an 818 Youth Activity Group for those with Special Educational Needs and Disabilities | Only a minority of CYP had heard of the LO and a quarter used it. No participants had ever used the site. Many CYP would use the LO site to access information. Use of social media would increase awareness of site. Many CYP use their local library service. CYP feel they can talk and engage with key school staff members. Interest in having more charitable work connected with the LO site and local fund raising events. CYP reported that there were plenty of activities in St. Helens, and generally they could find this information on the LO. Majority of participants found it easy to find information and navigate the LO site. Most frequent issue raised was the aesthetic of the LO website. Name "LO" liked by participants. | Low |
| Staffordshire County Council, 2022 | At least two methods | Mixed stakeholders | Observational, Descriptive | LA | 10 PCs, 45 not stated | Gather feedback on LO website to improve accessibility to users. | Not stated | The majority of CYPs had not heard of the LO and had never used the LO before. The majority of CYP felt that there were plenty of activities in the local area. Many CYP suggested design changes to the website, particularly adding more colour, images, and eductional games. Some CYP found the LO website structure confusing. | Low |
| Staffordshire County Council, 2019 | At least two methods | CYP | Observational, Descriptive | LA | 19 CYP | Gather CYP feedback on LO website to make the website more appealing and easier to use. | CYP took part in workshops and user testing sessions coordinated by the Voice Project, SENDIASS and the Visual Impairment service | >50% said LO website information was easy to find and understand. Many respondents suggested making the website more child friendly by simplifying language and adding more pictures. | Low |
| Staffordshire County Council, 2021 | At least two methods | CYP | Observational, Descriptive | LA | 20 CYP | Gather basic demographics and gain feedback on LO and LO website. | CYP took part in survey and user testing sessions via the Voice Project, and SEND for our Voices | CYPs wanted the LO website to be more child-friendly. Respondents found it nor easy or hard to find information on local activities. Respondents were primarily <16 years old. | Low |
| Torbay Council , 2015 | At least two methods | Mixed stakeholders | Observational, Descriptive | LA | 32 professionals, 5PCs | Improve current LO's dissemination, ease of use and usefulness for users. | Survey publicised using a number of methods including a press release, the Parent Participation website, SEND Reforms newsletter, team meetings, Facebook, Twitter and email | Low response rate highlights lack of awareness of the LO. PCs and professionals believe the website needs changes to its design such as adding photos and reducing length of text. Professionals found it easier to find the services they required than PCs. | Low |
| Torbay Council , 2019 | At least two methods | Mixed stakeholders | Observational, Descriptive | LA | 23 SENCos | Gather SENCos feedback of LO. | Not stated | The majority of SENDCos were satisfied with the RSA and annual review process. >90% of SENCos felt satisfied with the SEN team service. However, support with SENDCo work could be improved. Lack of wordiness and use of SMART goals identified as crucial for a EHCP. | Low |
| Wakefield City Council, 2016 | At least two methods | Mixed stakeholders | Observational, Descriptive | LA | 42 not stated | Summarise improvements made to LO and short breaks. Also, receive feedback for LO website to guide improvments. | Questionnaire circulated to Parent/Carer, Young People, Schools, Colleges, NHS and Voluntary/Community sector groups, available in LO website | >60% rated the appearance and content of LO website rated as "good" or "very good". Resondents mostly found the information easily and found the information relevant. The website layout was highly praised. | Low |
| Leeds SENDIASS (Leeds City Council), 2021 | At least two methods | Mixed stakeholders | Observational, Descriptive | LA | 7 CYP, PC Not stated | Gather young people's thoughts about the SENDIASS website, and parents' thoughts about SENDIASS support. | Not stated | The majority of CYP found the website useful and found the information they eeded. Parents found fairly easy to get in touch with SENDIASS and have their questions answered. | Low |
| Child Friendly Leeds (Leeds City Council), 2021 | Qualitative | Mixed stakeholders | Observational, Descriptive | LA | 5 CYP and 6 PCs | LO website feedback for ease of finding information and improvements. | Not stated | CYP and PCs liked the website design and found the information easy and clear to find. | Low |
| Reading Families Forum, 2022 | Quantitative (primary) | Mixed stakeholders | Observational, Descriptive | LA | 50 PCs, 6 SENCos, 3 volunteers, 4 not stated | Assess current use of LO and current interest in regular email contact with newletters. | Not stated | The majority of respondents had accessed the SEND LO, half of which did so to find out about EHCPs.>75% found the information they required, but <65% wanted more information on short breaks sevices. >60% wanted to receive more information about the SEND local offer. | Low |
| Smith, P. (Nottinghamshire County Council), 2021 | At least two methods | Mixed stakeholders | Observational, Descriptive | LA | 1 CYP, 20 PCs, 13 not stated | Gain feedback from PCs, CYP, and professionals on their experience of the LO website to guide improvements. | Survey advertised through various channels including NCC Corporate Communications, IRIS Magazine, the LO website, Families Information Service Facebook page, Nottinghamshire Parent/Carer Forum, Special Schools, Early Childhood Services, Education and Health partners | The majority of respondents found the LO website and information easier to find than not, and mostly accurate. The majority said they were likely to visit the LO again, although only <40% were saitisfied with the experience using the LO. >50% of respondents suggested improvments for the LO. | Low |
| **Practitioner roles and training** | | | | | | | | | |
| Swindon Borough Council, 2021 | Quantitative (primary) | Mixed stakeholders | Observational, Descriptive | LA | 187 | Gain feedback from practitioners and professionals who work with CYP and families with SEND around their training needs | No methods reported aside from statements about when the survey was open, and the comparison against 2019 data. | High confidence about: making positive relationships with CYP, parents/carers and other practitioners; working co-productively with CYP and families; multi-agency working to meet needs of CYP and families; using person-centred approaches, effective listening and structured conversations with CYP and parents/carers; seeing past CYP disability to promote their strengths. Training/information needed about: personal budgets, including explanations to parents/carers; discussions about preparation for adulthood from younger ages; accessing the complex case panel; explaining different types of participation; contributing to the LO and explaining it to others; explaining the legal framework around EHCPs, and the SEND and Inclusion Strategy (2020-23) | Low |
| **School exclusions** | | | | | | | | | |
| House of Commons Education Committee, 2018 | Qualitative | Mixed stakeholders | Observational, Analytical | LA | 100 pieces of evidence submitted to the enquiry | To examine school exclusion rates of pupils from mainstream schools and referals to alternative provision. | Drew upon secondary data and evidence submitted from a range of stakeholders to the call for evidence by the House of Commons Education committtee. No information on how particpants were identified, sampled or recruited. | Rise in ‘zero-tolerance’ behaviour policies may be driving increases in punishments and exclusions that should be managed in mainstream school environments, and disproportionately affect disadvantaged children; Children and their families who are directed off site or are excluded have no system of redress, face an adversarial system, and have no say over new placements; LAs have low awareness of provision in their area for alternative placements, do not make appropriate commissioning decisions, which in turn do not face scrutiny, leading to inappropriate placement decisions; inconsistent exclusion processes and referral to AP; Lack of moral accountability or incentives for schools to retain pupils who may be challenging; lack of transparency about exclusion rates when parents/carers choose schools; LAs have little oversight or scrutiny over school decisions to exclude, placement decisions, or checking unregistered providers. | Low |
| **SEND identification and provision: inspectorate led research** | | | | | | | | | |
| Hayter, S., 2017 | At least two methods | Mixed stakeholders | Observational, Descriptive | LA & MAT | Not stated | To investigate the effectiveness of SEND reforms in Leeds. | No information about participant identification, recruitment or sampling. Methods carried out by Ofsted/CQC inspectors included: direct discussion with paricipants, an online webinar with parents/carers, a desk review of the local area's self-evaluation and performace data, and the local offer. | Local area works with MAT to assess each YP to ensure needs are met. Leaders have developed services for the youngest vulnerable children for early SEND identification. The majority of parents who attended EHCP assessment meetings were satisfied with the outcome. 90% of new EHCPs are completed within 20-weeks. There is often inconsistent identification and provision for CYP SEND. Short timeframe to convert statements to EHCPs lead to delaying reviews. School nursing service inadequately commissioned, and health checks not completed at key transition points for CYP. Parents report that CYP social needs are not accurately assessed or met. Many EHCPs lacked analysis of barriers to meeting outcomes. Very low awareness of the local offer. | Low |
| Ofsted , 2017 | At least two methods | Mixed stakeholders | Observational, Descriptive | LA | 30 LA inspections | To identify strengths and weaknesses of SEND provisions found from 30 LA SEND inspections. | The framework for these inspections sets out how Ofsted and CQC jointly inspect the local area’s effectiveness in three main aspects: • Identifying children and young people’s SEND • Meeting the needs of children and young people who have SEND • Improving outcomes for children and young people who have SEND. Inspectors assess how well local areas are preparing these children and young people to live as independently as possible and, where possible, secure meaningful employment as they move into their adult lives. This inspection framework holds local area leaders to account for how they implement the Code of Practice and for their strategic leadership of services in the local area. In particular, inspectors evaluate how well the implementation of the Code leads to improvements in: • Identification of SEND • Providing for and meeting needs • Outcomes for children and young people who have SEND. | SEND children had poorer experience of education including more abscence and exclusion. Some ares had limited CAMHS and therapy options. A lack of co-production in more than 1/3 areas. More than 50% of parents did not help parents access SEND information. There was good short breaks access for SEND children. | Low |
| Ofsted, 2021 | At least two methods | Mixed stakeholders | Observational, Descriptive | LA | 44 PC interviews, CYP and carers not stated. | Examine experiences of SEND support for CYP during the pandemic. | 6 ‘interim visits’ to local areas, and 4 more during the spring term 2021, Used a case-study approach that focused on a sample of children and young people with SEND to examine their experiences of support during the pandemic. During these visits, inspectors spoke to the parents and carers of 44 children and young people, along with the education, health and social care practitioners who worked with them. We also invited children and young people to take part in interviews, but very few did.  Inspectors also held discussions with education, health, social care and children’s services leaders from the 10 areas we visited. This included senior officers from the local authority, clinical commissioning groups and NHS services such as therapy providers, and child and adolescent mental health services (CAMHS).  We asked the local areas to distribute surveys to parents, carers and young people to gather their views and experiences of the support they received during the pandemic. Inspectors shared key findings in meetings with local area leaders during the visit.  This report also draws on Ofsted’s findings from our visits to schools, early years providers, further education and skills providers and children’s social care providers and the subsequent reports we published in autumn 2020, as well as a range of other reports that have been published during 2020/21. | Needs for SEND children had been better met in key stage 1 than in 2 and 3. SEND pupils are absent and excluded significantly more than non-SEND pupils. The majority of parents felt that their children have not received the support and services they needed. The lack of joint commissioning and poor EHCP quality are significant issues. | Low |
| Ofsted , 2016 | At least two methods | Mixed stakeholders | Observational, Descriptive | LA | 741 CYP and 1,223 PC Questionnaires, Webinars 116 PC's, Webchat 40 PC's, 500 participants at seminars, 11 emails, 5 inspections. | To inspect how effectively local areas fulfil their responsibilities to CYP who have SEND. | The consultation was open to the general public and promoted widely through the Ofsted, CQC and DfE websites, conferences and media .They used a variety of methods to gather views for the consultation. These included two questionnaires: one specifically for children and young people to complete; and one for all other interested parties, such as parents and carers, professionals, institutions and organisations, voluntary and charitable groups, including children and young people. Additionally, counsultation was also made through webinars, a webchat session and face-to-face events with professionals from the health, education and social care sectors, parent representatives and the voluntary and community sectors. Also  received responses by email. No information on pariciapant identifiaction, sampling or recruitment. | 90% of respondents were supportive of the proposals to identify, meet and analyse SEN childrens progress across transitions. | Low |
| Ofsted, 2021 | Qualitative | Mixed stakeholders | Observational, Descriptive | LA | 20 CYP, 21 PC's, 7 HT's, 7 SENCOs, 21 teachers and 20 LSA's, and 2 LA's at interview. | To understand how are the needs of SEND CYP are met in mainstream schools. Also, how do schools approaches to identifying and supporting SEND CYP change between providers. | Ofsted research team carried out qualitative interviews with co-produced topic guides with 21 non-randomly sampled pupils with SEND from 7 mainstream schools in 2 local authorities, 7/21 had EHCPs. Mix of primary and secondary age. Focus groups with LA and CCG professionals. | Those with SEND often missed parts of the curriculum due to time out of class and exclusion from activities. Multi agency collaboration was limited and caused delays in identifying SEND. There wer clear inconsistencies in provision such as multi agency support, progress in school and inclusion activities. | Medium |
| **SEND identification and provision: LA-led or commissioned research** | | | | | | | | | |
| Hall, K., 2017 | Qualitative | CYP and parents/carers | Observational, Descriptive | LA | 109 CYP, 124 PC, professionals n.s | Gather feedback about SEND services from CYP with SEND and their PCs in Leicestershire. | Meetings at youth clubs, maintained special and independent schools, summer activities, forum settings, support groups and a film project. Informal interviewing techniques to ask questions. | Parents were confused as to what service were available to them. They felt that most information about the local offer is spread by word of mouth. Parents felt that mainstream schools had a lack of understanding of ADHD and ASD. Parents felt fruistrated that private reports were not involved in the EHCP process. Post-18 support is poor which may be helped by multi-agency collaboration. Respondents noticied inconsistencies in SEND provisions in different schools. | Low |
| Deacon, A., 2017 | At least two methods | Mixed stakeholders | Observational, Descriptive | LA | 29 EYP's | Includes: Childminders and Schools with Nursery and Reception Classes to understand more about SEND children aged 0-5 accessing education; and Feedback from parent/carers of children with SEND who may be currently entitled to Early Education or have previously been entitled to Early Education | Private, Voluntary and Independent (PVI) Early Years Settings including childminders and Early Years Foundation Stage Leaders within Reading maintained schools; and Parent/carers of children with SEND who may be currently entitled to Early Education or have previously been entitled to Early Education | 1/3 of care settings were unsure on how to apply for funding. Practitioners stated that additional training on early years difficulties and communication needs would improve provision for SEND children. Practitioners felt a lot of uncertainty in finding resources to support SEND children. Most parents felt unconfident in finding care to suit their child's needs. 76% of parents felt informed about their children's progress. | Low |
| Cheshire East Council , 2021 | At least two methods | Parents/carers | Observational, Descriptive | LA | 345 PC's | Gather PC feedback on ASD assessment. | Survey promoted via: -The Cheshire East Parent Carer Forum -The council’s consultation webpages -Social media, including via Twitter and Facebook. | Positive ratings were received about written reports, information about childrens referral, venue for appointments and signposting. However, there were negative reviews on the clarity of information and providing information about who to contact and access to support. There was also negative feedback on the time to receive the firt appointment and the wait for the initial assessment. The majority of categories had a small improvement from 2020 to 2021. | Low |
| Cambridgeshire County Council , 2018 | Quantitative (primary) | CYP | Observational, Descriptive | LA | 52 CYP | Assess if the Cambridgshire SEND support improves CYP's quality of life. | No methods. | The majority of respondents felt that the support helps to make new friends, build confidence and new skills, and spend time away from family doing local activities. The themes that young people struggled with were related to being around large groups of other people and uncertainty about activities. | Low |
| Peterborough City Council , 2020 | At least two methods | CYP | Observational, Descriptive | LA | 12 CYP | To understand what improvements CYP would like to see in SEND communication. Next, to identify the most effective ways to inform CYP with SEND to access the support and information they need. | Survey sent by the communications team to students with SEND at City College Peterborough | >90% of SEND children found it difficult to find SEND information. They preferred method to receive the information was through social media videos. | Low |
| Booth, D et. al., 2019 | At least two methods | Parents/carers | Observational, Analytical | LA | Not stated | Assess the current and future needs of the Lancashire SEND population. Also, to identify gaps current SEN provision and support. | In mid-2018 a project group of data and intelligence specialists from across Lancashire began the process of data gathering and analysis, underpinned with a broad literature review of international and national evidence on SEND. As part of the JSNA a data dashboard has also been developed to present the data relating to the educational, health and care needs of the SEND population. This was largely on 2ndry data. Primary data was gathered from parents and carers to obtaine their view points on SEND provision and support. however, no information on sampling, partcipant recrruitment etc. | The SEND cohort is having an increase in percentage of absentees. CiN are more likely to have a longer case duration when they have SEN. Looked after SEND children were more likely to have a full care order. Children with SEND were more likely to be deprived. | Low |
| Bournemouth, Christchurch and Poole Council , 2021 | Quantitative (primary) | CYP and parents/carers | Observational, Descriptive | LA | 193 PC's and 37 CYP | Understand experiences of SEND services across health, education and social care. | Not stated | The majority of parents knew how to access mental health and educational support for their CYP. Most parents feel like their views have been included in their CYP's care and EHCP. The majority also felt happy with support from the educational and mental health support provided. Most did not feel happy that they were listened too or well cared for by the social care services. Most parents felt that their child did not receive all the support they needed. Parents also felt that there was a lack of communication. Parents stated that they were not confident in the support for their childs transition to adulthood. The majority of CYP felt safe, supported and secure in education and their local community. The majority felt knew where to fnd information about staying heathy, work and activities outside of school. Most young people felt involved in decisions made about them. | Low |
| Atkinson, E., 2018 | At least two methods | Mixed stakeholders | Observational, Descriptive | LA | 50 EHC professional interviews, 27 EP surveys, 155 PC interviews, 67 PC surveys, 49 CYP interviews. | Assess effectiveness of current SEND provisions in Blackburn and Darwen across settings. | Survey circulated to head teachers and SENCOs we met with managers and staff across children’s education, health and care services, parents and young people, head  teachers, deputy head teachers, further education staff, and SENDIASS, with a follow-up survey in early January for all schools and settings to ensure as many people as possible could contribute to the review. As part of the review, Blackburn with Darwen set up an advisory group to shape the review  and consider emerging themes. NDTi staff attended these meetings, as well as exploring a  range of relevant information about the SEND system in the borough. This included the  Local Offer, relevant local strategies and policies, data provided locally and by DfE, based on  the returns by the local authority.In addition, we have reviewed a wide range of documents, including a  sample of 10 EHC plans. The data and information gathering stage progressed on two fronts: > interviews with education, health and care professionals and young people > interviews with parents and carers of young people with SEND currently in the  education system.We reviewed the Local Offer, local policy documents and national data sets during  November and fieldwork continued until 18th December, with a further online survey  completed up to the end of January 2018. Qualitative data was compiled through face to face interviews, focus groups discussions,  telephone interviews, online and paper questionnaires. how sampling was carried out is not mentioned. parents were approached through foloowing channels: >Local Parent Carer Forum, Blackburn with Darwen Parents in Partnership (PIP) >Friends of the Local Offer group  >Parents at an evening Transition Event >Focus group at Crosshill Special School >Focus groups with Blackburn with Darwen parent carers from Children’s Centres Early  Years groups and Special Schools >The online survey was promoted by SENCOs and a local support group, | Schools found that inclusivity of SEND pupils becomes more difficult in secondary school due to a widening attainment gap and focus on exam results. 65% of respondents saw the SEN support services in a positive light. Provision for pupils with more complicated SEND in mainstream schools was poor. | Low |
| Hackney Learning Trust, 2017 | At least two methods | Mixed stakeholders | Observational, Descriptive | LA | 38 families and 52 professionals | Sought views of professionals and parents and carers of Hackney CYP with SEND regarding SEND provision in Hackney. Intention of survey: 1. Gather views regarding the sufficiency of current provision in Hackney for CYP with SEND. 2. Seek ideas for possible adaptations or extensions that could be funded by the DfE capital grant to increase provision | Online survey published and promoted via the Hackney LO website consultations page, downloadable version made available on the LO, print copies were made available for collection in HLT reception or via post upon request | The majority of parents and professionals felt that education provision was not sufficient. More than 70% parents said that their children had experienced barriers to education. | Low |
| North East Lincolnshire Council, SEND North East Lincolnshire, NHS North East Lincolnshire Clinical Commissioning Group, 2021 | At least two methods | Parents/carers | Observational, Descriptive | LA | 107 PC | Gather PC experiences of SEND services for CYP. | Not stated | The majority of parents said their children were well supported to reach goals and move between settings. Most respondents did not find the local offer useful and did not know which services will support their children post-16. | Low |
| Portsmouth City Council , 2021 | At least two methods | Parents/carers | Observational, Descriptive | LA | 681 families | Help understand current issues with SEND provisions in Portsmouth to develop and shape future SEND services. | Survey promoted through Social media, Portsmouth City Council website, Portsmouth LO website, Email distribution lists,Newsletters,Through schools and health services | About 1/3 children received planning support for post-16 education. Over 2/3 children had an ECHP, where children with EHCPs were more likely to use a short breaks service. Most parents felt they were listened to and said their child was receiving the support to help them learn and progress in education. 42% of parents with SEND children said their children had mental health issues. Parents felt that there were delays in receiving support for SEND and that schools do not offer enough support. | Low |
| Vincent, J. & Cross, J., 2021 | At least two methods | Parents/carers | Observational, Descriptive | LA | 273 PC's | To understand the current views of PC on SEND provisions and reforms in Portsmouth. | Online open survey on the Council’s online consultation hub, survey and a feedback flyer promoted via the Sefton Parent and Carer Forum membership and a network of distributors including school’s admin teams and all school’s Headteachers for SENCO’s, SEND Co-production, Communication and Engagement Sub Group partner members, Every Child Matters Forum Coordinattor, Aiming High Coordinator, Head of Education Excellence (for School’s newsletter), Healthwatch Sefton (for website), NHS South Sefton and NHS Southport & Formby CCG (for website), Sefton council social media, promoted on LO, completed over phone | Parents and carers feel listened to, however they felt that SENCOs could improve contact with them. The majority of respondents had not recently used the local offer. Respondents were mostly unaware of social care service including the EXCP process. The lockdown has caused significant delay in asessments and support. | Low |
| Swindon Borough Council, 2022 | At least two methods | Mixed stakeholders | Observational, Descriptive | LA | 261 PC's, 97 professionals, 28 CYP | Understand the experience of young people, their parents/carers and practitioners delivering services to kNow if improvement plan is working, and capture their thoughts from the pandemic | Survey promoted through SEND Families Voice, STEP, SEND News Splash, SBC & CCG newsletters & SENCOs | Their are inconsistencies with the quality that SEND services provide to different families. Parents of SEND children are dissatisfied with time taken to arrange assessments and see clinicians. Parents recognised the lack of communication between different services. Parents desired more funding for SEND services as they often struggled to contact services promptly. | Low |
| McCaffrey, M & Robinson, P., 2020 | At least two methods | Parents/carers | Observational, Descriptive | LA | 30 families | Gather families experiences of SEND provisions and suggestions on how SEND services might be improved. | Survey emailed to Derbyshire Parent/Carer Voice's members, posted on Derbyshire SEND LO, promoted in social media platforms (Facebook, twitter, Instagram) | About half of families did not feel they were supported by care professionals or had regular updates about their children care. Only 10% of parents used the local offer to find oiut about care advice. Familes felt that communication about care planning and preparation for transitions was poor. Parents beleived that service access and timely support were the largest factors to improve current SEND support. | Low |
| McCaffrey, M & Robinson, P., 2020 | At least two methods | Parents/carers | Observational, Descriptive | LA | 320 families | Gather families experiences of SEND provisions and suggestions on how SEND services might be improved. | Letter from Chair of the SEND Improvement Group requesting co-operation from all partners across Education Health and Care in Tameside to promote the survey link to families within their services, lead people informed parents/carers about the survey and signposted them to it place on the LO landing page in Tameside and Derbyshire, survey promoted through social media platforms (Facebook , twitter, Instagram) | Over half of families felt they were supported by care professionals and had regular updates about their childs care. Parents beleived quality time was the largest factor that would improve family wellbeing. Most families beleived the support provided was not timely. 1/3 families said they were not involved in the EHCP process due to lack of vcommunication. Active parental involvement and improved communication were desired by parents. Parents beleived that early planning with the young person was the best preparation method for the transition to adulthood. Timely assessment and improved perso centered planning were marked as the best ways to improve immediate support. | Low |
| Stevens, J., 2020 | At least two methods | Parents/carers | Observational, Descriptive | LA | 100 PCs | Feedback on services and providers and to help understand what is important to respondents | Not stated | Over 60% of children took at least 6 months to be assessed for SEND. Half of famlies have trouble with trips and social occasions. 30% of parents often neglect themselves to care for their SEND child, with 80% report issues with emotional wellbeing. The vast majority of parents feel their child's needs are not being met. Half of parents found the EHCP process difficult. Less than half of parents had used the local offer, and half of those found it easy to use. | Low |
| Bristol City Council , 2021 | Quantitative (primary) | CYP and parents/carers | Observational, Descriptive | LA | 200 PC's | Gain insight into PC experience of SEND support for their children. | Survey promoted through the LO, schools and education settings and the Council and Health partners parent carer networks and social media platforms | >50% of parents felt their childs needs were mostly met. Most parents felt that extta educational support was not sufficient. Children with an EHCP plan were more likely to exerience an exclusion. 1/5 felt that their child had been left out frequently. Most respondents rated the EHCP process as at least satisfactory. Most respondents were not aware of the Local offer website. | Low |
| Bristol City Council, Bristol Parent Carers and Supportive Parents, Bristol Learning City, NHS Bristol, North Somerset and South Gloucestershire CCG., 2020 | At least two methods | CYP and parents/carers | Observational, Descriptive | LA | 370 PC's | To summarise PC and CYP experiences of SEND services and provision in Bristol. Also, compare to Local Area leaders’ responses. working together and with parents and carers and young people to improve SEND services and provision | No methods reported, though the authors note: There are 10,051 children with an identified special educational need or disability in Bristol’s maintained nurseries and schools, so we are mindful that this [370 respondents] is a very small percentage of the number of families who have a child with SEND – about 3.6% - but it is a helpful start in providing partners with some insight into parents’ experiences and views | Most respondents found it difficult to apply for an EHC assessment. Many respondents felt their childs development suffered due to a delay in recognising their childs needs. Most parents rated the EHC plan as satisfactory or better. Nearly half of EHC plans had not been reviewed in the past year. Mainstream schools were found to be worse at meeting SEND childrens needs than specialist schools. Parents gave mixed responses on their childrens support outside of the classroom. The majority felt their children were bullied by other children and 30% felt that staff may have bullied them. The majority found the transitions into post 16 education and into adulthood very difficult. | Low |
| Families And Carers Together in Buckinghamshire and Buckinghamshire SEND Information, Advice and Support Service, 2021 | At least two methods | Parents/carers | Observational, Descriptive | LA | 530 PC's | Summarise families' experience with SEN provisions in Buckinghamshire. | Methods section largely missing. Two pieces of methods info were: 1) survey eligibility (parent/carer of a child with SEND 0-25) and 2) that the survey was available to complete for three weeks between the 1 st and 22nd of March, 2021. Also that the LA were Not involved in developing and editing the report (though there is a conflict of interest) | The SENCos were highly regarded, however SEND support was viewed as poor. EHC plans were vague and the LO was rarely accessed. <44% felt that their child was receiving adequate support. | Low |
| Dodsworth, S. (Service for SEND for Lincolnshire County Council), 2021 | Qualitative | Mixed stakeholders | Observational, Descriptive | LA | 29 meetings, <300 email responses | To assess the current SEND provisions and barriers to family services. To assess if current improvement plans will address the appropriate SEND provision issues and adjust accordingly. | Dedicated email account set up for written submissions, virtual meetings | There is inconsistent and minimal communication with families when decisions are made about their childs care. Parents expressed a lack of timeliness in response to their enquires. EHCPs were often poorly written and non-person centered. There has been an increasing demand in specialist placements as mainstream schools are struggling to meet the needs of SEND CYP. There is a lack of integration between social care and family services which is impacting care for CYP. | Low |
| Torbay Council , 2018 | At least two methods | Education professionals | Observational, Descriptive | LA | 23 SENCos | Feedback from SENCOs to drive service developments | Survey sent to Primary, secondary and special schools SENCos | Practitioners stated that multi-agency involvement would greatly improve the annual review process. The majority of practitioners felt supported by the SEN team. EHCPs can be written more clearly and are better with PC and CYP involvement. Information is often duplicated across paperwork. | Low |
| Scambler, M. , 2021 | At least two methods | Mixed stakeholders | Observational, Descriptive | LA | 62 professionals, 725 PC's, 889 CYP | Establish the impact of legislation changes on CYP with SEND. Use the data to look for any emergency changes needed to SEN provisions. | Stakeholders from different organisations including East Sussex County Council, East Sussex Clinical Commissioning Group, East Sussex Healthcare NHS Trust, Kent Community Health NHS Foundation Trust, Sussex Partnership NHS Foundation Trust and several local schools. Survey sent to parent carers by multiple channels including ESPCF and Amaze, ESCC social media, ISEND social media, SENCO hubs, Parent GoverNors, Virtual School Bags, GP’s, and Community Paediatrics. | Many parents found accessing SEND related services difficult. There are inconsistent SEND provisions across schools which may be linked to inconsistent joint working.Large waiting times, strict referral criteria, and lack of capacity are preventing many with SEND needs from being supported. | Low |
| Northumberland County Council, Northumberland Parent Carer Forum, Northumberland Clinical Commissioning Group, 2020 | At least two methods | Parents/carers | Observational, Descriptive | LA | 426 PC's | Understand what happened to families with SEND during lockdown and inform SEND provision planning in Northumberland. | Survey promoted through social media channels, direct contact with SEND groups within Northumberland, Northumberland Parent Carer Forum and professionals across education, health and social care. | Lockdown negatively affected SEND childrens mental health significantly more than their physical health. Lockdown helped SEND children spend more time with their families and feel less stress from school. Services were greatly reduced and/or paused during lockdown. The majority of health or social care assessments were dalayed or cancelled. Only 40% of SEND children received an EHCP. Most families felt they needed more home schooling. | Low |
| Milton Keynes Council, 2017 | Quantitative (primary) | Education professionals | Observational, Descriptive | LA | 36 schools | Understand the impact of the SEND reforms and progress being made locally | Milton Keynes SEND service teams sent a survey to all mainstream schools | Most schools had an increase in SEND children with the new reforms. The majority of schools used assessments to determine SEND childrens progress. More thsn half of schools sometimes do not incluce CYP in their reviews. Schools expressed some confusion on their role in the EHC space, with the main ways to help being more staff training and consistent processes. | Low |
| Cambridgeshire County Council , 2016 | At least two methods | Parents/carers | Observational, Descriptive | LA | 167 PC's | Get parents feedback on the new SEND system and a bench mark for future years to judge progress against | Methods only partial, and state that 4 events were held, followed by an online survey. Thematic analysis of data. | About a 1/3 of parents felt the support their child received was poor. Only half of parents had used the local offer. The majority of parents felt they were not well involved in decisions relating to their childs education. More than half of parents did not find the information the needed in the local offer. | Low |
| Cheshire East Council , 2019 | At least two methods | Parents/carers | Observational, Descriptive | LA | 123 PC's | Gather the LA's views of current SEND service provisions to help commission superior services in the future. | Consultation promoted through: - The CWD database (approximately 300 members on record) - The Parent Carers Forum - The Cheshire East council website | 77% had accessed short break services within the last 24 months. Many local short break services had not been accessed. The Cheshire East CWD Team and Social Media were the most used places to find information about short breaks, with a lesser 12% finding information through the Local offer. The primary reason for short breaks not being accessed was families lack of awareness. The most important factors in accessing short breaks was the staffs awareness of needs and the location of time of the activities. | Low |
| Cambridgeshire County Council , 2018 | At least two methods | Parents/carers | Observational, Descriptive | LA | 75 PC | Assess if the Cambridgshire SEND support effectively meets PCs needs. | Parents and carers of children supported by: -Disabled Children’s Early Help Team -Disabled Children’s Social Work Teams North & south - Disabled Children’s Social Work Units North & south | The majority of families and carers felt listened to and involved in the development of the EHCP plan. The majority of parents felt that there was increased child independence, which helped the parents spend time doing other activities. A minority said that social care was involved in the EHCP process. | Low |
| Enfield Council, 2022 | At least two methods | CYP | Observational, Descriptive | LA | 82 CYP | Gather CYP views about play and leisure opportunities available in Enfield. | On-line survey circulated to All About Us Network | girls enjoyed the same activities and wished to do similar activities in the future. Girls were more interested than boys in getting involved with the scouts. The children wanted to find out about the activties through schools and their parents. | Low |
| London Borough of Richmond upon Thames, 2018 | At least two methods | CYP | Observational, Descriptive | LA & MAT | 24 CYP FG's, 25 CYP questionnaire | Engage with CYP with SEND to consider how to provide high quality SEND provisions on a budget. | Focus groups and quesitonnaires in special and mainstream schools to ensure pupils with EHCPs and those receiving SEN Support were included | The majority of respondents wanted an improved post-16 offer. Few young people had contributed to their own EHCP plans. Respondents supported the investment into annual reviews. Lack of investment from the central government had reduced therapeutic provisions and learning support assistant availability. | Low |
| Wiltshire Parent Carer Council, 2018 | Qualitative | Parents/carers | Observational, Descriptive | LA & MAT | 106 PCs | Consider the current and future challenges facing SEND provisions in Wiltshire schools. | Discussion groups in Salisbury, Trowbridge and Chippenham for parent carers of CYP with SEND held by Wiltshire Parent Carer Council | Early intervention and timely support for SEND were recognised as vital for reducing special school places for some children. These can also reduce the number of exclusions and reduiced the number of families needing to home educate. Poorly written EHCPs and lack of staff training were identified as serious issues in SEND care. | Low |
| Norfolk County Council, SEND Norfolk and Norfolk and Waveney Clinical Commissioning Group, 2020 | At least two methods | CYP | Observational, Descriptive | LA | 133 CYP | Sought the views of young people | Survey sent to number of both statutory and voluntary organisations, schools and colleges in Norfolk, available on the service web-site young person’s page, advertised on social media platforms. | Half of CYP felt they were involved in their reviews. More than half of children felt they were listened too and understood. Just under half of CYP did not know where to access information regarding support for SEND. | Low |
| **SEND identification and provision: National govt / DfE led/commissioned research** | | | | | | | | | |
| House of Commons Education Committee, 2019 | At least two methods | Mixed stakeholders | Observational, Descriptive | LA & MAT | 700 testimonials, 12 group interviews with mixed stakeholders. | Assess the impact of Part 3 of the Children and Families Act 2014 on families with SEND. | Inquiry launched on 18 April 2018. 700 written submissions, including from parents/carers and other stakeholders. 12 oral evidence sessions from parents, children and young people, representatives from charitable organisations, schools, colleges, local authorities and the health sector and the Minister of State for Care, the Minister of State for School Standards and the then Parliamentary Under-Secretary of State for Children and Families. Also used existing literature and policies to arrive at conclusions. | Inadequate funding is a major factor in schools and LAs failing to meet needs of CYP with SEND. There is an absense of accountability across the DfE and LAs. More guidance needs to be provided for schools on supporting SEN children. Ofsted and CQC need the funding to carry out more rigourous EHCP and LA inspections. | Low |
| Griggs, J & Bussard, L., 2017 | At least two methods | Parents/carers and SEND/education professionals | Observational, Descriptive | LA | 16 CYP and 16 Providers. | Assess the quality of early years settings in meeting the needs of children with SEND and their families. To assess the facilitators and barriers to SEND provisions across different SEND severities. | Case study children were selected from the families who completed the age three interview as part of the SEED longitudinal survey, Furthermore, there was No direct question in the SEED survey about whether the child had been formally identified as having SEND. Therefore, the qualitative study used a proxy measure of SEND, drawing on the following information collected in the survey: 1. Parents reported that their child had a disability/ health condition, and/or had a developmental or behavioural problem; 2. Children had a score more than one standard deviation below the mean on the British Ability Scales (BAS) III naming Vocabulary scale, as measured in the survey; or children were not able to complete the naming Vocabulary module due to their disability/health condition  Fieldwork took place between October 2015 and February 2016. Two topic guides were used to guide the discussions; one for parents and another for providers.   Interviews with parents lasted approximately 40 minutes on average, those with early years providers tended to be longer (approximately an hour), reflecting the more detailed topic guide.  Interviews were conducted face-to-face with both parents and providers, with the exception of one parental interview, which was conducted over the telephone.  Initial contact was made by an introductory letter to the parents, which included further information about the study. This was followed up by a telephone call to explain the research in further detail, confirm selection criteria and invite participation.   Once parental consent had been secured invitation letters were sent to providers, who were also contacted by telephone to invite them to participate. Flexible interview dates and times were offered to maximise participation.  In all but two cases the interviews took place on the same day, and all bar one included a short observation pf the child in the setting. All parents who took part received a £20 high street shopping voucher.   The qualitative data were analysed using Framework - an approach to qualitative data management which is systematic and comprehensive. | Proximity to the parents’ house was the predominant factor about where to send their child for education and care. Those with more complicated SEND consider more factors. Some parents did not engage with setting activities as a result of parents not feeling its necessary to get involved in their child’s early years care. Short breaks providers felt happy about advertising the EHCP process. However, they felt concerned about the volume of paperwork required. There was variation in the families’ experiences of SEND services depending on SEND of their child and across locations. The early years provisions benefits were confidence, improved social skills, and condition improvement. | Medium |
| Parish, N & Bryant B., 2015 | At least two methods | Mixed stakeholders | Observational, Descriptive | LA | 13 LA's, 19 stakeholders interviews. | To collate and analyse data on the funding and costs of SEND educational provisions. Use the data to inform funding policy for students with SEN. | Literature review and data analysis, and fieldwork (November 2014 to March 2015): semi-structured telephone interviews to develop question frameworks for local level stakeholders, investigating with interviews, survey, and exemplar profiles of CYP with SEN: 1: Identifying need – how do LAs predict and analyse the incidence of SEN to plan and distribute funding? 2: Allocating funding – how do LAs decide the distribution of funding for pupils supported by SEN provision? 3: Achieving outcomes – how well do the different approaches to funding for SEN used by local areas meet the needs of pupils and parents? Do particular approaches to funding contribute to better outcomes for children and young people with SEN? 4: Developing options for a future SEN funding system. | The current spend on YP with high needs does not consider factors such as deprivation, prior attainment and disability fairly. Highly incusive and smaller schools may not be able to match the £6,000 support for SEND pupils. Joint commisioning of authorities may be the answer to providing support to children with complex SEND needs. However, effective joint commisioning was rarely seen. | Low |
| Department for Education, 2021 | Qualitative | Education professionals | Observational, Descriptive | LA & MAT | 30 teacher interviews and 30 school leader interviews | To understand schools approaches to identifying pupils who need SEN support, meeting those needs and evaluating whether the support provided is adequate. | 60 45 minute qualitative interviews with topic guide, with leaders and teachers from mainstream primary and secondary schools in England, quota sampling for equal split of respondent types and school stage, and a range of characteristics, recruited via DfE Panel Study of Leaders and Teachers. Interviews via telephone and video-conferencing platforms. Field work March-May 2021. Topic guide explored leaders’ and teachers’ experiences with pupils receiving SEN support (i.e., excluding pupils with an EHCP) in pre-pandemic period. Analysis using framework/grid of discrete topic areas, and comparisions by interview, respondent group & school type. Unique themes could also develop. Researchers established most and least common themes in each topic area. | Schools used SENCO assessments, teacher observations and baseline testing to identify SEND pupils. The primary barriers to identification were classroom resources and access to SEN specialists. Having an active SENCO who communicated school staffs' responsibilities ensured the best support. Use of SEN specialists to support teachers also provided the best support. | Low |
| **SEND identification and provision: Third sector-led research** | | | | | | | | | |
| Amaze, 2020 | Qualitative | Mixed stakeholders | Observational, Descriptive | LA | 37 parent carers (focus groups), 63 parent carers (survey), 25 professionals (interviews) | Find out how participation and co-production can be strengthened in the borough so that parent carer voices, and those of CYP, shape and inform the design, delivery and review of services to meet the needs of the local SEND community. | Intention to reach a representative cross-section of parent carers whose childen come from all age groups and have different types of SEND, families with children in mainstream and special education, home or further education, families with children with SEN Support, EHCP and families with children who may have SEN but are still awaiting a diagnosis. Telephone interviews with professionals to increase response rate, headteachers and SENCOs invited from a number of schools and senior staff at a number of key voluntary organisations. Flyers advertising our focus groups and survey distributed to education settings, health, social care and other organisations by LA. Support groups and voluntary organisations approached to circulate review. Social media channels used to publicise review. Education settings, children's centres, the child development clinic at the Whittington, health visitors and Portage approached. Support groups publicised review on social media pages, websites and at face-to-face meetings. | More than 60% parents said they did not feel involved in planning services for their children. Parents felt that communication from SEND professionals was poor and that they did not feel listened to. Parents also recognised the lack of joint working across care those involved in their childrens care. A small minority of parents would use a professional or local offer to find information for SEND support. More than 70% of families wanted access to more SEND information. | Low |
| Essex County council et al., 2016, 2018, 2019, 2020 | Quantitative (primary) | Mixed stakeholders | Observational, Analytical | LA | 782 CYP, 3899 PC, 833 EHC practitioners questionnaires. | Survey professionals and PCs of SEND CYP to assess the ability of Hackney to meet SEND needs. Combine survey data with other studies to form a SEND provision improvement plan. | Surveys available online or as a paper format. No information available on how participants were identified and recruited. | A higher percentage of practioners compared to PC and CYP felt that inclusion in planning, progress to outcomes and families views were part of the care for children with SEN. Hackney - 3/4 of practicioners felt that EHCPs helped build more person centered care and was helpful to the young people. Knowlsley - 3/4 of parents said the support received was at least good. Parents scored childrens outcomes at less than 50%, and parental outcomes at just above 50%. Over half of practicioners agreed that EHCPs had helped them in all areas of work. About 50% of practicioners beleived the EHCP plan was helpful for SEND CYP. Southend-on-sea - On average, 44% of parents ranked the support their child received as poor. Most parents said that both their own and childs views were at least partially listend to. Most parents said their children had not yet achieved all their goals set out in yheir EHCP. Most parents agreed that QOL had not improved with the addition of EHCPs. Practicioners felt that PCs and CYPs views were mostly included. 2/3 of practicioners felt that EHCPs had helped children with SEND. Most practicioners felt that SEND children were progressing towards goals. WEST BERKSHIRE - most children felt their own and their families views were at least mostly included in planning. Over half of children felt that recent support was helpful to quality of life and reflected their needs. Over 2/3s of practicioners felt that recent support had improved CYPs quality of life. The vast majority of practicioners felt that childrebs views were included in planning. More than half of practicioners felt SEND CYP had reached their goals. More than half of parents regarded the support their child received as good. Most parents felt their views were mostly reflected in their childs planning. Parents were mostly satisfied with their childrens outcomes. Parents had mixed views on their personal outcomes. Just over half of practicioners felt EHCPs had at least mostly supported SEND CYP. Over half of parents rated the quality of life improvements and support their child received as at least good. Over half of parents said that EHCPs had improved their personal QOL. | Low |
| Healthwatch Swindon, 2020 | At least two methods | Parents/carers | Observational, Descriptive | LA | 102 PCs | To gather feedback on SEND services in Swindon. | Surveys completed online by people involved with Swindon SEND Families Voice | People do not limit themselves to one way of making appointments with GP, telephone most frequently used. More people found difficult rather than easy to make an appointment (waiting times, availability of appointments, emergency appointments given when required). Hospital, dentist and community health services most common health services used in last 12 months. Mixed responses of health services use. Fewer participants receive social care support than health services. Majority of participants feel safe in local community. Broad understanding of "safeguarding" and where to report a concern. Improvements regarding speed of response to telephone calls, shorter waiting times, more support, more coordination between services and more funding Concern about transition from children’s to adult services. Family or friend and Swindon LO most common sources of information. | Low |
| PACTS , 2021 | At least two methods | Parents/carers | Observational, Descriptive | LA | 72 PCs | Feedback on about SEND provisions across multiple settings during and after the pandemic. | Survey advertised via PACTS website, email to members, PACTS Facebook open page, Twitter, Instagram, Stockport’s LO, Council’s SEND Facebook page, NHS Facebook groups, shared via parent groups also shared it. | Over 70% of post-17 children have a SEN support plan or EHCP. Educational support for SEND children over lockdown became increasingly worse in higher age groups. The majority of parents of 5-11 year olds felt the social care services were less than okay during lockdown. All age groups felt that social care services were less than okay following the lockdown. There were mixed opinions on the quality of heath and educational support following lockdown. Most parents have used the local offer before. | Low |
| Parish N, Bryant B & Swords B., 2018 | At least two methods | Mixed stakeholders | Observational, Descriptive | LA | 93 LA surveys and 9 LA investigations | To quantify SEND expenditure for CYP with SEND. To understand why SEND spending has risen and propose actions to alleviate the heavy expenditure. | Scoping review, survey, fieldwork | Increased total spend for high needs block and high needs block allocation; Increased gap between high needs expenditure and funding; Increased average expenditure per LA; Increased spend due to increased number of CYP needing support and increased units costs of placements. | Low |
| The National Autistic Society, 2015 | At least two methods | CYP and parents/carers | Observational, Analytical | LA | 1,431 PC's and 231 CYP. | To understand the current experiences of autistic CYP and their families in education compared with previous research. | Survey of those who have and have Not been through the new system | The vast majority of parents of SEND children felt that provision for education, mental health and social care had not improved with the introduction of the CFA in 2014. The vast majority of families felt they did not receive enough support for buddying, daily skills, short breaks or mental health support. The majority also felt that there was a lack of timely support which drastically effected their childrens educational attainment and social skills. A minority of parents felt satisfied with their EHC plan and had accessed the local offer. | Low |
| Sheffield Parent Carer Forum, 2019 | At least two methods | Parents/carers | Observational, Descriptive | LA | 706 PC's | Understand how SEND affects a family and issues parents have with SEND provisions. Assess the impact of the Children and Families Act 2014 on parental satisfaction. | Survey advertised via SPCF’s email list, SMS list, website and social media channels; survey circulated to the email list of the Child Disability Register; survey publicised by parent support groups to their members; targeted emails sent to parents of children with primary needs other than ASD, parents of children aged 0-5 years and 16-25 years, and Non White British parents; local BME organisations, Family Centres, special schools with predominantly Non-ASD cohorts and further education colleges asked to publicise the survey | More families have felt increasingly isolated. The majority of parents feel their children are getting too little input into decisions and less support from services. Parents reported great difficulty accessing mental health services, where the majority of families were affected by mental health issues. Parents expressed difficulty applying for EHCPs, and were concerned about the quality of the implementation. Only a quarter of parents felt their SEND childrens needs were being met in mainstream schools. | Low |
| British Association of Social Workers, 2018 | Quantitative (primary) | Education professionals | Observational, Analytical | LA | Not stated | Collect the views of school leaders on their experiences with SEND children. Use the data to as evidence for funding shortages for SEND children. | Sought views of school leaders on experiences with SEND in relation to funding | 94% of school leaders are finding it increasingly harder to find the funding to support SEND children. More than two thirds of school leaders stated that EHCPs do not accurately reflect and address the needs of SEND pupils. | Low |
| Association of Directors of Children's Services, 2017 | At least two methods | Directors of Children's services | Observational, Descriptive | LA | Sample of 112 LAs | Gather information on home to school transport spend in 2015/16 | The authors contacted all Directors of Children's Services in October 2016, requesting home to school transport spend for 2015/16. 112 LAs responded about the following outcomes: amount spent, key issues driving demand, and local costs. Mixture of quantitative methods (e.g. expenditure data) and thematic assessment of drivers of demand. | Variations in expenditure for SEND based on type of LA (% highest in London Boroughs); Issues driving increased demand and costs of home to school transport: increased number of CYP with SEND, shortage of mainstream school places and specialist educational provision, geographical nature of LAs, differences in start and finish times for post-16 provision, lack of capacity in transport market. | Low |
| Swords B, Parish N & Kulawik K., 2019 | At least two methods | Mixed stakeholders | Observational, Descriptive | LA | 45 LA surveys, 8 LA investigations | Understand factors driving demand and spend on home-to-school transport over the previous 5 years and what LAs have done to mitigate overspending. | Scoping review, survey, fieldwork | Significant variation between LAs on expenditure on home-to-school transport; Higher expenditure in rural areas; Increased number of children with SEND in receipt of transport; Increased expenditure on transport for children with SEND due to increased number of children with EHCPs, increased complexity of needs, increased distance travelled | Low |
| **Stakeholder strategy/planning** | | | | | | | | | |
| North Yorkshire County Council, Council for Disabled Children, North Yorkshire Clinical Commissioning Group, 2021 | Qualitative | Mixed stakeholders | Observational, Descriptive | LA | 48 parents/carers, 44 professionals | To obtain parents/carers' and professionals' views to inform the development of the SEND strategy | A series of focus groups were analysed thematically, to identify common themes, and differences by location and respondent type | Key themes relevant to LAs: Constituents of a 'good life' for CYP with SEND included being valued, with good mental health and emotional wellbeing, enjoying a wide range of opportunities and challenges, celebrating achievements, becoming more independent, and being safe. Current obstacles to this were: strategic (insufficient accountability and transparency, lack of joined up working, poor communication and engagement with parents/carers); operational (insufficient local provision, particularly social, poor experiences of the EHCP process, services not needs led, ineffective 'early help', heavy burden on parents/carer to coordinate support, overstretched, understaffed workforce; insufficient respite. Priorities to improve locally included: improved attitudes towards CYP and families; clearer, more accessible information, and transparent communication, needs-led services, ending the post-code lottery, more social opportunities and respite. | Medium |
| Council for Disabled Children, 2021 | At least two methods | Mixed stakeholders | Observational, Descriptive | LA | Focus groups with 40 CYP, 9 parents/carers, and a survey of 43 parents/carers | To develop the SEND strategy 2022-2027 by asking CYP with SEND what they thought was good about support in Rochdale and what could be improved. Parents/carers of children with SEND were asked what Rochdale could do differently to better meet the needs of their children. | Parent carers views gathered through focus groups and survey to reach a diverse range of parent carers. | **CYP** valued the activities and groups, and the variety. Some CYP wanted a wider choice, including “outside sports” and ‘mainstream’ groups. Some CYP wanted improved transport (buses and taxis) and better wheelchair accessibility. Some CYP wanted better preparation for adulthood, including information about services and jobs, as well as bringing back previously valued support and ensuring services were affordable. Parents/carers compared leaving school to a 'cliff edge' and wanted better preparation for adulthood, and employment support. They also reported there was little support available to help coordinate all the different service inputs and wanted a single key worker to help, as well as improved information sharing to minimise the need to repeat their story, and to ensure professionals were using the most up to date information about their children. | Low |
| Wolverhampton Council, 2019 | At least two methods | Mixed stakeholders | Observational, Descriptive | LA | 178 CYP (mixed sex and ethnicity, ~50% had a disability affecting daily activities, expected to last at least 1 year), 89 parents/carers, 62 professionals (nearly half education, one quarter health, 10% care, 10% voluntary sector) | To inform strategic, system-wide priorities, commissioning, and future plans, to provide evidence-based support services tailored to need, and to improve independence and outcomes for CYP with SEND, and their families, in Wolverhampton, with the lens of independence and inclusion, reducing 'social and environmental barriers to living an ordinary life' | Online surveys hosted and promoted on the CWC Consultation Hub: CYP survey, parent/carer Voice4Parents annual survey, professionals' survey, all with with questions about 'a good ordinary life' and preparing for adulthood. Thematic analysis by respondent group | **CYP:** generally positive about future aspirations, rich and varied depiction of good ordinary life: fun activities, functionality, daily activities, Independence; **Professionals:** focused on inclusion, equality and access to services and support, and communities, relationships and leisure. Professionals said more could be done to achieve good ordinary lives, with more coordinated tailored support, as early as possible to prepare them; **Parents/carers:** more support, and better functioning services in Wolverhampton would help families, and would not require repeating their story many times (78% said this), the leisure offer was unsatisfactory for three quarters of the p/c sample, information about available services was difficult to find (73% said this), with low awareness of the Local Offer(66% had not heard of it), >50% said services in the city do not work together to support families. | Low |
| Westminster City Council, 2017 | At least two methods | Mixed stakeholders | Observational, Analytical | LA | Not stated | To inform the development of the joint LA and Clinical Commissioning Group SEND Strategy for Westminster area using data about complex needs in the borough and nationally, current service provision, identify gaps in services and areas of unmet need | Two workshops, focus groups, interviews and local data from key stakeholders and providers. | Key messages: 1) Early identification and support: ASD diagnosis, support and information could be more transparent and tailored; 2) Post-diagnosis support requires clear, accessible information about available services, how to access them, who is involved in autism pathway, autism friendly local offer. Continued strategic and operational engagement between LA, schools, CCG and health partners; 3) Local offer should be updated in consultation with CYP with autism and their families, all professionals should be aware and signposting families to the LO; 4) Aim for 'seamless transfer' of service provision when children exit or arrive in the borough; 5) Ensure service planning, funding, workforce development and capacity considers projected growth in the cohorts with SEND; 5) Enhance post-16 information to include more information about preparing for adult life; 6) Consider pathways for specific cohorts: high functioning autism, complex needs and requiring medical interventions, SLCN, PMLD. 7) Local strategies to consider how to promote mental health and wellbeing of children and young people with SEN with early intervention and prevention. 8) Comprehensive, combined SEND database, across sectors, would help understand local needs, aid future planning and communication about post-16 education; employment; supported/independent living and accessing the local community. | Low |
| **Transition to adulthood** | | | | | | | | | |
| Jones, DfE, 2018 | Qualitative | Mixed stakeholders | Observational, Descriptive | LA | n=70 (30 apprenticeship providers, 28 employers, 9 third party organisations) | To provide an evidence base for the effectiveness of apprenticeship funding support for people with LDDs and/or from disadvantaged backgrounds, understand how apprenticeship providers and employers support apprentices, how this support is resourced and funded, and whether it enables individuals to succeed in and complete their apprenticeship. | Indepth interviews (face-to-face or by phone, 60-90 minutes) with key stakeholders providing support to apprentices with LDD and/or from disadvantaged backgrounds. Audio-recordings were transcribed and analysed using a thematic framework approach. | 1) Providers may ask for apprentices to provide/obtain an EHCP at any point during the apprenticeship to identify learning support needs, but EHCPs were harder to secure with LAs reluctant "to write apprenticeships into an individual’s plan" because of funding concerns; others said apprentices without EHCPs were ineligible for some programmes. 2) Partner-led recruitment (which can include LAs) was the least commonly used method (compared to employer or provider led) but was viewed positively as it can target programmes to apprentices from particular backgrounds, e.g. individuals with LDDs, and improve apprenticeship access. Partner-led routes also meant providers were "more likely to be aware of the wider support needs of the apprentices", partially avoiding dependence that people self-declare additional needs. 3) Charities can be approached with a Section 106 (legal agreements between LA and developers) meaning they must accept a specific quota and 'type' of young people from an area, but the funding was too 'contract driven', in-flexible about training and led to inadequate support for this cohort. 4) Funding: Some apprentices have undiagnosed LDDs or learning support needs not related to LDD for which employers would like to apply for further Additional Learning Support funding, but criteria, eligibility and restrictions on use of funds are unclear. Many apprentices have mental health issues, which impact on learning and safeguarding support needs, but again providers are uncertain about eligibility and evidence needed to get additional learning support funding. | Low |
| Natspec, 2021 | Quantitative (primary) | CYP and parents/carers | Observational, Analytical | LA | 137 parents/carers of young people with SEND in England and Wales | To discover how young people with EHCPs or Welsh equivalent plans and their families find out about post-16 options, views on the amount, quality and timeliness of information, advice and guidance they receive, if parental satisfaction with these aspects differs between England and Wales, and to identify examples of effective and poor practice. | Online survey of parents/carers of young people with SEND (two-thirds from England, one-third from Wales). Most young people were educated in special schools or specialist further education colleges, 55% had autism, plus/minus a range of other SEND types. Survey items included open and closed questions.No information about sampling or recruitment. | Most parents/carers said LA information, advice and guidance (IAG) services on post-16 options was not working well (though greater satisfaction in Wales), with a call for: 1) earlier IAG to allow informed decision-making and aid transitions; 2) the full range of options, delivered impartially (not selected by LAs in advance, no witholding of information, and not steering people on the basis of cost); 3) weight given to young people's and families preferences, and involvement of young people in decision-making; 4) access to a properly funded service, where a named, trained person with good SEMD understanding can help young people, parents/carers understand the different options over a period of time; 5) clear information and timelines about post-16 options and choices from Year 9, referenced at every annual review; 6) improved local offers, with one point of information including all post-16 education and training options. co-created with users so it is comprehensive, searchable, and navigable. | Low |

## Online resource 11. References: Grey literature studies

1. Adams, L., Tindle,A., Basran,S., Dobie,S., Thomson,D., Robinson,D., Codina,G. (2018). *Education, Health and Care plans: A qualitative investigation into service user experiences of the planning process*. https://assets.publishing.service.gov.uk/ Retrieved from https://assets.publishing.service.gov.uk/media/5aba6920e5274a1aa2d418c3/Education_Health_and_Care_plans_-_a_qualitative_investigation.pdf
2. Adams, L., Tindle,A., Basran,S., Dobie,S., Thomson,D., Robinson,D., Shepherd,C. (2017). *Experiences of Education, Health and Care plans: a survey of parents and young people*. https://assets.publishing.service.gov.uk/government/uploads/system/uploads/attachment_data/file/709743/Experiences_of_EHC_plans_-_A_survey_of_parents_and_young_people.pdf
3. Amaze. (2020). *Improving parent carer participation and co-production in Haringey*. https://www.haringey.gov.uk/sites/haringeygovuk/files/amaze_report4.pdf
4. Association of Directors of Children's Services. (2017). *Home to School Transport: Survey of Local Authority Spend 2015/16*. https://adcs.org.uk/assets/documentation/Home-to-school_transport_ADCS_policy_position_and_cost_analysis_final_161213.pdf
5. Atkinson, E. (2018). *Blackburn with Darwen Council High Needs Provision (SEND) Review*. https://search3.openobjects.com/mediamanager/blackburn/directory/files/bwd_report_final_published.pdf
6. Booth, D. H., E. Jones,G. Richardson,S. Robinson,G. Sumner,H. Waddington,C. . (2019). *Special educational needs and disabilities in Lancashire: A joint strategic needs assessment*. https://www.lancashire.gov.uk/media/912472/special-educational-needs-and-disabilities-in-lancashire-2018-19.pdf
7. Bournemouth Christchurch & Poole Council. (2021). *SEND Survey 2021*. Retrieved 29/05/24 from https://haveyoursay.bcpcouncil.gov.uk/send-survey-2021
8. Bournmouth Christchurch and Poole Council. (2021). Local Offer Survey Responses (Social care section). In (pp. 6). https://www.bcpfamilyinformationdirectory.com: Bournmouth, Christchurch and Poole Council.
9. Bradford City Council. (2020). *CYP outcomes* (Bradford's Special Educational Needs and Disabilities (SEND) Local Offer Annual Report (2019-2020), Issue. https://localoffer.bradford.gov.uk/. https://localoffer.bradford.gov.uk/public/images/images/1597138938.pdf
10. Bristol City Council. (2020a). *SEND Services Survey for Children and Young People Report*. https://www.bristol.gov.uk/files/documents/3627-send-services-children-and-young-people-survey-full-report-final/file
11. Bristol City Council. (2020b). *Survey of parents and carers of children and young people with Special Educational Needs and Disability (SEND)*. https://www.bristol.gov.uk/files/documents/3630-bristols-send-survey-report-spring-2020/file
12. Bristol Parent Carer Forum., S. P. S., Bristol City Council. . (2021). *Survey of Parents and Carers of Children and Young People with Special Educational Needs and Disabilities (SEND)*. https://www.bristol.gov.uk/files/documents/3605-parent-and-carer-consultation-report-2021/file
13. British Association of Social Workers., N. A. f. H. T. (2018). *Empty Promises: The crisis in supporting children with SEND*. Retrieved 29/05/24 from https://new.basw.co.uk/policy-and-practice/resources/empty-promises-crisis-supporting-children-send#:~:text=This%20report%20uses%20our%20findings,is%20now%20at%20crisis%20point.&text=Only%202%25%20of%20respondents%20said,statements%20for%20pupils%20with%20SEND.
14. Bryant, B., Parish,N., Kulawik,K. (2022). *Agreeing to disagree? Research into arrangements for avoiding disagreements and resolving disputes in the SEND system in England*. https://static1.squarespace.com/static/5ce55a5ad4c5c500016855ee/t/6221ee346c97bb4c0c754891/1646390841226/220222_LGA_SEND+disputes_report_FIN/AL.pdf
15. Cambridgeshire County Council. (2018). *Disabled Children’s Social Care: What Children and Young People told us about Disabled Childrens Social Work and Early Help Teams, April –September 2018*. https://www.cambridgeshire.gov.uk/asset-library/imported-assets/DCSC%20children%20and%20young%20people%20feedback%20summary%20April%20-%20Sept%202018.pdf
16. Cambridgeshire County Council., S. (2016). *Parents’ feedback on the new SEND system after one year*. https://www.cambridgeshire.gov.uk/asset-library/imported-assets/SENDIASS_parent_carer_feedback_report_June_2016.pdf
17. Cambridgeshire County Council., S. (2018). *Disabled Children’s Social Care-Parents and Carer Feedback Summary, April-September 2018*. https://www.cambridgeshire.gov.uk/asset-library/imported-assets/DCSC%20parent%20and%20carer%20feedback%20summary%20April%20-%20Sept%202018.pdf
18. Care Quality Commission, O. (2020). *COVID-19 series: briefing on special educational needs and disabilities provision, November 2020* (COVID-19 thematic series: November briefing December 2020, Issue. https://assets.publishing.service.gov.uk/media/5fd36f80d3bf7f3057adeb2f/COVID-19_series_briefing_on_special_educational_needs_and_disabilities_provision__November_2020.pdf
19. Cheshire East Council. (2019). *A summary of responses to Cheshire East Council’s Short Breaks for Disabled Children: Parent Carer Consultation*. https://www.cheshireeast.gov.uk/pdf/council-and-democracy/consultations/short-breaks-for-disabled-children-parent-carer-consultation-full-report.pdf
20. Cheshire East Council. (2021a). *Parent/carer views of the autism assessment process – January 2021 Survey results*. https://www.cheshireeast.gov.uk/pdf/council-and-democracy/consultations/consultation-results/parent-carer-views-of-the-autism-assessment-process-2021-final-report.pdf
21. Cheshire East Council. (2021b). *Parent/carer views of the Education, Health and Care needs assessment process – January 2021*. https://www.cheshireeast.gov.uk/pdf/council-and-democracy/consultations/consultation-results/parent-carer-views-of-the-ehc-assessment-process-2021-final-report.pdf
22. Child Friendly Leeds., L. C. C. (2021). Leeds Local Offer Website User Testing - March 2021. In. Leeds: Child Friendly Leeds. (Reprinted from: 28/11/2024).
23. Council for Disabled Children., R. B. C., Heywood Middleton and Rochdale CCG. (2021). *Raising Rochdale – supporting SEND children, young people and families*. https://search3.openobjects.com/mediamanager/rochdale/fsd/files/rochdale_send_alliance_strategy.pdf
24. Deacon, A. (2017). *Reading Early Years and Special Educational Needs and Disability (SEND) Consultation Report 2017- Consultation with Reading Early Years Providers*. https://consult.reading.gov.uk/children-education-early-help-services/childcare-survey-for-parents-carers-with-send-chil/results/final-consultation-with-settings-and-parentcarers.pdf
25. Department for Education. (2021). *SEN support: Findings from a qualitative study*. https://www.gov.uk/government/publications/special-educational-needs-sen-support-findings-from-a-qualitative-study
26. Devon County Council. (2018). *Parental Feedback from EHCP Statutory Assessments*. https://devoncc.sharepoint.com/sites/PublicDocs/Education/Children/Forms/AllItems.aspx?id=%2Fsites%2FPublicDocs%2FEducation%2FChildren%2FSEND%2FFeedback%20You%20Said%20We%20Did%2FEHCP%20feedback%20results%2FParental%20Feedback%20from%20EHCP%20Statutory%20Assessments%20Aug%2DNov%202018%2Epdf&parent=%2Fsites%2FPublicDocs%2FEducation%2FChildren%2FSEND%2FFeedback%20You%20Said%20We%20Did%2FEHCP%20feedback%20results&p=true&ga=1
27. Devon County Council. (2022). Your feedback on our short breaks offer. In. Devon: Devon County Council.
28. Dodsworth, S., Lincolnshire County Council,. (2021). *Suffolk County Council's Special Educational Needs and Disabilities Review Undertaken by Lincolnshire County Council July 2021 Review Team*. https://www.suffolklocaloffer.org.uk/asset-library/lincolnshire-independent-send-review-report-2021.pdf
29. Doncaster Borough Council. (2017). Local Offer – Tell us what you think to the new Local Offer survey results & Feedback from SENCo Cluster meeting. In. Doncaster: Doncaster Borough Council.
30. Enfield Council. (2022). *Results of CYP Play & Leisure Survey March 2022*. https://www.enfield.gov.uk/__data/assets/pdf_file/0014/24008/Results-of-CYP-Play-and-Leisure-Survey.pdf
31. Essex County Council., I. C., University of Lancaster. (2019). *Personal Outcome Evaluation Tool (POET): Survey Feedback 2018/19*. http://www.essexlocaloffer.org.uk/wp-content/uploads/2020/11/3a.-In-Controls-CYP-report-1.pdf
32. FACT Bucks., B. S. (2021). *Buckinghamshire SEND Survey 2021- Survey Report*. https://www.factbucks.org.uk/wp-content/uploads/2021/05/2021-Buckinghamshire-SEND-Survey-Report.pdf
33. Griggs, J., Bussard,L. (2017). *Study of Early Education and Development (SEED): Meeting the needs of children with special educational needs and disabilities in the early years* https://assets.publishing.service.gov.uk/media/5a7f0e5de5274a2e87db3857/SEED_Meeting_the_needs_of_children_with_SEND_in_the_early_years_-_RR554.pdf
34. Hackney Learning Trust. (2017). *SEND Provision in Hackney Provision Survey Findings August 2017*. https://search3.openobjects.com/mediamanager/hackney/fsd/files/send_provision_survey_results_report_v2_0_170928_2017.pdf
35. Hall, K., Leicestershire County Council. (2017). *Voice Work Report: Voices of Children and Young People with Special Education Needs and Disabilities and their Parent Carers*. https://www.leicestershire.gov.uk/sites/default/files/field/pdf/2020/10/1/send-voice-work-report.pdf
36. Hayter, S., Ofsted., Care Quality Commission. (2017). *Local Area SEND Inspection: Leeds City Council*. https://files.ofsted.gov.uk/v1/file/50000207
37. Healthwatch Swindon., S. F. V. (2020). *Swindon SEND Families Voice Survey Report*. https://www.healthwatchswindon.org.uk/sites/healthwatchswindon.org.uk/files/Swindon%20SEND%20Families%20Voice%20survey%20report%20VS%20EDITS.pdf
38. Herefordshire Council. (2018). Short Breaks Telephone Survey 2017-18 Findings. In (pp. 1-8). Herefordshire: Herefordshire Council.
39. Hodgson, L. (2021). The Derbyshire Local Offer Consultation Report Evidenced by Derbyshire Parent Carer Voice. In D. P. C. Voice (Ed.). Derbyshire: Derbyshire Parent Carer Voice.
40. House of Commons Education Committee. (2019). *Special educational needs and disabilities*. Publications.parliament.uk Retrieved from https://publications.parliament.uk/pa/cm201919/cmselect/cmeduc/20/20.pdf
41. House of Commons Select Committee. (2018). *Forgotten children: alternative provision and the scandal of ever increasing exclusions*. https://publications.parliament.uk/pa/cm201719/cmselect/cmeduc/342/342.pdf
42. House of Commons Women and Equalities Committee. (2020). *Unequal impact? Coronavirus, disability and access to services: full Report*. https://committees.parliament.uk/: House of Commons Retrieved from https://committees.parliament.uk/publications/4068/documents/40461/default/
43. Jones, E. D., E. (2018). *Exploring the funding and support for apprentices with additional support needs*. Department for Education Retrieved from https://assets.publishing.service.gov.uk/government/uploads/system/uploads/attachment_data/file/697649/Exploring_the_funding_and_support_for_apprentices_with_additional_support_needs.pdf
44. Keer, M. (2016). Not co-production: Was your child’s Education, Health and Care plan outsourced? https://www.specialneedsjungle.com/Not-coproduction-childs-education-health-care-plan-outsourced/
45. Lancashire County Council. (2017). FIND Newsletter Report from FIND Monitoring Questionnaire 2017. In. Lancashire: Lancashire County Council.
46. Lancashire County Council. (2021). *Education, health and care (EHC) plan annual review survey*. https://www.lancashire.gov.uk/children-education-families/special-educational-needs-and-disabilities/lancashire-send-partnership/publications/ehc-plan-annual-review-survey/
47. Leeds SENDIASS. (2021). SENDIASS Consultation March 2021. In. Leeds: Leeds SENDIASS.
48. London Borough of Richmond Upon Thames Children's and Schools Overview and Scrutiny Committee. (2018). *DEDICATED SCHOOLS GRANT: CONSULTATION ON FUNDING OPTIONS FOR THE HIGH NEEDS BLOCK*. https://5f2fe3253cd1dfa0d089-bf8b2cdb6a1dc2999fecbc372702016c.ssl.cf3.rackcdn.com/uploads/ckeditor/attachments/4220/High_Needs_Block_Dedicated_Schools_Grant.pdf
49. MacDonald, C. (2021). Northumberland Short Break Review Report Version 1.1 February 2021. In. Northumberland: Northumberland County Council.
50. McCaffrey, M., Robinson,P., & Tameside Metropolitan Borough Council. (2020a). *Glossop in conversation with families about SEND*. https://www.tameside.gov.uk/TamesideMBC/media/sen/Appendix-4-Glossop-survey-report.pdf
51. McCaffrey, M., Robinson,P., & Tameside Metropolitan Borough Council. (2020b). *Tameside in conversation with families about SEND*. https://www.tameside.gov.uk/TamesideMBC/media/sen/Tameside-survey-report.pdf
52. Milton Keynes Council. (2017). *SEND Survey report feedback*. https://www.milton-keynes.gov.uk/assets/attach/42222/SEND-survey-reportdec16.pdf
53. Natspec. (2021). *How young people with special educational needs and disabilities find out about their post-16 options*. https://natspec.org.uk/wp-content/uploads/2021/02/IAG-final-report.pdf
54. Norfolk County Council. (2020). *Norfolk SEND Local Area Written Statement of Action 2020-2022*. https://www.Norfolk.gov.uk/-/media/Norfolk/downloads/what-we-do-and-how-we-work/policy-performance-and-partnerships/policies-and-strategies/send-strategy/send-written-statement-of-action.pdf
55. North East Lincolnshire Council. (2021). *SEND Annual Survey Results 2021*. https://sendlocaloffer.nelincs.gov.uk/wp-content/uploads/2021/07/SEND-Survey-2021-CL.pdf
56. North Somerset Council. (2019). North Somerset Short Breaks offer for children and young people with additional needs and disabilities (SEND) under 18 years of age. In. North Somerset: North Somerset Council.
57. North Yorkshire County Council., N. Y. C., Council for Disabled Children. (2021). *North Yorkshire SEND Strategy Development: Analysis of focus groups*. https://www.whatdotheyknow.com/request/nycc_send_strategy_consultation/response/2253941/attach/3/North%20Yorkshire%20SEND%20Strategy%20focus%20groups%20report.pdf.pdf?cookie_passthrough=1
58. Northamptonshire County Council. (2017). Local Offer 2017 Survey. In. Northamptonshire: Northamptonshire County Council.
59. Northumberland County Council. (2020). *Understanding the impact of COVID 19 on children, young people and their families with SEND in Northumberland*. https://www.northumberland.gov.uk/NorthumberlandCountyCouncil/media/Child-Families/SEND/Have%20Your%20Say/SEND-COVID-Survey-Report-July-20.pdf
60. O'Malley, M. (2021). Short Breaks Clubs and activities for children with disabilities (CWD) research: Informing the CWD offer and commissioning strategy. In E. C. Council (Ed.). Essex: Essex County Council.
61. Ofsted. (2021a). *SEND: old issues, new issues, next steps*. https://www.gov.uk/government/publications/send-old-issues-new-issues-next-steps
62. Ofsted. (2021b). *Supporting SEND*. https://www.gov.uk/government/publications/supporting-send/supporting-send
63. Ofsted., C. (2016). *The inspection of local areas’ effectiveness in identifying and meeting the needs of children and young people who have special educational needs and/or disabilities*. https://www.gov.uk/government/consultations/local-area-send-consultation
64. Ofsted., C. (2017). *Local area SEND inspections: one year on*. https://www.gov.uk/government/publications/local-area-send-inspections-one-year-on
65. Oldham County Council. (2022). Independent Consultation for Short Breaks and Social Care Personal Budgets - Oldham. In. Oldham: Action Together.
66. PACTS Stockport parent-carer forum. (2021). *2021 SEND survey*. https://pactstockport.co.uk/wp-content/uploads/2022/02/2021-SEND-Survey-results.pdf
67. Palikara, O., Castro, S., Gaona, C., & Eirinaki, V. (2019). Professionals’ views on the new policy for special educational needs in England: ideology versus implementation. *European Journal of Special Needs Education*, *34*(1), 83-97. https://doi.org/10.1080/08856257.2018.1451310
68. Palikara, O., Castro,S., Gaona,C., Eirinaki,V. (2018). Capturing the Voices of Children in the Education Health and Care Plans: Are We There Yet? [Original Research]. *Frontiers in Education*, *3*. https://doi.org/10.3389/feduc.2018.00024
69. Peterborough City Council. (2020). *SEND College Students Survey*. https://search3.openobjects.com/mediamanager/peterborough/fsd/files/item_4_1_send_student_survey_results.pdf
70. Plymouth City Council. (2019). Local Offer Review Report November 2019. In. Plymouth: Plymouth City Council.
71. Portsmouth City Council. (2021). *Special Educational Needs and Disability (SEND) Parent and Carer Consultation 2021*. Retrieved 31/05/24 from https://yourcityyoursay.portsmouth.gov.uk/special-educational-needs-and-disability-send-consultations/send-parent-and-carer-consultation/
72. Reading Borough Council. (2022). Reading SEND Local Offer Survey 2021 - 2022. In. Reading: Reading Families Forum.
73. Scambler, M. (2021). *East Sussex Children and Young People with Special Educational Needs and Disabilities (SEND)- Comprehensive Needs Assessment 2021*. https://www.eastsussexjsna.org.uk/resources/children-and-young-people-with-special-educational-needs-or-disabilities-send-needs-assessment/
74. Scott, L. (2016). *SEND: The schools and colleges experience*. https://assets.publishing.service.gov.uk/media/5a75a259ed915d6faf2b4805/SEND_experiences_with_schools_and_colleges.pdf
75. Sheffield Parent Carer Forum. (2019). *State of Sheffield 2019*. https://sheffieldparentcarerforum.org.uk/wp-content/uploads/2019/04/State-of-Sheffield-2019-Full-report.pdf
76. Slough Borough Council. (2020). Local offer feedback - Local Offer survey at Haybrook College. In. Slough: Slough Borough Council.
77. Smith, P. (2021). Nottinghamshire County Council SEND Local Offer Survey Report 2020. In. Nottinghamshire: Nottinghamshire County Council.
78. South Gloucestershire Council. (2020). *Quality Assurance Framework Implementation Summary*. https://search3.openobjects.com/mediamanager/southglos/directory/files/send_quality_assurance_framework_summary_report_-_november_2020.pdf
79. South Gloucestershire Council. (2021). Local Offer Annual Report 2020-2021. In: South Gloucestershire Council.
80. St Helens Borough Council. (2017a). Consultation with Children and Young People - The Local Offer & Young People’s Development Session Around the Local Offer 19-25. In: St Helens Borough Council.
81. St Helens Borough Council. (2017b). Local Offer Feedback Session. In: St Helens Borough Council.
82. Staffordshire County Council. (2019). Children and Young People’s Workshop – SEND Local Offer Survey 2019. In: Staffordshire Connects.
83. Staffordshire County Council. (2021). Children and Young People's Survey 2021. In: Staffordshire Connects.
84. Staffordshire County Council. (2022). SEND Local Offer Annual Survey 2022. In: Staffordshire Connects.
85. Stevens, J., Telford & Wrekin Borough Council. (2020). *PODS (PARENTS OPENING DOORS) PARENT CARER FORUM RESPONSES TO ANNUAL SURVEY 2019/2020*. https://www.podstelford.org/wp-content/uploads/2020/03/Annual-Survey-report-Summary-January-2020.pdf
86. Surrey Additional Needs and Disabilities Partnership. (2021). SEND Local Offer Annual Feedback Summary 2021. In: SAND partnership.
87. Swindon Borough Council. (2021a). *SEND Annual Survey 2021*. https://www.swindon.gov.uk/downloads/file/10382/summary_report
88. Swindon Borough Council. (2021b). *SEND Workforce Development Survey 2021: Summary of Results*. https://localoffer.swindon.gov.uk/media/36464/send-workforce-development-survey-2021-summary.pdf
89. Swords, B., Parish N., Kulawik K. (2019). Understanding the drivers for rising demand and associated costs for home-to-school transport. In. Local Government Association Website: Local Goverment Association.
90. Telford and Wrekin Borough Council. (2023). Telford and Wrekin SEND JSNA. In: Telford and Wrekin Borough Council.
91. The National Autistic Society. (2015). *School report 2015*. https://static1.squarespace.com/static/56461310e4b04b5d43f6bb98/t/564de730e4b080a04030c147/1447946032877/SEN-report-branded-280815.pdf
92. Torbay Council. (2015). Local Offer Survey Results (March – April 2015). In: Torbay Council.
93. Torbay Council. (2018a). *SENCO SURVEY 2018 – Summary and Response*. https://search3.openobjects.com/mediamanager/torbay/enterprise/files/sendco_survey_feedback_june_2018_with_response.pdf
94. Torbay Council. (2018b). SENDCO SURVEY - Summary and Response. In: Torbay Council.
95. Torbay Council. (2020). *EHC Plan Questionnaires Sept 16 – March 19*.
96. Vincent, J., Cross,J. (2021). *SEND Consultation Feedback from the 2021 Public Consultation Exercise on Special Educational Needs and/or Disabilities (SEND) Local Area Provision in Sefton*. https://search3.openobjects.com/mediamanager/sefton/fsd/files/send_parent_and_carer_survey_2021_report_1.pdf
97. Wakefield City Council. (2016). Wakefield Local Offer - You Said, We Did. In: Wakefield City Council.
98. Wakefield City Council. (2018). Children and Young People Engagement. In: Wakefield City Council.
99. Westminster City Council. (2017). *Children and Young People with Special Educational Needs and Disabilities Joint Strategic Needs Assessment (JSNA) Report*. https://committees.westminster.gov.uk/documents/s27840/5b%20-%20WCC%20SEND%20JSNA%20report.pdf
100. Westminster City Council. (2021). Autism Strategy Responses and Feedback. In: Westminster City Council.
101. Wiltshire Parent Carer Council. (2018). *SPECIAL SCHOOLS PROVISION IN WILTSHIRE*. https://www.wiltshireparentcarercouncil.co.uk/images/d/df/Special_School_Discussion_Groups_Report_-_Jan_2018_-_Final_Report.pdf
102. Wolverhampton Council. (2019). *Wolverhampton Joint Strategic Needs Assessment*. https://www.wolverhampton.gov.uk/sites/default/files/2020-01/SEND-JSNA.pdf
